# Heterocycle-guided synthesis of *m*-hetarylanilines via three-component benzannulation

**DOI:** 10.3762/bjoc.20.188

**Published:** 2024-09-02

**Authors:** Andrey R Galeev, Maksim V Dmitriev, Alexander S Novikov, Andrey N Maslivets

**Affiliations:** 1 Department of Chemistry, Perm State University, ul. Bukireva 15, Perm, 614990, Russian Federation; 2 Institute of Chemistry, Saint Petersburg State University, Universitetskaya Nab., 7/9, Saint Petersburg, 199034, Russian Federation; 3 Research Institute of Chemistry, Рeoples’ Friendship University of Russia (RUDN University), Miklukho-Maklaya Street, 6, Moscow, 117198, Russian Federation

**Keywords:** aniline, benzannulation, condensation, 1,3-diketone, Hammett constants, terphenyl

## Abstract

A one-pot three-component synthesis of substituted *meta*-hetarylanilines from heterocycle-substituted 1,3-diketones has been developed. The electron-withdrawing power of the heterocyclic substituent (which can be estimated on the basis of calculated Hammett constants) in the 1,3-diketone plays a pivotal role in the studied reaction. The series of *meta*-hetarylanilines prepared (21–85% isolated yield) demonstrates the synthetic utility of the developed method.

## Introduction

**Figure 1:**
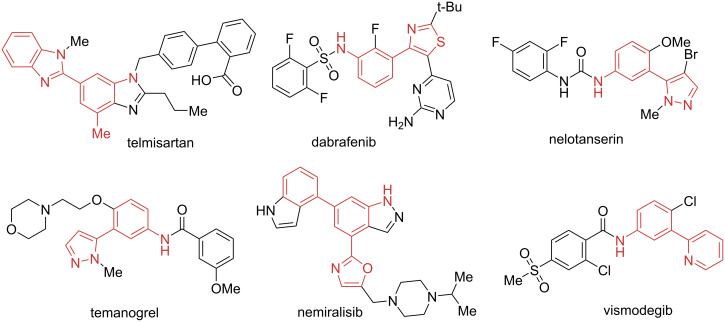
The *meta*-hetarylaniline motif in bioactive molecules.

The aniline moiety is omnipresent in the synthetic chemistry with applications ranging from building blocks to catalysis [1–4]. Among the possible substitution patterns, *meta*-substituted anilines hold a special place. These compounds are difficult to access due to the inherent *ortho*-/*para*-directional reactivity of the amino group, at the same time they are widely used in medicinal chemistry, resulting in several marketed drugs (Figure 1). On the other hand, 3,5-diarylanilines can be regarded as *meta*-terphenyls which are of great interest for material and coordination chemistry [5–16]. Moreover, compounds with diverse bioactivities and natural products contain the *meta*-terphenyl moiety as a key fragment [17–26].

**Scheme 1:**
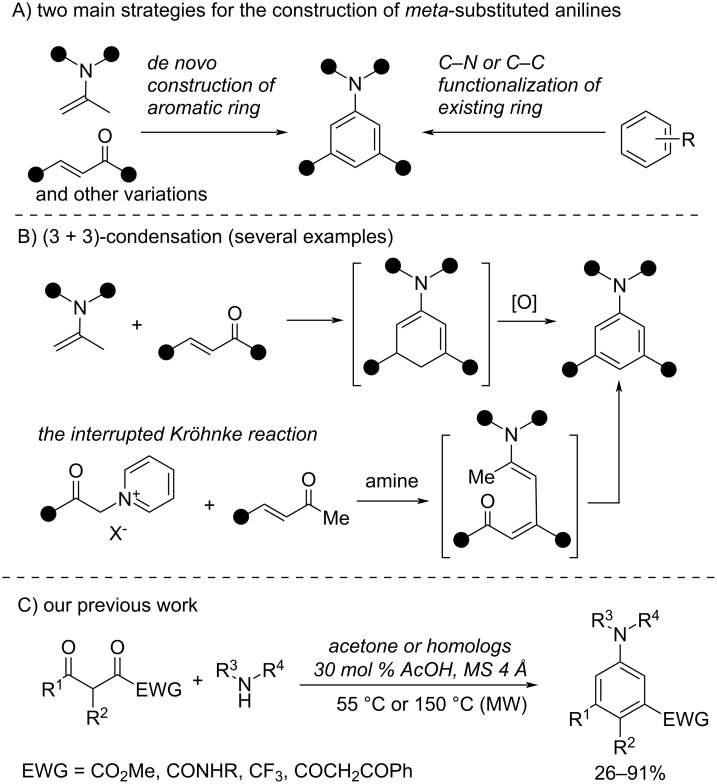
Strategies to access *meta*-substituted anilines.

The aforementioned features have led to an extensive development of novel methods to access *meta*-substituted anilines which can be divided into two main strategies (Scheme 1A) [27]. The first strategy focuses on the decoration of the aromatic ring mainly via metal-catalyzed C–N or C–C bond formation. Despite recent advances in the area of remote C–H functionalization, this strategy still requires some pre-functionalization of the starting material or the use of directing groups [28–32]. An alternative strategy is based on aromatic ring formation via benzannulative inter- or intramolecular condensation of acyclic precursors [27,32–37].

Within the latter strategy, [3 + 3] condensations are gaining much attention due to the availability of starting materials and the straightforward installation of the aryl(hetaryl) substituent in the *meta*-position [38–43]. For example, several methods based on the Michael condensation–oxidation sequence starting from α,β-unsaturated ketones have been described (Scheme 1B) [44–50]. Recently, several methods have been developed based on the interrupted Kröhnke reaction (Scheme 1B) [51–52]. The main step of this process is an intermolecular cyclization of the formed 1,5-diketone followed by aromatization.

Previously we have shown that 1,3-diketones bearing an electron-withdrawing group (EWG) adjacent to one of the carbonyls readily react with in situ-generated acetone imines in a (3 + 3) manner to afford *meta*-substituted anilines (Scheme 1C) [53–54]. Various EWGs (ester, carbamoyl, ketone, trifluoromethyl) have been successfully employed which motivated us to evaluate other possible EWGs.

## Results and Discussion

Based on the fact, that many heterocycles are isoelectronic to an ester or a carbamoyl group, we were interested in testing various heterocycles as electron-withdrawing groups for activation of the carbonyl group in 1,3-diketones. In order to compare the electron-withdrawing ability of heterocycles and previously studied EWGs, we tried to utilize Hammett constants. Since only a few numbers of experimentally measured Hammett constants for heterocycles are known [55–56], this approach seems unsuitable at first. However, recently, a web-based tool for the calculation of the substituent descriptors compatible with the Hammett sigma constants was released [57] allowing direct comparison of different substituents.

**Figure 2:**
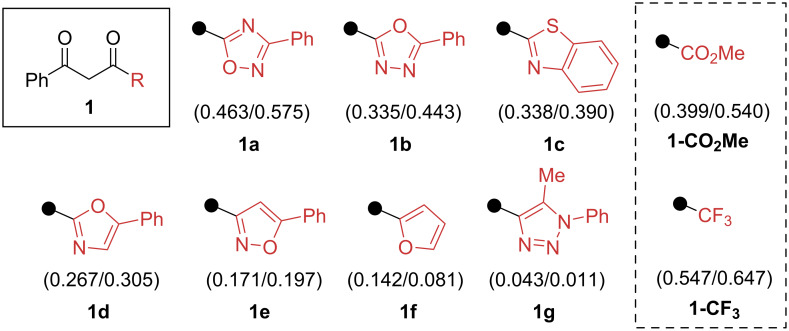
The model series of synthesized 1,3-diketones and corresponding calculated Hammett constants of heterocyclic substituents. Previously studied EWGs [53] are shown in the dashed block. The numbers in parentheses are σ_m_ and σ_p_ calculated [57] constants.

On the basis of the calculated Hammett constants, we have selected a model series of 1,3-diketones, bearing heterocyclic substituents with a range of electron-withdrawing ability (Figure 2). For the amine model series, we used amines with different nucleophilicity, namely benzylamine (primary alkylamine), morpholine (secondary alkylamine) and aniline (aromatic amine).

**Scheme 2:**
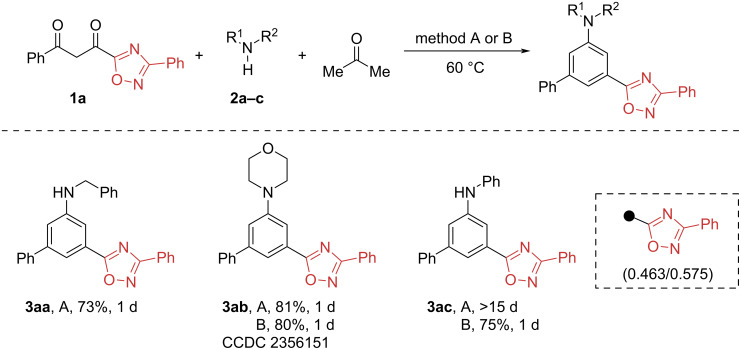
Synthesis of *meta*-substituted anilines from 1,2,4-oxadiazol-5-yl substituted 1,3-diketone **1a**. Conditions A: **1a** (0.5 mmol), **2a,b** (0.5 mmol) and acetone (2 mL), 60 °C. Conditions B: **1a** (0.5 mmol), **2c** (0.75 mmol), AcOH (30 mol %), molecular sieves 3 Å (300 mg) and acetone (2 mL), 60 °C.

We first examined the reaction of 1,2,4-oxadiazole-1,3-diketone **1a** (σ_m_/σ_p_ 0.463/0.575, which is quite close to the constants of the CO_2_Me group, so a successful reaction was expected, Figure 2) with morpholine under previously found conditions (Scheme 2). To our delight, the *meta*-1,2,4-oxadiazole aniline **3ab**, formed by the sequence of aldol-type reactions, was isolated in 80% yield and its structure was confirmed by NMR and single crystal X-ray analysis (CCDC 2356151). No significant improvement in the yield was observed by varying the reaction conditions. Surprisingly, the reaction of 1,3-diketone **1a**, morpholine and acetone without the use of molecular sieves and acid catalysis (conditions A) resulted in 81% yield of *meta*-substituted aniline **3ab**.

Applying the latter conditions to the reaction with benzylamine, the target substituted aniline **3aa** was formed in a good 73% yield. However, the reaction with less nucleophilic aniline was sluggish, requiring almost 15 days to achieve full conversion of the 1,3-diketone **1a** (TLC control). In this case, performing the reaction in the presence of molecular sieves and 1.5-fold excess of aniline (conditions B) dramatically reduced the reaction time to 1 d, allowing isolation of diarylamine **3ac** in 75% yield (Scheme 2).

**Scheme 3:**
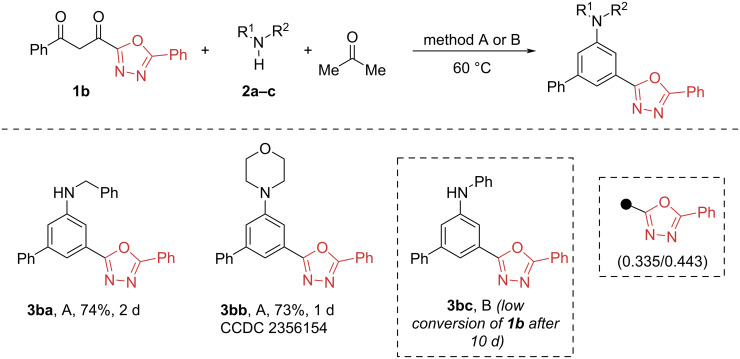
Synthesis of *meta*-substituted anilines from 1,3,4-oxadiazol-substituted 1,3-diketone **1b**. Conditions A: **1b** (0.5 mmol), **2a**,**b** (0.5 mmol) and acetone/CHCl_3_ (3 mL, 2:1), 60 °C. Conditions B: **1b** (0.5 mmol), **2c** (0.75 mmol), AcOH (30 mol %), molecular sieves 3 Å (300 mg) and acetone (2 mL), 60 °C.

Next, we started to screen various heterocyclic 1,3-diketones with the model amine series. The reaction of diketone **1b**, bearing the less electron-withdrawing 1,3,4-oxadiazole moiety (σ_m_/σ_p_ 0.335/0.443), with alkylamines proceeded well (73–74% yields), but required the addition of CHCl_3_ as co-solvent due to the low solubility of the starting 1,3-diketone **1b**. On the other hand, the reaction of 1,3-diketone **1b** with aniline resulted in low conversion of **1b** even at prolonged reaction times (up to 10 days). The addition of molecular sieves, excess aniline, or acid catalysts did not significantly affect the conversion (Scheme 3).

**Scheme 4:**
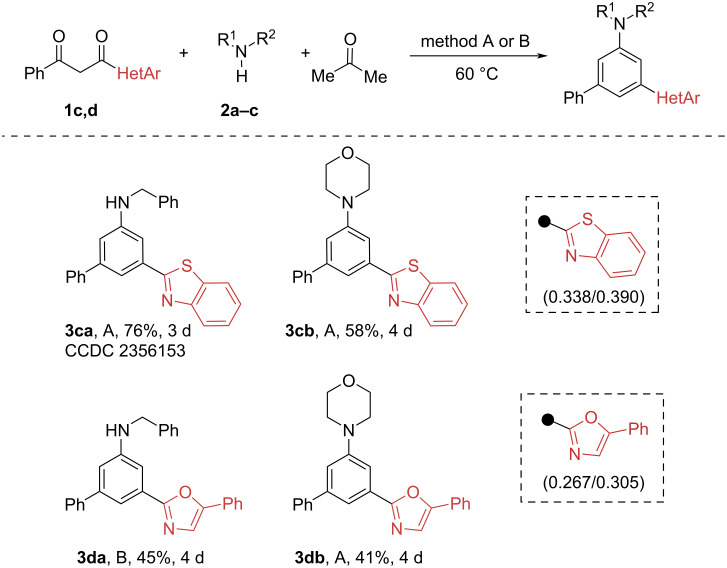
Synthesis of *meta*-substituted anilines from benzothiazol-2-yl and oxazol-2-yl-substituted 1,3-diketones. Conditions A: **1c**,**d** (0.5 mmol), **2a**,**b** (0.75 mmol) and acetone/CHCl_3_ (2 mL, 1:1), 60 °C. Conditions B: **1c**,**d** (0.5 mmol), **2a**,**c** (0.75–2.5 mmol), AcOH (30 mol %), molecular sieves 3 Å (300 mg) and acetone/CHCl_3_ (2 mL, 1:1), 60 °C.

1,3-Diketones with benzothiazole (**1c**, σ_m_/σ_p_ 0.338/0.390) and oxazole (**1d**, σ_m_/σ_p_ 0.267/0.305) substituents reacted with primary and secondary alkylamines, requiring prolonged heating and a 1.5 excess of amine (Scheme 4), to give *meta*-substituted arylamines in reasonable synthetic yields. In the case of 1,3-diketone **1d**, the addition of molecular sieves is necessary in order to reduce the formation of the enamine side-product. Similar to 1,3-diketone **1b**, an extremely low conversion of **1c** and **1d** was observed in the reaction with aniline.

**Scheme 5:**
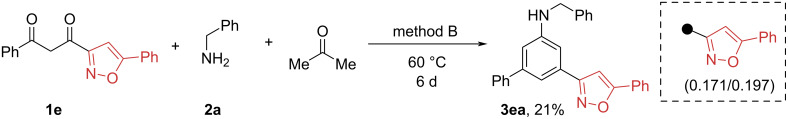
Synthesis of *meta*-substituted aniline from isoxazol-3-yl-substituted 1,3-diketone **1e**. Conditions B: **1e** (0.3 mmol), **2a** (0.45 mmol), AcOH (30 mol %), molecular sieves 3 Å (300 mg) and acetone (1 mL), 60 °C.

The isoxazol-3-yl or furan-2-yl substituents have calculated Hammett constants below 0.200 (Figure 2), thus based on the above observations, a slow reaction of 1,3-diketones **1e** and **1f** with aliphatic amines was expected. Indeed, the reaction of 1,3-diketones **1e** or **1f** with benzylamine was sluggish, and a number of undefined side-products were formed (LC control). *m*-Isoxazole arylamine **3ea** was isolated in low yield (21%) after heating for 6 days (Scheme 5). In fact, arylamine **3fa**, produced from furan-substituted 1,3-diketone **1f** and a 5-fold excess of benzylamine, could be prepared in 18% crude yield (after 7 days, see Supporting Information File 1, page S9).

Finally, all attempts to perform the reaction with 1,2,3-triazole 1,3-diketone **1g** (σ_m_/σ_p_ 0.043/0.011) with model amine series failed (7 days reaction time in each case). This result is in good agreement with low Hammett constants of the triazole-substituent of 1,3-diketone **1g**, which are close to the calculated Hammett constants of the phenyl group (σ_m_/σ_p_ 0.055/0.012).

Quantum-chemical/chemoinformatics calculations were also performed to correlate the observed reactivity with the charges on the carbonyl group of 1,3-diketones. Unfortunately, no reliable correlation was found between the charges and the reactivity of the 1,3-diketones with the present set of heterocyclic substituents or with the EWGs that were previously studied (see Supporting Information File 1).

**Figure 3:**
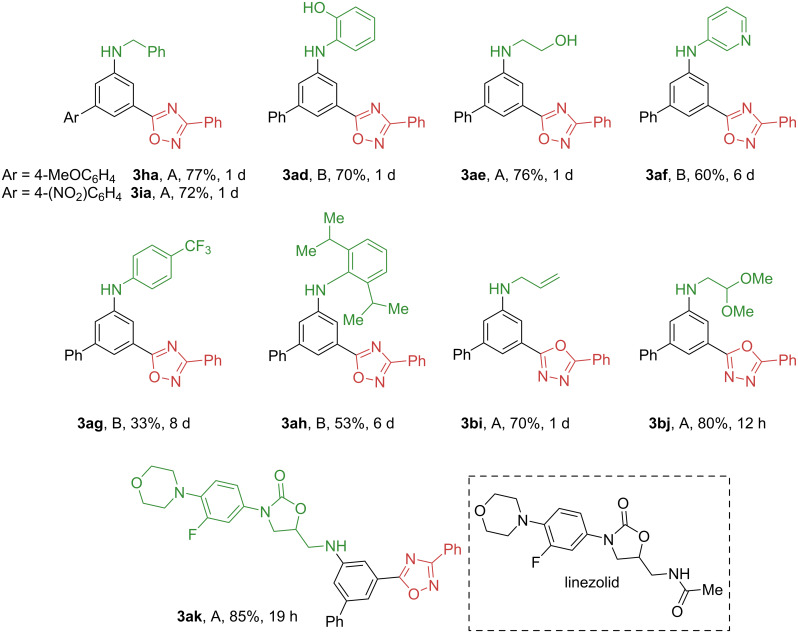
Scope of functionalized amines in three-component condensation. Conditions A: **1a**,**b**,**h**,**i** (0.2–0.5 mmol), **2e**,**i**–**k** (0.2–0.75 mmol) and acetone (1–2 mL) or acetone/CHCl_3_ (3 mL, 2:1), 60 °C. Conditions B: **1a** (0.3–0.5 mmol), **2d**,**f**–**h** (0.45–0.75 mmol), AcOH (30 mol %), molecular sieves 3 Å (300 mg) and acetone (1–2 mL), 60 °C.

The presence of electron-donating (**3ha**) or electron-withdrawing groups (**3ia**) in the aryl substituent of the 1,3-diketone does not affect the reaction outcome (Figure 3). We have further demonstrated the utility of three-component condensation by introducing additional functional groups into the amine moiety (Figure 3).

Substituted arylamines bearing alcohol (**3ae**), phenol (**3ad**), alkene (**3bi**), dimethyl acetal (**3bj**) functionality can be accessed in good yields. Reaction of 1,3-diketone **1a** with a non-amidine type heterocyclic amine, 3-aminopyridine, provided *N*-hetarylaniline (**3af**) in moderate yield. Furthermore, it is possible to synthesize sterically hindered anilines such as arylamine **3ah**, which was prepared from 2,6-di(isopropyl)aniline in 53% yield. However, in the case of an electron-deficient amine (4-trifluoromethylaniline), the desired *meta*-heterocycle aniline **3ag** was prepared in low yield. Finally, the developed method is suitable for the late-stage arylation of drug-like molecules such as deacetyllinezolid (**3ak**).

**Scheme 6:**
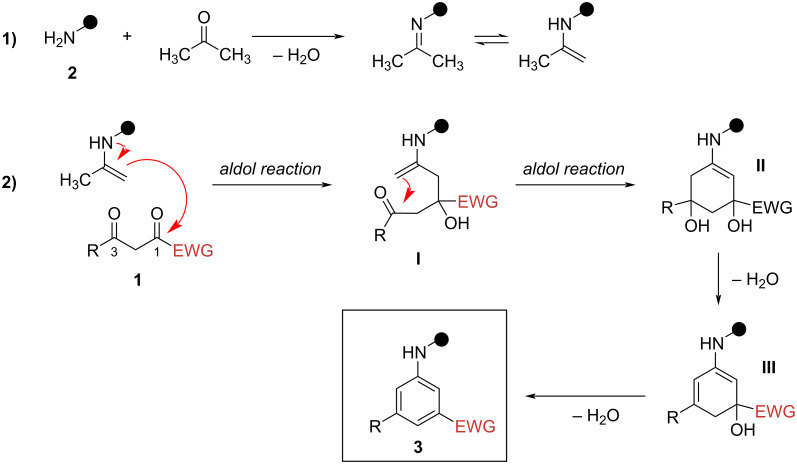
Proposed mechanism for the formation of *meta*-substituted anilines **3** via three-component benzannulation.

The *meta*-substituted anilines **3** are formed in a sequence of reactions shown in Scheme 6 [53]. Firstly, the reaction of acetone and amines **2** leads to the formation of acetone imine/enamine (reaction 1, Scheme 6). Nucleophilic addition of an enamine to the most electron-deficient carbonyl group (C^1^=O, adjacent to the EWG) of the 1,3-diketones **1** gives the acyclic carbinol **I** (reaction 2, Scheme 6), followed by the intramolecular addition of enamine **I** to the C^3^=O to form intermediate **II**, which dehydrates to cyclic carbinol **III**. Finally, dehydration of intermediate **III** yields anilines **3**.

## Conclusion

In summary, a method for the synthesis of substituted *meta*-hetarylanilines under mild conditions starting from 1,3-diketones with heterocyclic substituents, acetone and various amines has been developed. The success of this three-component reaction is governed by the electron-withdrawing ability of the heterocyclic substituent in the 1,3-diketone, which can be evaluated with computational Hammett constants. As a rule-of-thumb, 1,3-diketones bearing substituents with σ_m_ or σ_p_ > 0.300 afford *meta*-anilines from alkylamines in good synthetic yields, and higher σ_m_ or σ_p_ are required for three-component condensation with less nucleophilic arylamines. The developed one-pot three-component reaction is efficient (yields up to 85%), compatible with many functional groups, and allows to synthesize a series of difficult-to-access *meta*-substituted anilines of interest for medicinal and material chemistry.

## Supporting Information

CCDC 2356152 (**1g**), 2356151 (**3ab**), 2356154 (**3bb**) and 2356153 (**3ca**) contain the supplementary crystallographic data for this paper. These data can be obtained free of charge from the Cambridge Crystallographic Data Centre via https://www.ccdc.cam.ac.uk/structures.

File 1 Full experimental details, characterization data of new compounds and NMR spectra.

File 2 xyz files with Cartesian atomic coordinates for all model structures and CIF-files for compounds **1g**, **3ab**, **3bb** and **3ca**.

## Data Availability

The data that supports the findings of this study is available from the corresponding author upon reasonable request.
