# Supplementary material for: Heterocycle-guided synthesis of m-hetarylanilines via three-component benzannulation
Source: Beilstein J Org Chem. 2024 Sep 2;20:2208–16. doi: 10.3762/bjoc.20.188 (PMC11403807; doi:10.3762/bjoc.20.188)
Supplement: File 1 — Full experimental details, characterization data of new compounds and NMR spectra. [file Beilstein_J_Org_Chem-20-2208-s001.pdf]

BEILSTEIN JOURNAL OF ORGANIC CHEMISTRY

Supporting Information

for

Heterocycle-guided synthesis of *m*-hetarylanilines via three-component benzannulation

Andrey R. Galeev, Maksim V. Dmitriev, Alexander S. Novikov and Andrey N. Maslivets

Beilstein J. Org. Chem. **2024,** *20,* 2208–2216. doi:10.3762/bjoc.20.188

Full experimental details, characterization data of new compounds and NMR spectra

License and Terms: This is a supporting information file under the terms of the Creative Commons Attribution License (https://creativecommons.org/licenses/by/4.0). Please note that the reuse, redistribution and reproduction in particular requires that the author(s) and source are credited and that individual graphics may be subject to special legal provisions.
The license is subject to the *Beilstein Journal of Organic Chemistry* terms and conditions: (https://www.beilstein-journals.org/bjoc/terms)

General information

^{1}H, ^{13}C and ^{19}F NMR spectra were recorded on a Bruker Avance III HD spectrometer (at 400, 101, 377 MHz, respectively) at 40 °C (313 K) in $CDCl_3$ and DMSO-d_6 using the residual solvent peak ($CDCl_3$: δH = 7.26 ppm, δC = 77.16 ppm; DMSO-d_6: δH = 2.50 ppm; δC = 39.52 ppm) as internal standards, for ^{19}F spectra C_6F_6 (δ = −164.9 ppm) was used as the internal standart. Splitting patterns of apparent multiplets were designated as s (singlet), d (doublet), t (triplet), q (quartet), m (multiplet), br (broadened). FTIR spectra were recorded on a Perkin–Elmer Spectrum Two spectrometer from mulls in mineral oil. Melting points were measured with Mettler Toledo MP70 Melting Point apparatus or Khimlabpribor PTP apparatus. Thin-layer chromatography (TLC) was performed on silica gel 60 F254 plates (Merck); spots were visualized with UV light (254 nm) or iodine vapors. HPLC analyses were performed on Hitachi Chromaster equipped with PDA detector Hitachi Chromaster 5430 (NUCLEODUR C18 Gravity column 3 µm, 4 × 150 mm). Elemental analyses were carried out on a Vario MICRO Cube analyzer. X-ray structural analyses were performed on an Xcalibur Ruby diffractometer using a Mo X-ray source (Mo Kα 0.71073 Å), by scanning at 295(2) K. All solvents and reagents were purchased from commercial vendors and were used as received (PhMe and 1,4-dioxane were dried over Na and distilled).

Synthesis of starting materials

General procedure for the synthesis of 1,2,4-oxadiazole 1,3-diketones 1a, 1h and 1i [1,2]

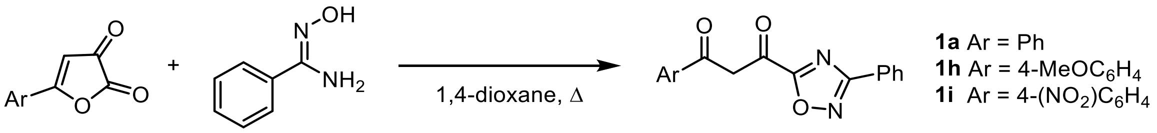

Benzamidoxime (1 equiv.) was added to a solution of 5-arylfuran-2,3-dione (1 equiv.) in dry 1,4-dioxane (10 mL/ 5 mmol of furan-2,3-dione) and the mixture was refluxed for 1 hour. The mixture was cooled, the solvent was evaporated and the residue was crystallized from EtOH.

1-Phenyl-3-(3-phenyl-1,2,4-oxadiazol-5-yl)propane-1,3-dione (1a). Yield 73%, beige powder, mp 132–133 °C (lit. 134–135 °C), R_f 0.72 (PhMe/EtOAc 5:1). **^{1}H NMR** (400 MHz, $CDCl_3$) δ 15.63 (s, 1H), 8.18 (dd, *J* = 7.7, 1.9 Hz, 2H), 8.10 – 8.01 (m, 2H), 7.64 (t, *J* = 7.3 Hz, 1H), 7.58 – 7.48 (m, 5H), 7.30 (s, 1H). **^{13}C NMR** (101 MHz, $CDCl_3$) δ 189.1, 171.3, 169.5, 167.9, 134.4, 134.1, 131.8, 129.15 (2C), 129.12 (2C), 128.1 (2C), 127.8 (2C), 126.3, 97.8.

1-(4-Methoxyphenyl)-3-(3-phenyl-1,2,4-oxadiazol-5-yl)propane-1,3-dione (1h). Yield 68%, yellow powder, mp 133–134 °C (lit. 132–133 °C), R_f 0.79 (PhMe/EtOAc 5:1). **^{1}H NMR** (400 MHz, $CDCl_3$) δ 15.80 (br.s, 1H), 8.19 – 8.15 (m, 2H), 8.04 (d, *J* = 9.0 Hz, 2H), 7.58 – 7.48 (m, 3H), 7.22 (s, 1H), 7.01 (d, *J* = 9.0 Hz, 2H), 3.90 (s, 3H). **^{13}C NMR** (101 MHz, $CDCl_3$) δ 188.9, 171.6, 169.5, 166.3, 164.8, 131.8, 130.5 (2C), 129.1 (2C), 127.8 (2C), 127.1, 126.3, 114.5 (2C), 97.5, 55.8.

1-(4-Nitrophenyl)-3-(3-phenyl-1,2,4-oxadiazol-5-yl)propane-1,3-dione (1i). Yield 60%, tan powder, mp 142–146 °C, R_f 0.45 (PhMe/EtOAc 5:1). **^{1}H NMR** (400 MHz, $CDCl_3$) δ 14.71 (br.s, 1H), 8.37 (d, *J* = 8.9 Hz, 2H), 8.20 (d, *J* = 8.9 Hz, 2H), 8.18 – 8.11 (m, 2H), 7.62 – 7.48 (m, 3H), 7.33 (s, 1H). **^{13}C NMR** (101 MHz, $CDCl_3$) δ 185.2, 170.8, 170.1, 169.7, 150.9, 139.3, 132.0, 129.2 (2C), 128.9 (2C), 127.8 (2C), 126.0, 124.2 (2C), 98.3.

1-Phenyl-3-(5-phenyl-1,3,4-oxadiazol-2-yl)propane-1,3-dione (1b)

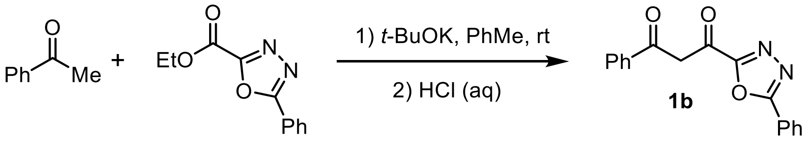

t-BuOK (1.68 g, 15 mmol, in two portions) was added to a solution of ethyl 5-phenyl-1,3,4-oxadiazole-2-carboxylate [3] (2.29 g, 10 mmol) and acetophenone (1.16 mL, 10 mmol) in 30 mL of dry PhMe. The mixture was stirred at rt for 1 day (color change from colorless to yellow-orange/reddish slurry). 20 mL of 1 M HCl was added to the mixture and stirred for 30 min, followed by extraction with $CHCl_3$ (2 × 50 mL). The combined organic extracts were washed with water (2x20 mL) and evaporated (without drying), the residue was crystallized from EtOH. Yield 1.28 g (44%), bright-yellow cotton-like solid, mp 209–210 °C, R_f 0.49 (PhMe/EtOAc 5:1).

^{1}H NMR (400 MHz, $CDCl_3$) δ 15.57 (s, 1H), 8.27 – 8.14 (m, 2H), 8.07 – 7.93 (m, 2H), 7.71 – 7.42 (m, 6H), 7.30 (s, 1H). **^{13}C NMR** (101 MHz, $CDCl_3$) δ 184.7, 172.5, 166.5, 161.4, 133.7, 133.7, 132.8, 129.4 (2C), 129.1 (2C), 127.8 (2C), 127.7 (2C), 123.2, 96.1. Anal. Calcd for $C_{17}H_{12}N_2O_3$: C 69.86; H 4.14; N 9.58. Found: C 70.05; H 4.34; N 9.39.

1-(Benzo[d]thiazol-2-yl)-3-phenylpropane-1,3-dione (1c) [4]

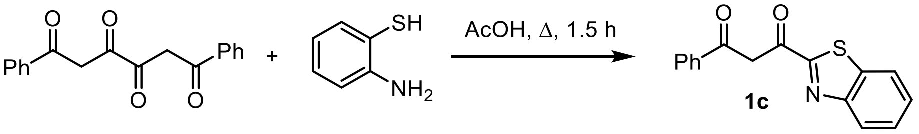

A solution of 1,6-diphenylhexane-1,3,4,6-tetraone (2.0 g, 6.8 mmol) and *o*-aminothiophenol (710 uL, 6.8 mmol) in 20 mL of glacial acetic acid was refluxed for 1.5 h. The mixture was cooled and left overnight for crystallization. The formed participate was filtered and recrystallized from AcOH. Yield 1.07 g (56%), mp 152–154 °C (lit. 153–155 °C).

1-Phenyl-3-(5-phenyloxazol-2-yl)propane-1,3-dione (1d)

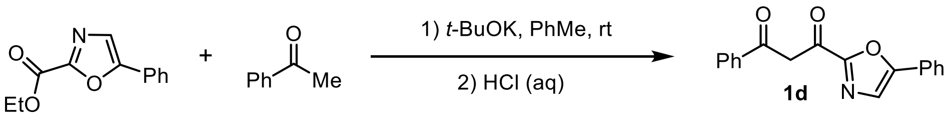

t-BuOK (1.22 g, 11 mmol, in one portion) was added to a solution of ethyl 5-phenyloxazole-2-carboxylate [5] (1.58 g, 7.3 mmol) and acetophenone (850 uL, 7.3 mmol) in 30 mL dry PhMe. The mixture was stirred at rt for 5 h, then 1.5 mL AcOH was added and the mixture was stirred for another 30 min. The solvent was evaporated and the residue was crystallized from EtOH/H_2O. Yield 1.42 g (67%), bright-yellow cotton-like solid, mp 176–178 °C.

^{1}H NMR (400 MHz, $CDCl_3$) δ 15.81 (s, 1H), 8.10 – 7.94 (m, 2H), 7.86 – 7.73 (m, 2H), 7.61 – 7.54 (m, 2H), 7.53 – 7.44 (m, 4H), 7.41 (t, *J* = 7.3 Hz, 1H), 7.20 (s, 1H). **^{13}C NMR** (101 MHz, $CDCl_3$) δ 183.9, 174.2, 157.2, 154.4, 134.3, 133.1, 129.9, 129.3 (2C), 128.9 (2C), 127.5 (2C), 127.1, 125.3 (2C), 124.5, 95.1. Anal. Calcd for $C_{18}H_{13}NO_3$: C 74.22; H 4.50; N 4.81. Found: C 74.67; H 4.55; N 5.02.

1-Phenyl-3-(5-phenylisoxazol-3-yl)propane-1,3-dione (1e)

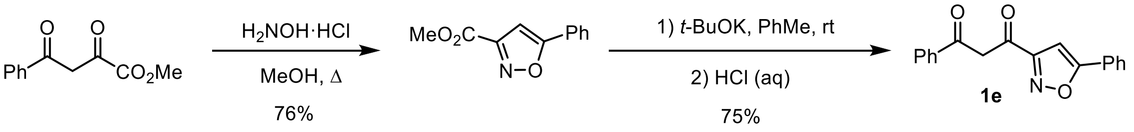

Synthesis of *methyl 5-phenylisoxazole-3-carboxylate*: hydroxylamine hydrochloride (2.52 g, 36 mmol) was added to a solution of methyl benzoylpyruvate (6.18 g, 30 mmol) in 30 mL of MeOH. The mixture refluxed for 3 h, cooled to rt and placed in a refrigerator for 2 h. The formed precipitate was filtered and washed with a small amount of cooled MeOH. Yield 4.63 g (76%), off-white powder, mp 79–81 °C (lit. 80–82 °C [Synthesis, **2013**, 45(2), 260–4]).

t-BuOK (1.34 g, 12 mmol, in one portion) was added to a solution of methyl 5-phenylisoxazole-3-carboxylate (2.03 g, 10 mmol) and acetophenone (1170 uL, 10 mmol) in 40 mL of dry PhMe. The mixture was stirred at rt for 1 day (color change from colorless to yellow-orange). 20 mL of 1M HCl and 20 mL of H_2O were added to the mixture, the resulting mixture was stirred for 30 min, the phases were separated and the aqueous phase was extracted with PhMe (20 mL). The combined organic extracts were washed with water (2 × 30 mL) and evaporated (without drying), the residue was crystallized from EtOH/CH_2Cl_2. Yield 2.19 g (75%), off-white cotton-like solid, mp 138–139 °C, R_f 0.84 (PhMe/EtOAc 5:1).

^{1}H NMR (400 MHz, $CDCl_3$) δ 15.86 (s, 1H), 8.04 – 7.97 (m, 2H), 7.87 – 7.74 (m, 2H), 7.57 (t, *J* = 7.3 Hz, 1H), 7.53 – 7.43 (m, 5H), 7.13 (s, 1H), 6.98 (s, 1H). **^{13}C NMR** (101 MHz, $CDCl_3$) δ 184.2, 179.2, 171.7, 161.8, 134.3, 133.1, 130.8, 129.2 (2C), 128.9 (2C), 127.5 (2C), 127.0, 126.1 (2C), 98.3, 95.1. Anal. Calcd for $C_{18}H_{13}NO_3$: C 74.22; H 4.50; N 4.81. Found: C 74.54; H 4.73; N 5.01.

1-(Furan-2-yl)-3-phenylpropane-1,3-dione (1f)

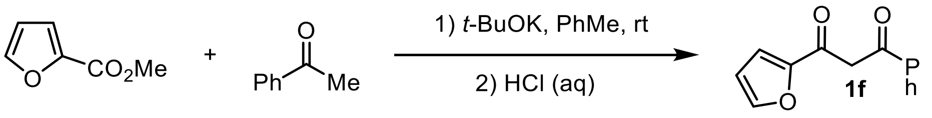

Similar to the literature method [6]: *t*-BuOK (3.2 g, 28.5 mmol, in one portion) was added to a solution of methyl furan-2-carboxylate (2 mL, 19 mmol) and acetophenone (2.2 mL, 19 mmol) in 30 mL dry PhMe. The mixture was stirred at rt for 1 day (color change from colorless to orange). 3.2 mL AcOH and 50 mL H_2O were added to the mixture and stirred for 1 hour. The phases were separated and the aqueous phase was extracted with EtOAc (2 × 25 mL). The combined organic phase was washed with water (2 × 50 mL) and evaporated to give a yellow-orange oil. EtOH was added to the oil and the resulting solution was placed in a freezer overnight. The precipitate was filtered and washed with a small amount of cooled EtOH and hexane. Yield 1.6 g (40%), beige solid, mp 66–68 °C (lit. 66 °C [7]), R_f 0.74 (PhMe/EtOAc 5:1).

^{1}H NMR (400 MHz, $CDCl_3$) δ 16.12 (s, 1H), 8.01 – 7.85 (m, 2H), 7.63 – 7.59 (m, 1H), 7.54 (t, *J* = 7.3 Hz, 1H), 7.48 (t, *J* = 7.3 Hz, 2H), 7.24 (d, *J* = 3.5 Hz, 1H), 6.77 (s, 1H), 6.58 (dd, *J* = 3.5, 1.7 Hz, 1H). **^{13}C NMR** (101 MHz, $CDCl_3$) δ 182.9, 177.6, 151.3, 146.2, 135.0, 132.5, 128.8 (2C), 127.2 (2C), 115.8, 112.8, 92.9.

1-(5-Methyl-1-phenyl-1*H*-1,2,3-triazol-4-yl)-3-phenylpropane-1,3-dione (1g)

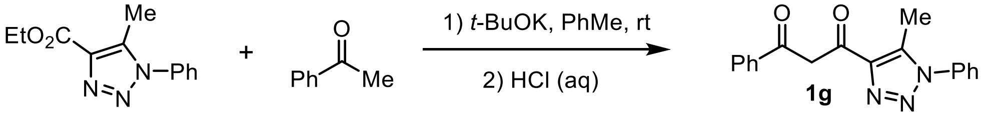

t-BuOK (850 mg, 7.55 mmol, in one portion) was added to a solution of ethyl ethyl 5-methyl-1-phenyl-1*H*-1,2,3-triazole-4-carboxylate [8] (1.16 g, 5 mmol) and acetophenone (595 uL, 5 mmol) in 10 mL of dry PhMe. The mixture was stirred at rt for 1 day (color change from colorless to orange-red). 10 mL of 1M HCl and 20 mL of H_2O were added to the mixture and stirred for 30 min, followed by extraction with EtOAc (2 × 30 mL). The combined organic extracts were washed with water (2 × 20 mL) and evaporated (without drying), the residue was crystallized from EtOH/H_2O, filtered and washed with EtOH/H_2O and hexane. Yield 672 mg (44%), beige powder, mp 114–116 °C.

^{1}H NMR (400 MHz, $CDCl_3$, enol OH not found) δ 8.06 – 8.00 (m, 2H), 7.65 – 7.55 (m, 3H), 7.54 – 7.44 (m, 5H), 7.40 (s, 1H), 2.68 (s, 3H). **^{13}C NMR** (101 MHz, $CDCl_3$) δ 184.4, 181.2, 142.0, 137.4, 135.7, 134.7, 132.4, 130.1, 129.8 (2C), 128.8 (2C), 127.2 (2C), 125.5 (2C), 94.8, 10.4. Anal. Calcd for $C_{18}H_{15}N_3O_2$: C 70.81; H 4.95; N 13.76. Found: C 71.08; H 5.17; N 13.87.

Synthesis of products 3

General procedure A: 1,3-Diketone **1a–g**, acetone (and chloroform if necessary) and amine (1.1 or 1.5 equiv) were added to a reaction vial. The vial was placed in a preheated (60 °C, thermocouple) heating block and stirred for the indicated time. After completion, the reaction mixture was filtered to remove molecular sieves and purified by recrystallization or column chromatography.

General procedure B: 1,3-Diketone **1a–g**, acetone (and chloroform if necessary), molecular sieves 3 Å (300 mg per 0.5 mmol of diketone), acetic acid (30 mol %) and amine were added to a reaction vial. The vial was placed in a preheated (60 °C, thermocouple) heating block and stirred for the indicated time. After competition, the reaction mixture was filtered to remove molecular sieves and purified by recrystallization or column chromatography.

***N*-Benzyl-5-(3-phenyl-1,2,4-oxadiazol-5-yl)-[1,1'-biphenyl]-3-amine (3aa)**

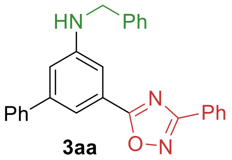

3aa

According to the **GP A**; from 1,3-diketone **1a** (0.5 mmol), amine **2a** (0.55 mmol) and acetone (2 mL); reaction time 1 d, recrystallized from EtOH. Beige powder, 148 mg (73%), mp 151–153 °C, R_f = 0.64 (PhMe).

^{1}H NMR (400 MHz, $CDCl_3$) δ 8.26 – 8.11 (m, 2H), 7.83 (d, *J* = 1.6 Hz, 1H), 7.61 (dd, *J* = 7.2, 1.9 Hz, 2H), 7.53 – 7.50 (m, 4H), 7.48 – 7.28 (m, 8H), 7.08 (t, *J* = 1.9 Hz, 1H), 5.03 (s, 1H), 4.48 (s, 2H). **^{13}C NMR** (101 MHz, $CDCl_3$) δ 176.2, 169.1, 148.5, 143.6, 140.5, 138.4, 131.2, 129.97 (4C), 129.95 (2C), 128.0, 127.9 (2C), 127.8, 127.7 (2C), 127.3 (3C), 125.8, 117.0, 116.4, 111.3, 48.9. IR (mineral oil), cm^{-1}: 3432, 1613, 1581, 1562, 1464, 1455, 1445. Anal. Calcd for $C_{27}H_{21}N_3O$: C 80.37; H 5.25; N 10.41. Found: C 80.60; H 5.44; N 10.51.

4-(5-(3-Phenyl-1,2,4-oxadiazol-5-yl)-[1,1'-biphenyl]-3-yl)morpholine (3ab)

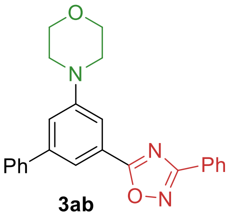

3ab

According to the **GP A**; from 1,3-diketone **1a** (0.5 mmol), amine **2b** (0.55 mmol) and acetone (2 mL); reaction time 1 d, recrystallized from EtOH. Beige powder, 156 mg (81%), mp 172–174 °C, R_f = 0.6 (PhMe/EtOAc 5:1).

^{1}H NMR (400 MHz, $CDCl_3$) δ 8.23 – 8.16 (m, 2H), 7.95 (t, *J* = 1.5 Hz, 1H), 7.73 (dd, *J* = 2.5, 1.4 Hz, 1H), 7.67 – 7.63 (m, 2H), 7.55 – 7.45 (m, 5H), 7.44 – 7.37 (m, 1H), 7.35 (t, *J* = 2.0 Hz, 1H), 4.00 – 3.88 (m, 4H), 3.43 – 3.29 (m, 4H).**^{13}C NMR** (101 MHz, $CDCl_3$) δ 176.1, 169.2, 152.1, 143.6, 140.6, 131.3, 129.0 (2C), 128.97 (2C), 128.1, 127.7 (2C), 127.4 (2C), 127.2, 125.7, 118.8, 118.7, 113.8, 66.9 (2C), 49.3 (2C). IR (mineral oil), cm^{-1}: 1581, 1589, 1556, 1462, 1447. Anal. Calcd for $C_{24}H_{21}N_3O_2$: C 75.18; H 5.52; N 10.96. Found: C 75.43; H 5.71; N 10.78.

***N*-Phenyl-5-(3-phenyl-1,2,4-oxadiazol-5-yl)-[1,1'-biphenyl]-3-amine (3ac)**

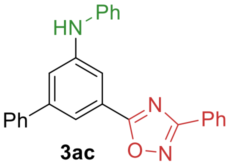

3ac

According to the **GP B**; from 1,3-diketone **1a** (0.5 mmol), amine **2c** (0.55 mmol) and acetone (2 mL); reaction time 1 d, flash column chromatography (PhMe/hexane 2:1). Off-white wool-like compound, 146 mg. (75%), mp 164–165 °C, R_f = 0.3 (PhMe/hexane 2:1).

^{1}H NMR (400 MHz, $CDCl_3$) δ 8.24 – 8.12 (m, 2H), 7.98 (t, *J* = 1.5 Hz, 1H), 7.85 (t, *J* = 1.8 Hz, 1H), 7.67 – 7.60 (m, 2H), 7.55 – 7.44 (m, 6H), 7.44 – 7.32 (m, 3H), 7.21 (d, *J* = 7.3 Hz, 2H), 7.06 (t, *J* = 7.4 Hz, 1H), 5.62 (s, 1H).**^{13}C NMR** (101 MHz, $CDCl_3$) δ 175.9, 169.2, 144.9, 143.7, 142.1, 140.1, 131.3, 129.8 (2C), 129.05 (2C), 129.0 (2C), 128.2, 127.7 (2C), 127.3 (2C), 127.2, 126.0, 122.6, 119.6, 119.4 (2C), 119.3, 115.1. IR (mineral oil), cm^{-1}: 3324,

1591, 1561, 1525, 1496, 1462. Anal. Calcd for $C_{26}H_{19}N_3O$: C 80.18; H 4.92; N 10.79. Found: C 80.30; H 5.05; N 10.86.

N-Benzyl-5-(5-phenyl-1,3,4-oxadiazol-2-yl)-[1,1'-biphenyl]-3-amine (3ba)

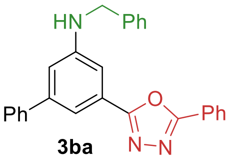

3ba

According to the **GP A**; from 1,3-diketone **1b** (0.5 mmol), amine **2a** (0.55 mmol), acetone (2 mL) and $CHCl_3$ (1 mL); reaction time 2 d, recrystallized from EtOH. Off-white compound, 149 mg (74%), mp 198–200 °C, R_f = 0.54 (PhMe/EtOAc 5:1).

^{1}H NMR (400 MHz, DMSO-d_6) δ 8.18 – 8.08 (m, 2H), 7.69 – 7.60 (m, 5H), 7.52 (d, J = 1.6 Hz, 1H), 7.51 – 7.42 (m, 4H), 7.42 – 7.32 (m, 4H), 7.25 (t, J = 7.3 Hz, 1H), 7.10 (t, J = 1.9 Hz, 1H), 6.78 (t, J = 6.0 Hz, 1H), 4.45 (d, J = 5.9 Hz, 2H). **^{13}C NMR** (101 MHz, DMSO-d_6) δ 164.4, 163.7, 149.6, 142.0, 139.8, 139.6, 131.8, 129.3 (2C), 128.8 (2C), 128.3 (2C), 127.7, 127.2 (2C), 126.7, 126.6 (2C), 126.5 (2C), 124.3, 123.3, 113.8, 112.4, 109.0, 46.3. IR (mineral oil), cm^{-1}: 3340, 1601, 1579, 1551, 1462. Anal. Calcd for $C_{27}H_{21}N_3O$: C 80.37; H 5.25; N 10.41. Found: C 80.75; H 5.18; N 10.58.

4-(5-(5-Phenyl-1,3,4-oxadiazol-2-yl)-[1,1'-biphenyl]-3-yl)morpholine (3bb)

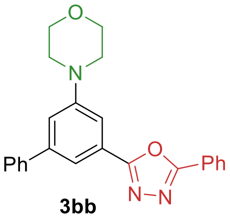

3bb

According to the **GP A**; from 1,3-diketone **1b** (0.5 mmol), amine **2b** (0.55 mmol), molecular sieves 3Å (300 mg), acetone (2 mL) and $CHCl_3$ (1 mL); reaction time 1 d, recrystallized from EtOH/Me_2CO. Beige powder, 140 mg (73%), mp 203–205 °C, R_f = 0.26 (PhMe/EtOAc 5:1).

^{1}H NMR (400 MHz, $CDCl_3$) δ 8.21 – 8.11 (m, 2H), 7.82 (t, J = 1.5 Hz, 1H), 7.68 (dd, J = 2.5, 1.4 Hz, 1H), 7.66 – 7.61 (m, 2H), 7.59 – 7.51 (m, 3H), 7.48 (t, J = 7.4 Hz, 2H), 7.40 (t, J = 7.3 Hz, 1H), 7.27 (dd, J = 2.5, 1.5 Hz, 1H), 4.39 – 3.71 (m, 4H), 3.61 – 3.03 (m, 4H). **^{13}C NMR** (101 MHz, $CDCl_3$) δ 165.1, 164.8, 152.2, 143.6, 140.8, 131.8, 129.2 (2C), 129.0 (2C), 128.1, 127.4 (2C), 127.1 (2C), 125.4, 124.2, 117.8, 117.4, 112.6, 66.9 (2C), 49.3 (2C). IR (mineral oil), cm^{-1}: 1595, 1588, 1574, 1540, 1487, 1462. Anal. Calcd for $C_{24}H_{21}N_3O_2$: C 75.18; H 5.52; N 10.96. Found: C 75.61; H 5.32; N 11.13.

5-(Benzo[*d*]thiazol-2-yl)-*N*-benzyl-[1,1'-biphenyl]-3-amine (3ca)

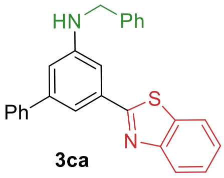

3ca

According to the **GP A**; from 1,3-diketone **1c** (0.5 mmol), amine **2a** (0.75 mmol), acetone (1 mL) and $CHCl_3$ (1 mL); reaction time 3 d, recrystallized from EtOH. Light-yellow powder, 148 mg (76%), mp 143–145 °C, R_f = 0.26 (Hexane/EtOAc 5:1).

^{1}H NMR (400 MHz, $CDCl_3$) δ 8.09 (d, J = 8.2 Hz, 1H), 7.90 (dd, J = 8.1, 1.1 Hz, 1H), 7.67 (t, J = 1.5 Hz, 1H), 7.65 – 7.60 (m, 2H), 7.53 – 7.41 (m, 6H), 7.41 – 7.34 (m, 4H), 7.34 – 7.28 (m, 1H), 6.99 (t, J = 1.9 Hz, 1H), 4.48 (s, 2H), 4.36 (br. s, 1H). **^{13}C NMR** (101 MHz, $CDCl_3$) δ 168.6, 154.3, 148.6, 143.4, 141.0, 138.8, 135.3, 135.1, 128.91 (2C), 128.86 (2C), 127.9 (2C), 127.8, 127.7, 127.4 (2C), 126.4, 125.2, 123.4, 121.7, 116.8, 114.7, 111.0, 48.9. IR (mineral oil), cm^{-1}: 3269, 1595, 1575, 1558, 1533, 1487, 1457. Anal. Calcd for $C_{26}H_{20}N_2S$: C 79.56; H 5.14; N 7.14; S 8.17. Found: C 79.99; H 5.31; N 7.02 S 8.32.

4-(5-(Benzo[*d*]thiazol-2-yl)-[1,1'-biphenyl]-3-yl)morpholine (3cb)

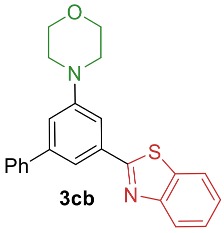

3cb

According to the **GP A**; from 1,3-diketone **1c** (0.5 mmol), amine **2b** (0.75 mmol), acetone (1 mL) and $CHCl_3$ (1 mL); reaction time 4 d, recrystallized from EtOH. Beige powder, 108 mg (58%), mp 165–167 °C, R_f = 0.50 (PhMe/EtOAc 5:1).

^{1}H NMR (400 MHz, $CDCl_3$) δ 8.10 (d, J = 8.2 Hz, 1H), 7.92 (dd, J = 8.0, 1.2 Hz, 1H), 7.82 – 7.76 (m, 1H), 7.72 (t, J = 1.9 Hz, 1H), 7.69 – 7.63 (m, 2H), 7.56 – 7.44 (m, 3H), 7.40 (td, J

= 7.6, 1.3 Hz, 2H), 7.26 (s, 1H), 4.05 – 3.87 (m, 4H), 3.44 – 3.26 (m, 4H). **^{13}C NMR** (101 MHz, $CDCl_3$) δ 168.5, 154.3, 152.1, 143.5, 141.0, 135.3, 135.1, 129.0 (2C), 127.9, 127.5 (2C), 126.5, 125.4, 123.4, 121.7, 118.9, 117.4, 113.4, 67.0 (2C), 49.6 (2C). IR (mineral oil), cm^{-1}: 1591, 1574, 1508, 1493, 1461. Anal. Calcd for $C_{23}H_{20}N_2OS$: C 74.16; H 5.41; N 7.52; S 8.61. Found: C 74.44; H 5.45; N 7.54 S 8.49.

_N_-Benzyl-5-(5-phenyloxazol-2-yl)-[1,1'-biphenyl]-3-amine (3da)

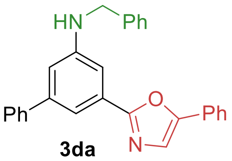

3da

According to the **GP B**; from 1,3-diketone **1d** (0.5 mmol), amine **2a** (0.75 mmol), AcOH (30 mol %), molecular sieves 3 Å (300 mg) and acetone (2 mL); reaction time 4 d, recrystallized from EtOH. Beige-yellow powder, 90 mg (45%), mp 163–165 °C, R_f = 0.57 (PhMe/EtOAc 5:1).

^{1}H NMR (400 MHz, $CDCl_3$) δ 7.80 – 7.67 (m, 3H), 7.63 (d, *J* = 7.0 Hz, 2H), 7.50 – 7.28 (m, 13H), 6.95 (t, *J* = 1.9 Hz, 1H), 4.48 (s, 2H), 4.45 (br. s, 1H). **^{13}C NMR** (101 MHz, $CDCl_3$) δ 161.7, 151.3, 149.0, 143.2, 141.1, 139.2, 129.0 (2C), 128.9 (3C), 128.8 (2C), 128.5, 128.3, 127.8 (2C), 127.7, 127.6, 127.4 (2C), 124.4 (2C), 123.6, 115.0, 113.9, 109.5, 48.6. IR (mineral oil), cm^{-1}: 3432, 1598, 1576, 1534, 1516, 1490, 1463. Anal. Calcd for $C_{28}H_{22}N_2O$: C 83.56; H 5.51; N 6.96. Found: C 83.69; H 5.68; N 7.05.

4-(5-(5-Phenyloxazol-2-yl)-[1,1'-biphenyl]-3-yl)morpholine (3db)

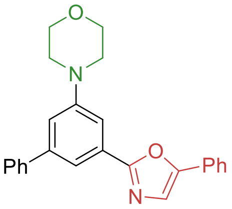

3db

According to the **GP A**; from 1,3-diketone **1d** (0.5 mmol), amine **2b** (0.75 mmol), acetone (1 mL), $CHCl_3$ (1 mL) and 1.5 equiv of AcOH (45 uL); reaction time 4 d, recrystallized from EtOH. Beige-yellow powder, 78 mg (41%), mp 165–167 °C, R_f = 0.35 (PhMe/EtOAc 5:1, 1% AcOH).

^{1}H NMR (400 MHz, $CDCl_3$) δ 7.84 (t, *J* = 1.5 Hz, 1H), 7.78 – 7.72 (m, 2H), 7.68 – 7.63 (m, 3H), 7.52 – 7.42 (m, 5H), 7.42 – 7.32 (m, 2H), 7.21 (dd, *J* = 2.5, 1.5 Hz, 1H), 4.01 – 3.86 (m, 4H), 3.43 – 3.24 (m, 4H). **^{13}C NMR** (101 MHz, $CDCl_3$) δ 161.6, 152.2, 151.5, 143.3, 141.2, 129.1 (2C), 128.93 (2C), 128.88, 128.6, 128.2, 127.9, 127.5 (2C), 124.5 (2C), 123.6, 117.1, 116.7, 112.3, 67.0 (2C), 49.5 (2C). IR (mineral oil), cm^{-1}: 1594, 1572, 1532, 1497, 1487, 1460. Anal. Calcd for $C_{25}H_{22}N_2O_2$: C 78.51; H 5.80; N 7.32. Found: C 78.70; H 5.97; N 7.46.

_N_-Benzyl-5-(5-phenylisoxazol-3-yl)-[1,1'-biphenyl]-3-amine (3ea)

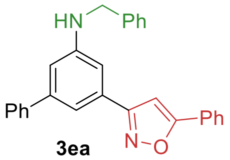

3ea

According to the **GP A**; from 1,3-diketone **1e** (0.3 mmol), amine **2a** (0.45 mmol), acetone (1 mL) and molecular sieves 3 Å (300 mg); reaction time 6d; purified via flash column chromatography (PhMe/hexane 6:1). Off-white powder, 26 mg (21%), mp 114–116 °C, R_f = 0.34 (PhMe/hexane 5:1).

^{1}H NMR (400 MHz, $CDCl_3$) δ 7.92 – 7.80 (m, 2H), 7.65 – 7.56 (m, 2H), 7.53 – 7.34 (m, 11H), 7.34 – 7.28 (m, 1H), 7.20 (t, *J* = 1.9 Hz, 1H), 6.94 (t, *J* = 1.9 Hz, 1H), 6.82 (s, 1H), 4.46 (s, 2H), 4.28 (s, 1H). **^{13}C NMR** (101 MHz, $CDCl_3$) δ 170.4, 163.5, 149.1, 143.3, 141.3, 139.2, 130.6, 130.3, 129.1 (2C), 128.9 (2C), 128.8 (2C), 127.8, 127.78, 127.75 (2C), 127.6, 127.4 (2C), 126.0 (2C), 115.7, 113.5, 110.0, 97.9, 48.6. IR (mineral oil), cm^{-1}: 3338, 1606, 1573, 1542, 1494, 1459. Anal. Calcd for $C_{28}H_{22}N_2O$: C 83.56; H 5.51; N 6.96. Found: C 83.98; H 5.62; N 7.21.

_N_-Benzyl-5-(furan-2-yl)-[1,1'-biphenyl]-3-amine (3fa)

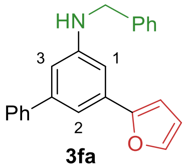

3fa

According to the **GP A**; from 1,3-diketone **1f** (0.5 mmol), amine **2j** (2.5 mmol), AcOH (30 mol %) and acetone (2 mL); reaction time 7 d, isolated via flash column chromatography (PhMe/hexane 1:2). Yellow oil, 29 mg (18%), R_f = 0.45 (PhMe/hexane 1:1). Note: this product was not isolated in pure form due to the presence of many side-products that complicate isolation. Crude yield is provided. The presence of the protons $H^1/H^2/H^3$ (dd or t due to the characteristic W-coupling) of the central benzene ring confirms the formation of the product **3fa**.

^{1}H NMR (400 MHz, $CDCl_3$) δ 7.64 – 7.58 (m, 2H), 7.48 – 7.27 (m, 10H), 7.07 – 6.95 (m, 1H), 6.78 (t, *J* = 1.9 Hz, 1H), 6.65 (d, *J* = 3.3 Hz, 1H), 6.48 (dd, *J* = 3.3, 1.8 Hz, 1H), 4.44 (s, 2H), 3.92 (s, 1H). **^{13}C NMR** (101 MHz, $CDCl_3$) δ 154.5, 149.0, 143.0, 142.0, 141.7, 139.4, 132.4, 128.84 (2C), 128.76 (2C), 127.8 (2C), 127.5, 127.5, 127.3 (2C), 113.0, 111.7, 111.2, 107.4, 105.3, 48.6.

_N_-Benzyl-4'-methoxy-5-(3-phenyl-1,2,4-oxadiazol-5-yl)-[1,1'-biphenyl]-3-amine (3ha)

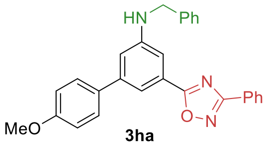

3ha

According to the **GP A**; from 1,3-diketone **1h** (0.5 mmol), amine **2a** (0.55 mmol) and acetone (2 mL); reaction time 1 d, recrystallized from EtOH. Off-white powder, 167 mg (77%), mp 149–150 °C, R_f = 0.85 (PhMe/EtOAc 5:1).

^{1}H NMR (400 MHz, $CDCl_3$) δ 8.26 – 8.16 (m, 2H), 7.77 (s, 1H), 7.63 – 7.48 (m, 5H), 7.46 – 7.35 (m, 5H), 7.31 (t, *J* = 7.1 Hz, 1H), 7.06 – 6.93 (m, 3H), 4.46 (d, *J* = 5.3 Hz, 2H), 4.34 (t, *J* = 5.3 Hz, 1H), 3.86 (s, 3H). **^{13}C NMR** (101 MHz, $CDCl_3$) δ 176.4, 169.1, 159.8, 149.1, 143.1, 138.9, 133.1, 131.2, 128.9 (4C), 128.4 (2C), 127.8 (2C), 127.72 (2C), 127.68, 127.4, 125.7, 116.2, 115.4, 114.5 (2C), 110.3, 55.5, 48.5. IR (mineral oil), cm^{-1}: 3447, 1610, 1582, 1560, 1521, 1505. Anal. Calcd for $C_{28}H_{23}N_3O_2$: C 77.58; H 5.35; N 9.69. Found: C 77.76; H 5.24; N 9.75.

_N_-Benzyl-4'-nitro-5-(3-phenyl-1,2,4-oxadiazol-5-yl)-[1,1'-biphenyl]-3-amine (3ia)

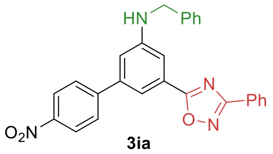

3ia

According to the **GP A**; from 1,3-diketone **1i** (0.5 mmol), amine **2a** (0.55 mmol) and acetone (2 mL); reaction time 1 d, recrystallized from EtOH. Light-orange powder, 161 mg (72%), mp 189–191 °C, R_f = 0.84 (PhMe/EtOAc 5:1).

^{1}H NMR (400 MHz, DMSO-*d*$_6$) δ 8.25 (d, *J* = 8.9 Hz, 2H), 8.12 – 8.00 (m, 2H), 7.87 (d, *J* = 8.9 Hz, 2H), 7.65 – 7.51 (m, 4H), 7.44 (dd, *J* = 2.3, 1.4 Hz, 1H), 7.42 – 7.36 (m, 2H), 7.30 (t, *J* = 7.6 Hz, 2H), 7.22 – 7.16 (m, 2H), 6.90 (t, *J* = 5.8 Hz, 1H), 4.42 (d, *J* = 5.8 Hz, 2H). **^{13}C NMR** (101 MHz, DMSO-*d*$_6$) δ 175.6, 168.1, 149.9, 146.9, 146.0, 139.8, 139.2, 131.5, 129.1 (2C), 128.3 (2C), 127.8 (2C), 127.2 (2C), 127.0 (2C), 126.8, 126.1, 124.7 (2C), 124.0, 114.8, 113.6, 111.5, 46.2. IR (mineral oil), cm^{-1}: 3418, 1597, 1557, 1512. Anal. Calcd for $C_{27}H_{20}N_4O_3$: C 72.31; H 4.50; N 12.49. Found: C 72.65; H 4.41; N 12.55.

2-((5-(3-Phenyl-1,2,4-oxadiazol-5-yl)-[1,1'-biphenyl]-3-yl)amino)phenol (3ad)

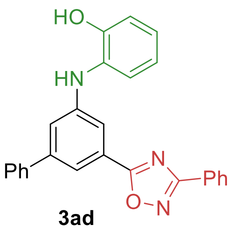

3ad

According to the **GP B**; from 1,3-diketone **1a** (0.5 mmol), amine **2d** (0.75 mmol) and acetone (2 mL); reaction time 1 d; recrystallized from EtOH. Beige powder, 143 mg (70%), mp 203–205 °C. R_f = 0.55 (PhMe/EtOAc 5:1).

^{1}H NMR (400 MHz, DMSO-*d*$_6$) δ 9.52 (s, 1H), 8.15 – 8.03 (m, 2H), 7.88 (s, 1H), 7.72 (t, *J* = 1.6 Hz, 1H), 7.70 – 7.64 (m, 3H), 7.63 – 7.56 (m, 3H), 7.51 (t, *J* = 7.6 Hz, 2H), 7.45 – 7.39 (m, 2H), 7.28 (d, *J* = 7.2 Hz, 1H), 6.99 – 6.91 (m, 2H), 6.88 – 6.80 (m, 1H). **^{13}C NMR** (101 MHz, DMSO-*d*$_6$) δ 175.7, 168.1, 149.9, 146.8, 142.1, 139.5, 131.5, 129.1 (2C), 128.9 (2C),

128.8, 127.9, 127.0 (2C), 126.6 (2C), 126.1, 124.4, 123.8, 122.1, 119.3, 117.5, 116.0, 115.3, 112.2. IR (mineral oil), cm^{-1}: 3479, 3327, 1598, 1557, 1494, 1460. Anal. Calcd for $C_{26}H_{19}N_3O_2$: C 77.02; H 4.72; N 10.36. Found: C 77.17; H 4.87; N 10.44.

2-((5-(3-Phenyl-1,2,4-oxadiazol-5-yl)-[1,1'-biphenyl]-3-yl)amino)ethan-1-ol (3ae)

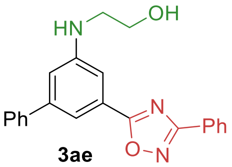

3ae

According to the **GP A**; from 1,3-diketone **1a** (0.5 mmol), amine **2e** (0.55 mmol) and acetone (2 mL); reaction time 1 d; recrystallized from Et_2O/hexane. Off-white powder, 136 mg (76%), mp 125–128 °C, R_f = 0.17 (PhMe/EtOAc 5:1).

^{1}H NMR (400 MHz, $CDCl_3$) δ 8.24 – 8.11 (m, 2H), 7.81 (d, *J* = 1.5 Hz, 1H), 7.62 (d, *J* = 7.1 Hz, 2H), 7.53 – 7.42 (m, 6H), 7.38 (t, *J* = 7.3 Hz, 1H), 7.11 (t, *J* = 1.9 Hz, 1H), 3.94 (t, *J* = 5.2 Hz, 2H), 3.46 (t, *J* = 5.2 Hz, 2H), 3.30 (s, 2H). **^{13}C NMR** (101 MHz, $CDCl_3$) δ 176.2, 169.1, 148.6, 143.6, 140.4, 131.3, 128.99 (2C), 128.96 (2C), 128.1, 127.7 (2C), 127.3 (2C), 127.2, 125.8, 117.3, 116.8, 111.4, 61.2, 46.7. IR (mineral oil), cm^{-1}: 3382, 3247, 1599, 1579, 1558, 1526, 1461. Anal. Calcd for $C_{22}H_{19}N_3O_2$: C 73.93; H 5.36; N 11.76. Found: C 74.18; H 5.44; N 11.95.

***N*-(5-(3-Phenyl-1,2,4-oxadiazol-5-yl)-[1,1'-biphenyl]-3-yl)pyridin-3-amine (3af)**

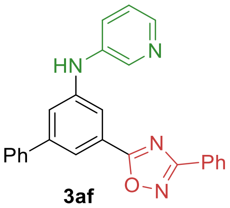

3af

According to the **GP B**; from 1,3-diketone **1a** (0.5 mmol), amine **2f** (0.75 mmol), AcOH (30 mol%), molecular sieves 3 Å (300 mg) and acetone (2 mL); reaction time 6 d; purified by flash column chromatography (PhMe/EA 3:1). Beige powder, 117 mg (60%), mp 172–175 °C. R_f = 0.13 (PhMe/EtOAc 3:1).

^{1}H NMR (400 MHz, $CDCl_3$) δ 8.51 (d, *J* = 2.7 Hz, 1H), 8.34 – 8.23 (m, 1H), 8.20 – 8.11 (m, 2H), 8.01 (d, *J* = 1.5 Hz, 1H), 7.83 (t, *J* = 1.8 Hz, 1H), 7.64 – 7.57 (m, 2H), 7.55 – 7.43 (m, 7H), 7.39 (t, *J* = 7.3 Hz, 1H), 7.25 (dd, *J* = 8.1, 4.8 Hz, 1H), 6.20 (s, 1H). **^{13}C NMR** (101 MHz, $CDCl_3$) δ 175.6, 169.2, 144.0, 143.9, 143.4, 141.4, 139.8, 139.0, 131.3, 129.1 (2C), 129.0 (2C), 128.3, 127.7 (2C), 127.3 (2C), 127.1, 126.2, 125.2, 124.1, 120.2, 120.0, 115.5. IR (mineral oil), cm^{-1}: 3233, 1613, 1584, 1562, 1497, 1465. Anal. Calcd for $C_{25}H_{18}N_4O$: C 76.91; H 4.65; N 14.35. Found: C 77.15; H 4.77; N 14.23.

5-(3-Phenyl-1,2,4-oxadiazol-5-yl)-*N*-(4-(trifluoromethyl)phenyl)-[1,1'-biphenyl]-3-amine (3ag)

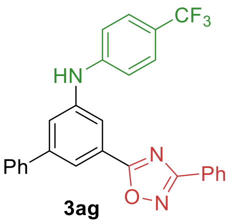

3ag

According to the **GP B**; from 1,3-diketone **1a** (0.3 mmol), amine **2g** (0.45 mmol), AcOH (30 mol%), molecular sieves 3Å (300 mg) and acetone (1 mL); reaction time 8d, flash column chromatography (hexane/EtOAc 10:1; loading in DCM). Light-yellow powder, 45 mg (33%), mp 185–187 °C. R_f = 0.78 (PhMe/EtOAc 5:1).

^{1}H NMR (400 MHz, $CDCl_3$) δ 8.23 – 8.15 (m, 2H), 8.08 (s, 1H), 7.92 (s, 1H), 7.64 (d, *J* = 7.1 Hz, 2H), 7.59 – 7.45 (m, 8H), 7.42 (t, *J* = 7.3 Hz, 1H), 7.18 (d, *J* = 8.3 Hz, 2H), 6.16 (s, 1H). **^{13}C NMR** (101 MHz, $CDCl_3$) δ 175.6, 169.3, 145.8, 144.0, 143.0, 139.6, 131.4, 129.2 (2C), 129.0 (2C), 128.4, 127.7 (2C), 127.3 (2C), 127.1 (q, *J* = 3.9 Hz), 127.1, 126.2, 124.6 (q, *J* = 270.9 Hz), 123.3 (2C, q, *J* = 32.8 Hz), 121.7, 121.0, 117.1, 116.7 (2C). ^{19}F NMR (377 MHz, $CDCl_3$) δ -64.75. IR (mineral oil), cm^{-1}: 3318, 1594, 1560, 1523, 1501, 1461. Anal. Calcd for $C_{27}H_{18}F_3N_3O$: C 70.89; H 3.97; N 9.19. Found: C 71.17; H 4.16; N 9.37.

N-Allyl-5-(5-phenyl-1,3,4-oxadiazol-2-yl)-[1,1'-biphenyl]-3-amine (3ai)

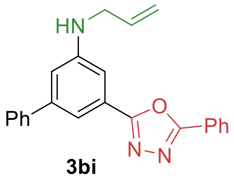

3bi

According to the **GP A**; from 1,3-diketone **1b** (0.5 mmol), amine **2i** (0.75 mmol), acetone (2 mL) and $CHCl_3$ (1 mL); reaction time 1d, recrystallized from EtOH. Light-yellow powder, 124 mg (70%), mp 161–163 °C, R_f = 0.46 (PhMe/EtOAc 5:1).

^{1}H NMR (400 MHz, $CDCl_3$) δ 8.20 – 8.10 (m, 2H), 7.72 – 7.67 (m, 1H), 7.67 – 7.62 (m, 2H), 7.59 – 7.50 (m, 3H), 7.50 – 7.43 (m, 2H), 7.43 – 7.34 (m, 2H), 7.02 (dd, *J* = 2.3, 1.6 Hz, 1H), 6.02 (ddt, *J* = 17.2, 10.5, 5.3 Hz, 1H), 5.37 (dd, *J* = 17.2, 1.6 Hz, 1H), 5.24 (dd, *J* = 10.3, 1.5 Hz, 1H), 4.54 (s, 1H), 3.93 (dt, *J* = 5.4, 1.6 Hz, 2H). **^{13}C NMR** (101 MHz, $CDCl_3$) δ 165.2, 164.7, 148.7, 143.5, 140.7, 134.8, 131.8, 129.2 (2C), 129.0 (2C), 128.0, 127.3 (2C), 127.1 (2C), 125.3, 124.3, 117.1, 115.5, 115.4, 110.2, 46.8. IR (mineral oil), cm^{-1}: 3333, 1604, 1550, 1529, 1463. Anal. Calcd for $C_{23}H_{19}N_3O$: C 78.16; H 5.42; N 11.89. Found: C 78.56; H 5.51; N 12.02.

N-(2,2-Dimethoxyethyl)-5-(5-phenyl-1,3,4-oxadiazol-2-yl)-[1,1'-biphenyl]-3-amine (3aj)

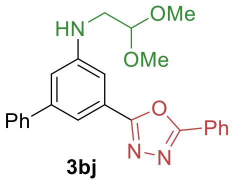

3bj

According to the **GP A**; from 1,3-diketone **1b** (0.5 mmol), amine **2j** (0.75 mmol), acetone (2 mL) and $CHCl_3$ (1 mL); reaction time 12 h, recrystallized from EtOH. Off-white powder, 162 mg (80%), mp 169–171 °C. R_f = 0.14 (PhMe/EtOAc 5:1).

^{1}H NMR (400 MHz, $CDCl_3$) δ 8.24 – 8.09 (m, 2H), 7.69 (t, *J* = 1.5 Hz, 1H), 7.66 – 7.61 (m, 2H), 7.53 (qd, *J* = 4.7, 1.5 Hz, 3H), 7.46 (t, *J* = 7.3 Hz, 2H), 7.41 – 7.33 (m, 2H), 7.07 – 6.93 (m, 1H), 4.63 (t, *J* = 5.4 Hz, 1H), 4.23 (s, 1H), 3.46 (s, 6H), 3.41 (d, *J* = 5.4 Hz, 2H).**^{13}C NMR** (101 MHz, $CDCl_3$) δ 165.1, 164.7, 148.9, 143.5, 140.7, 131.7, 129.2 (2C), 128.9 (2C), 127.9, 127.3 (2C), 127.1 (2C), 125.4, 124.3, 115.4, 115.3, 109.8, 102.7, 54.1 (2C), 45.6. IR (mineral oil), cm^{-1}: 3402, 1598, 1576, 1540, 1513, 1460. Anal. Calcd for $C_{24}H_{23}N_3O_3$: C 71.80; H 5.77; N 10.47. Found: C 72.17; H 5.83; N 10.31.

N-(2,6-Diisopropylphenyl)-5-(3-phenyl-1,2,4-oxadiazol-5-yl)-[1,1'-biphenyl]-3-amine (3ah)

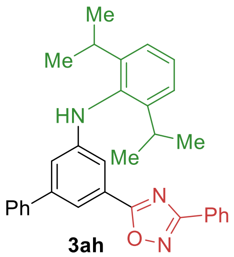

3ah

According to the **GP B**; from 1,3-diketone **1a** (0.3 mmol), amine **2h** (0.45 mmol), AcOH (30 mol %), molecular sieves 3 Å (200 mg) and acetone (1 mL); reaction time 6 d, recrystallized from MTBE/Me_2CO. Beige powder, 75 mg (53%), mp 189–190 °C. R_f = 0.39 (PhMe/Hexane 1:2).

^{1}H NMR (400 MHz, $CDCl_3$) δ 8.25 – 8.12 (m, 2H), 7.82 (t, *J* = 1.5 Hz, 1H), 7.64 – 7.55 (m, 2H), 7.51 (qd, *J* = 4.3, 1.5 Hz, 3H), 7.44 (t, *J* = 7.4 Hz, 2H), 7.40 – 7.32 (m, 3H), 7.31 – 7.23 (m, 2H), 6.87 (s, 1H), 5.46 (s, 1H), 3.27 (hept, *J* = 6.9 Hz, 2H), 1.21 (d, *J* = 6.9 Hz, 12H). **^{13}C NMR** (101 MHz, $CDCl_3$) δ 176.3, 169.1, 149.3, 147.7 (2C), 143.6, 140.5, 134.3, 131.2, 129.0 (2C), 128.9 (2C), 128.1, 128.0, 127.7 (2C), 127.33, 127.31 (2C), 125.8, 124.3 (2C), 116.8, 115.5, 111.3, 28.6 (2C), 24.0 (4C). IR (mineral oil), cm^{-1}: 3322, 1614, 1583, 1562, 1460. Anal. Calcd for $C_{32}H_{31}N_3O$: C 81.15; H 6.60; N 8.87. Found: C 81.27; H 6.83; N 8.94.

3-(3-Fluoro-4-morpholinophenyl)-5-(((5-(3-phenyl-1,2,4-oxadiazol-5-yl)-[1,1'-biphenyl]-3-yl)amino)methyl)oxazolidin-2-one (3ak)

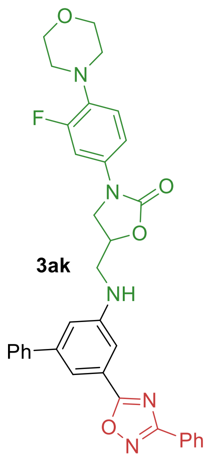

3ak

According to the **GP A**; from 1,3-diketone **1a** (0.2 mmol), amine **2k** (0.2 mmol) and acetone (1 mL); reaction time 19 h, recrystallized from EtOH. Off-white powder, 100 mg (85%), mp 207–210 °C, R_f = 0.15 (PhMe/EtOAc 5:1).

^{1}H NMR (400 MHz, DMSO-*d6*) δ 8.17 – 8.05 (m, 2H), 7.78 – 7.67 (m, 2H), 7.65 – 7.55 (m, 4H), 7.55 – 7.47 (m, 4H), 7.47 – 7.38 (m, 1H), 7.28 (t, *J* = 2.0 Hz, 1H), 7.24 – 7.16 (m, 1H), 7.04 (t, *J* = 9.3 Hz, 1H), 6.56 (t, *J* = 6.1 Hz, 1H), 4.94 (dtd, *J* = 9.0, 6.2, 4.3 Hz, 1H), 4.18 (t, *J* = 8.9 Hz, 1H), 3.86 (dd, *J* = 9.1, 6.4 Hz, 1H), 3.78 – 3.70 (m, 4H), 3.69 – 3.54 (m, 2H), 3.01 – 2.89 (m, 4H). **^{13}C NMR** (101 MHz, DMSO-*d6*) δ 175.9, 168.1, 154.5 (d, *J* = 243.6 Hz), 154.0, 149.7, 142.3, 139.6, 135.4 (d, *J* = 8.8 Hz), 133.4 (d, *J* = 10.7 Hz), 131.5, 129.1 (2C), 128.8 (2C), 127.8, 127.0 (2C), 126.7 (2C), 126.2, 124.4, 119.1 (d, *J* = 4.2 Hz), 114.6, 114.0 (d, *J* = 3.0 Hz), 113.9, 110.3, 106.6 (d, *J* = 26.1 Hz), 71.6, 66.0 (2C), 50.6 (d, *J* = 3.0 Hz, 2C), 47.4, 45.6. **^{19}F NMR** (377 MHz, DMSO-*d6*) δ -123.59. IR (mineral oil), cm^{-1}: 3416, 1732, 1607, 1559, 1517, 1460. Anal. Calcd for $C_{34}H_{30}FN_5O_4$: C 69.02; H 5.11; N 11.84. Found: C 69.36; H 5.21; N 11.67.

Crystal structure determination

The unit cell parameters and the X-ray diffraction intensities were measured on a Xcalibur Ruby diffractometer. The empirical absorption correction was introduced by multi-scan method using SCALE3 ABSPACK algorithm [9]. Using the Olex2 [10], the structures were solved with the SHELXS [11] or SHELXD [11] programs and refined by the full-matrix least-squares method in the anisotropic approximation for all non-hydrogen atoms with the SHELXL program [12]. Hydrogen atoms bound to carbon were positioned geometrically and refined using a riding model. The hydrogen atoms of NH and OH groups were refined independently with isotropic displacement parameters.

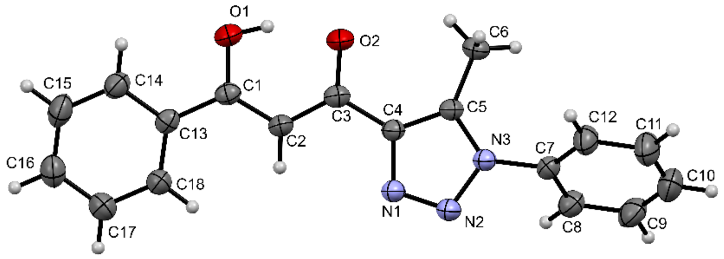

Figure S1. *Molecular structure of compound* ***1g*** *showing 30% probability amplitude displacement ellipsoids.*

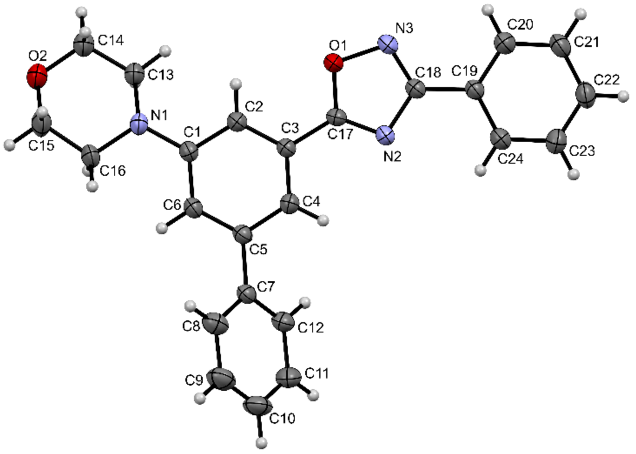

Figure S2. *Molecular structure of compound* ***3ab*** *showing 30% probability amplitude displacement ellipsoids.*

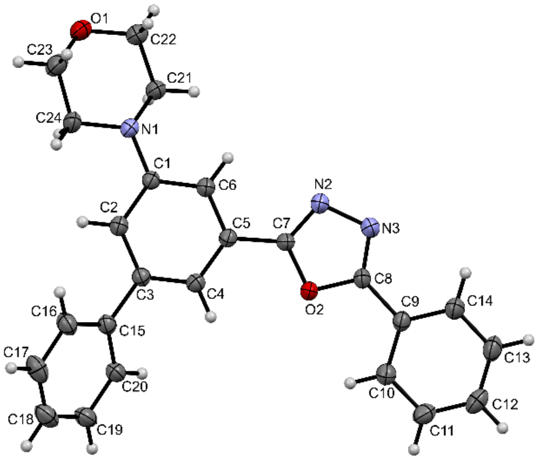

Figure S3. *Molecular structure of compound* ***3bb*** *showing 30% probability amplitude displacement ellipsoids.*

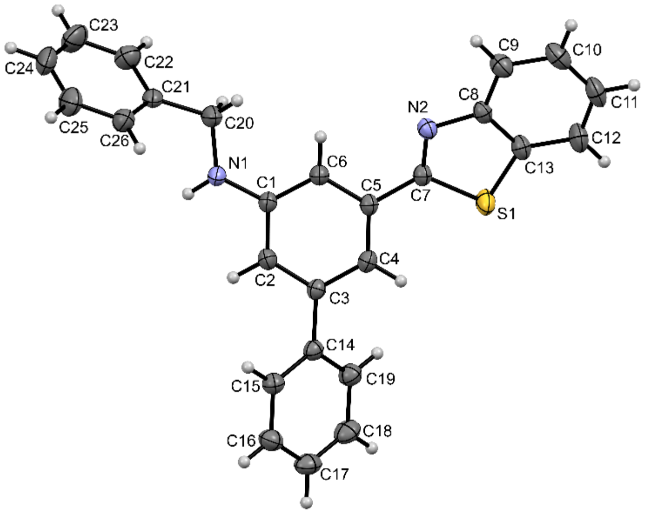

Figure S4. *Molecular structure of compound* ***3ca*** *showing 30% probability amplitude displacement ellipsoids.*

Table S1. Crystal data and structure refinement for compounds **1g**, **3ab**, **3bb, 3ca**.

Compound	**1g**	**3ab**	**3bb**	**3ca**
**CCDC**	2356152	2356151	2356154	2356153
Empirical formula	$C_{18}H_{15}N_3O_2$	$C_{24}H_{21}N_3O_2$	$C_{24}H_{21}N_3O_2$	$C_{26}H_{20}N_2S$
Formula weight	305.33	383.44	383.44	392.50
Temperature, K	295(2)	295(2)	295(2)	295(2)
Crystal system	monoclinic	monoclinic	monoclinic	orthorhombic
Space group	$P2_1/c$	$P2_1/n$	$P2_1/n$	Pbca
a, Å	6.513(2)	6.6892(16)	6.6905(12)	8.146(2)
b, Å	8.256(3)	10.681(3)	10.677(2)	19.701(5)
c, Å	29.053(10)	27.077(9)	27.214(6)	25.178(5)
α, °	90	90	90	90
β, °	92.13(3)	89.85(2)	90.41(2)	90
γ, °	90	90	90	90
Volume, $Å^3$	1561.0(10)	1934.5(10)	1944.0(7)	4040.7(17)
Z	4	4	4	8
Density (calculated), g/cm^3	1.299	1.317	1.310	1.290

Absorption coefficient, mm^{-1}	0.087	0.085	0.085	0.175
F(000)	640.0	808.0	808.0	1648.0
Crystal size, mm^3	0.6 × 0.3 × 0.08	0.6 × 0.1 × 0.05	0.6 × 0.4 × 0.1	0.55 × 0.25 × 0.2
Radiation	Mo Kα (λ = 0.71073)	Mo Kα (λ = 0.71073)	Mo Kα (λ = 0.71073)	Mo Kα (λ = 0.71073)
2Θ range for data collection, °	5.612 to 59.062	4.858 to 59.3	4.49 to 59.38	5.648 to 58.772
Index ranges	-9 ≤ h ≤ 6, -7 ≤ k ≤ 11, -39 ≤ l ≤ 30	-8 ≤ h ≤ 9, -9 ≤ k ≤ 14, -35 ≤ l ≤ 37	-7 ≤ h ≤ 9, -13 ≤ k ≤ 8, -37 ≤ l ≤ 24	-11 ≤ h ≤ 10, -26 ≤ k ≤ 23, -23 ≤ l ≤ 34
Reflections collected	7464	9348	7421	14590
Independent reflections	3687 [R_{int} = 0.0396, R_{sigma} = 0.0632]	4536 [R_{int} = 0.0545, R_{sigma} = 0.0928]	4261 [R_{int} = 0.0508, R_{sigma} = 0.0682]	4874 [R_{int} = 0.0401, R_{sigma} = 0.0494]
Data/restraints/parameters	3687/1/214	4536/0/262	4261/0/264	4874/0/267
Goodness-of-fit on F^2	1.014	1.032	1.095	1.029
Final R indexes [I>=2σ (I)]	R_1 = 0.0571, wR_2 = 0.1351	R_1 = 0.0717, wR_2 = 0.1403	R_1 = 0.0556, wR_2 = 0.1198	R_1 = 0.0513, wR_2 = 0.1152
Final R indexes [all data]	R_1 = 0.1173, wR_2 = 0.1824	R_1 = 0.1670, wR_2 = 0.1963	R_1 = 0.0816, wR_2 = 0.1518	R_1 = 0.0923, wR_2 = 0.1411
Largest diff. peak/hole, $eÅ^{-3}$	0.17/-0.20	0.17/-0.26	0.21/-0.19	0.16/-0.28

Computational details

The DFT calculations for all model structures (**Figure S5**) were carried out at the M06-2X/6-31G* level of theory with the help of the Gaussian-09 program package [13]. No symmetry restrictions have been applied during the geometry optimization procedure. The Hessian matrices were calculated analytically for all optimized model structures to prove the location of correct minima on the potential energy surfaces. The Cartesian atomic coordinates for all model structures are presented in attached xyz-files (see Supporting Information File 2).

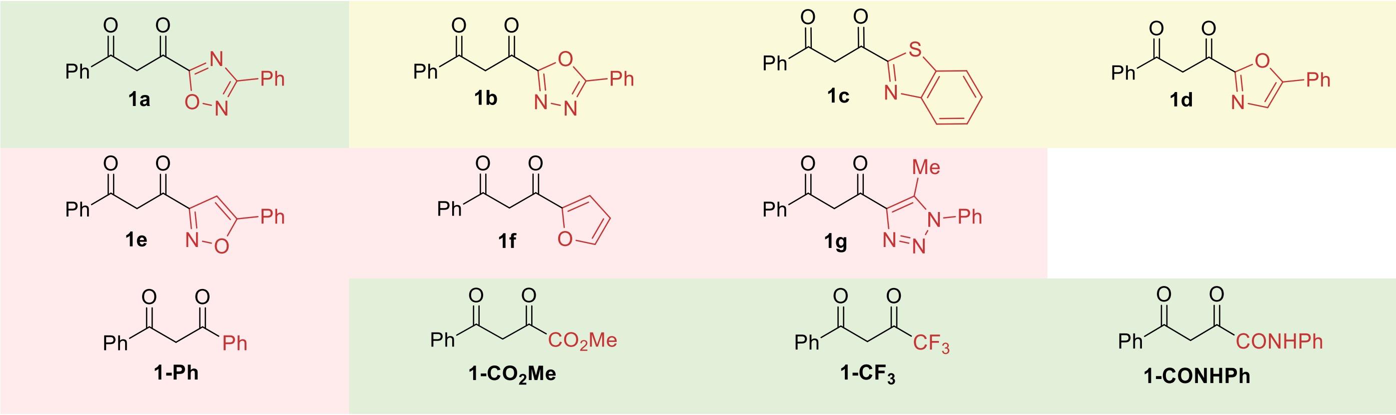

Figure S5. The model series of 1,3-diketones (green – successful reaction with alkyl- and arylamines; yellow – successful reaction with alkylamines; red – unsuccessful or complicated reaction with alkylamines). 1,3-Diketones 1-Ph, 1-CO_2Me, 1-CF_3 and 1-CONHPh were studied previously [14].

Mulliken and Gasteiger (PEOE) charges were calculated for the 1,3-diketones in their keto and enol forms. Unfortunately, there is no correlation of reactivity with the Mulliken charges on the carbonyl carbon. A slightly better trend in reactivity is seen in the Gasteiger charges of the carbonyl carbon (**Table S2**). However, the Hammett constants calculated using Ertl's web-based tool [15] provide the best prediction of reactivity for these 1,3-diketones (**Figure S6**).

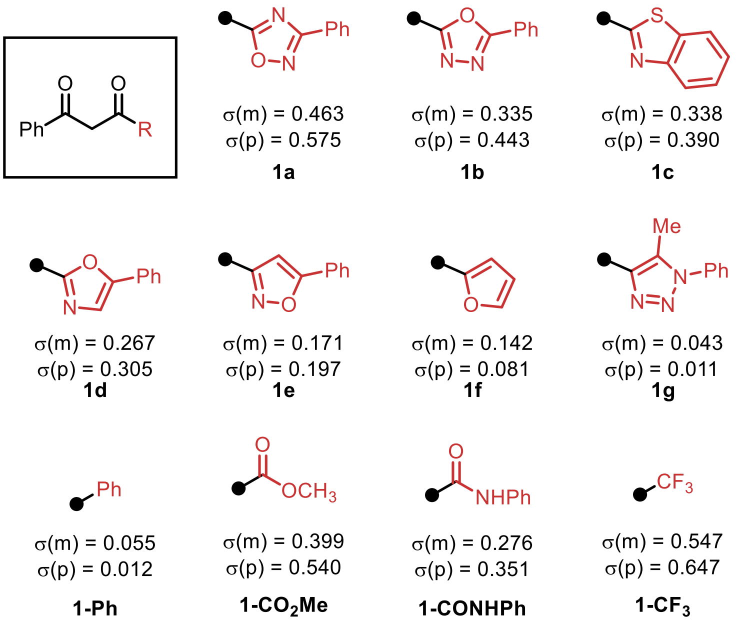

Figure S6. The model series of 1,3-diketones with calculated (Ertl's web-based tool) Hammett constants.

Table S2. The calculated charges for the 1,3-diketone model series

1,3-diketones	Charges of the enol form				Charges of the keto form			
	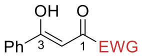				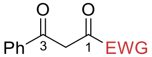			
	C^1-EWG		C^3-Ph		C^1-EWG		C^3-Ph	
	Gasteiger (PEOE)	Mulliken	Gasteiger (PEOE)	Mulliken	Gasteiger (PEOE)	Mulliken	Gasteiger (PEOE)	Mulliken
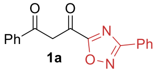 1a	0.23281078	0.397155	0.08253728	0.369021	0.21489088	0.401371	0.17026388	0.436228
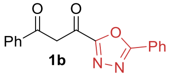 1b	0.23245502	0.407540	0.08253703	0.356896	0.21453425	0.447565	0.17026362	0.421719
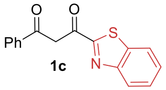 1c	0.21317288	0.429893	0.08247343	0.363714	0.19528561	0.464127	0.17019723	0.429492
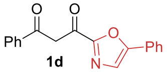 1d	0.23060343	0.394174	0.08253587	0.353392	0.21268238	0.436739	0.17026239	0.430439
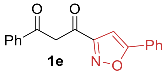 1e	0.20875028	0.426754	0.08245387	0.364520	0.19087076	0.422219	0.17017682	0.429744
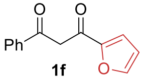 1f	0.21135056	0.404507	0.08247177	0.356366	0.19346649	0.430176	0.17019547	0.425726
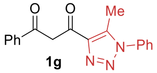 1g	0.20990789	0.434210	0.08245466	0.355750	0.19202673	0.426957	0.17017765	0.425768
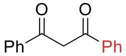 1-Ph	0.18795900	0.420700	0.08239133	0.363235	0.17011152	0.442541	0.17011152	0.408584
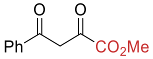 1-CO_2Me	0.25270809	0.339323	0.08263459	0.356169	0.23476923	0.412033	0.17033507	0.425854
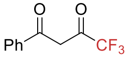 1-CF_3	0.26139441	0.290487	0.08240761	0.372031	0.24345676	0.364606	0.17010650	0.423194
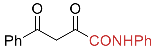 1-CONHPh	0.24320262	0.364377	0.08257455	0.364627	0.22526640	0.408754	0.17030279	0.429418

References:

1. Nekrasov D.D., Chizh V.G., Andreichikov Y.S., Tul'bovich G.A. *Zhurnal Organicheskoi Khimii*, *1996*, *32*, 761.
2. Krylova I.V., Nekrasov D.D., Andreichikov Y.S. *Khimiya Geterotsiklicheskikh Soedinenii*, **1988**, *11*, 1457.
3. Kumar N.N.B., Kuznetsov D.M., Kutateladze A.G. *Org. Lett.,* **2015**, 17, 438.
4. Stepanova E.E., Krasokha M.O., Galeev A.R., Dmitriev M.V., Maslivets A.N. *Russ J Org Chem,* **2018**, *54*, 1735.
5. Kalinin S., Valtari A., Ruponen M., Toropainen E., Kovalenko A., Nocentini A., Gureev M., Dar'in D., Urtti A., Supuran C.T., Krasavin M. *Bioorganic & Medicinal Chemistry,* **2019**, *27*, 115086.
6. Klein E., DeBonis, S., Thiede B., Skoufias D.A., Kozielski F., Lebeau L. *Bioorganic & Medicinal Chemistry*, **2007**, *15*, 6474.
7. Rao H.S.P., Muthanna N. *European Journal of Organic Chemistry*, **2015**, 1525.
8. Golobokova T.V., Proidakov A.G., Kizhnyaev V.N. *Russ J Org Chem*, **2020**, *56*, 446.
9. CrysAlisPro, Agilent Technologies, Version 1.171.37.33 (release 27-03-2014 CrysAlis171 .NET).
10. Dolomanov O.V., Bourhis L.J., Gildea R.J., Howard J.A.K., Puschmann H. *J. Appl. Cryst.*, **2009**, *42*, 339.
11. Sheldrick G.M. *Acta Crystallogr. Sect. A Found. Crystallogr.* **2008**, *64*, 112.
12. Sheldrick G.M. *Acta Crystallogr., Sect. C: Struct. Chem.*, **2015**, *71*, 3.
13. Gaussian 09, Revision C.01, M. J. Frisch, G. W. Trucks, H. B. Schlegel, G. E. Scuseria, M. A. Robb, J. R. Cheeseman, G. Scalmani, V. Barone, B. Mennucci, G. A. Petersson, H. Nakatsuji, M. Caricato, X. Li, H. P. Hratchian, A. F. Izmaylov, J. Bloino, G. Zheng, J. L. Sonnenberg, M. Hada, M. Ehara, K. Toyota, R. Fukuda, J. Hasegawa, M. Ishida, T. Nakajima, Y. Honda, O. Kitao, H. Nakai, T. Vreven, J. A. Montgomery, Jr., J. E. Peralta, F. Ogliaro, M. Bearpark, J. J. Heyd, E. Brothers, K. N. Kudin, V. N. Staroverov, T. Keith, R. Kobayashi, J. Normand, K. Raghavachari, A. Rendell, J. C. Burant, S. S. Iyengar, J. Tomasi, M. Cossi, N. Rega, J. M. Millam, M. Klene, J. E. Knox, J. B. Cross, V. Bakken, C. Adamo, J. Jaramillo, R. Gomperts, R. E. Stratmann, O. Yazyev, A. J. Austin, R. Cammi, C. Pomelli, J. W. Ochterski, R. L. Martin, K. Morokuma, V. G. Zakrzewski, G. A. Voth, P. Salvador, J. J. Dannenberg, S. Dapprich, A. D. Daniels, O. Farkas, J. B. Foresman, J. V. Ortiz, J. Cioslowski, and D. J. Fox, Gaussian, Inc., Wallingford CT, 2010.
14. Galeev, A. R.; Dmitriev, M. V.; Mokrushin, I. G.; Mashevskaya, I. V.; Maslivets, A. N.; Rubin, M. *Org. Biomol. Chem.* **2019**, *17*, 10030.
15. Ertl, P. *Chemistry Methods* **2022**, *2*, e202200041.

NMR spectra

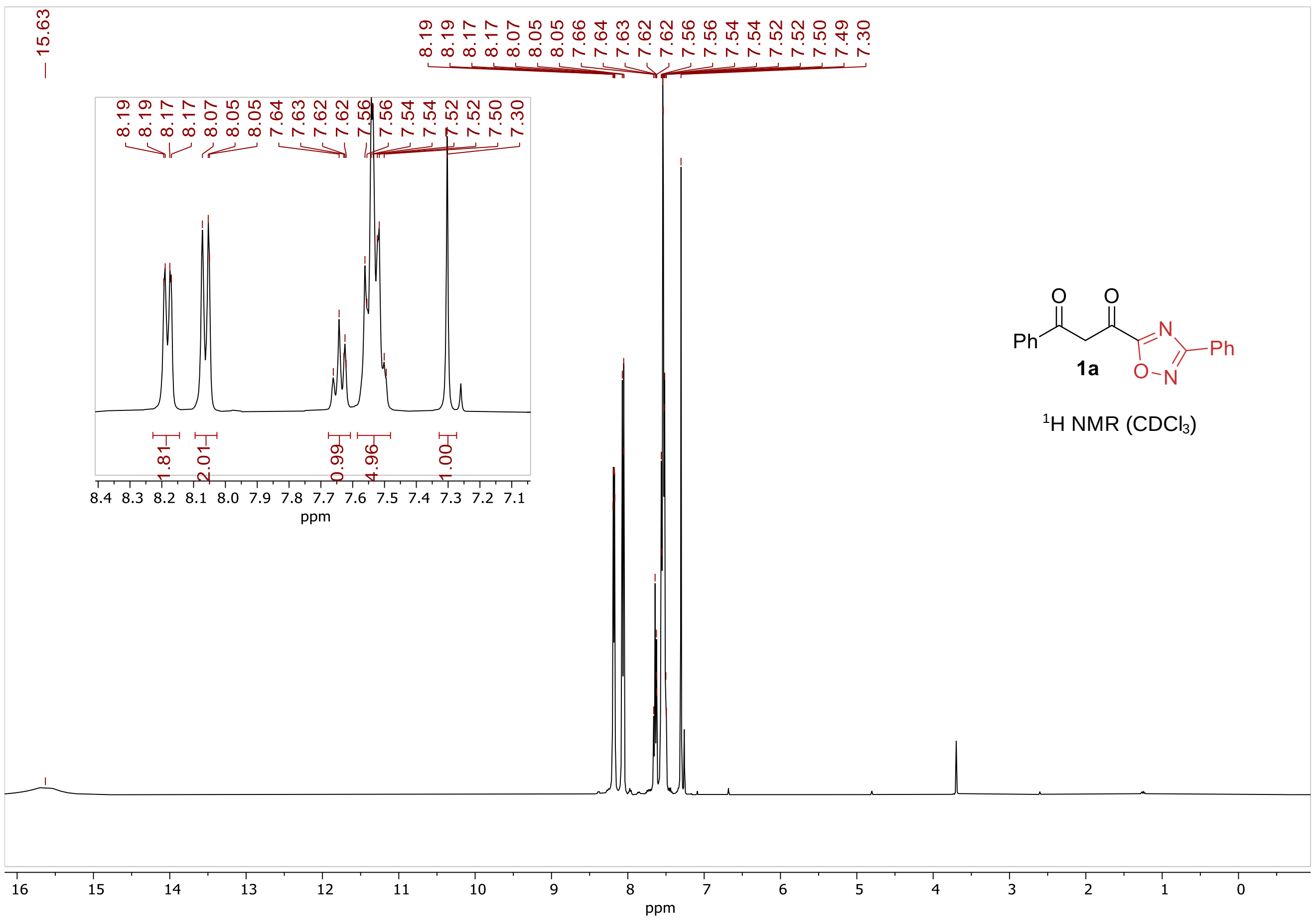

15.63
8.19 8.19 8.17 8.17 8.07 8.05 8.05 7.66 7.64 7.63 7.62 7.62 7.56 7.56 7.54 7.54 7.52 7.52 7.50 7.49 7.30
1.81
2.01
0.99
4.96
1.00
ppm
1a
Ph
O
N
Ph
^{1}H NMR ($CDCl_3$)

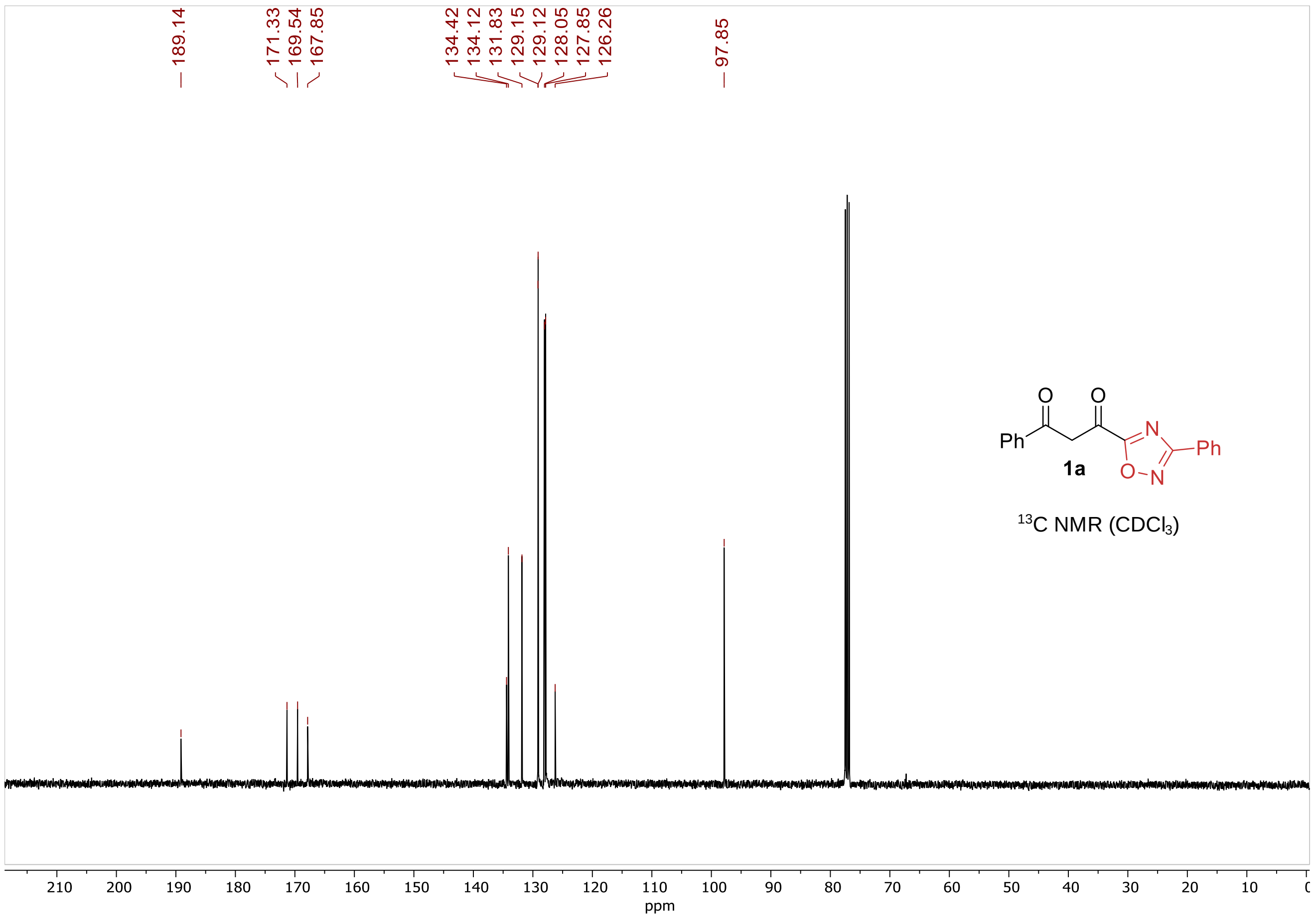

189.14
171.33 169.54 167.85
134.42 134.12 131.83 129.15 129.12 128.05 127.85 126.26
97.85
ppm
1a
^{13}C NMR ($CDCl_3$)

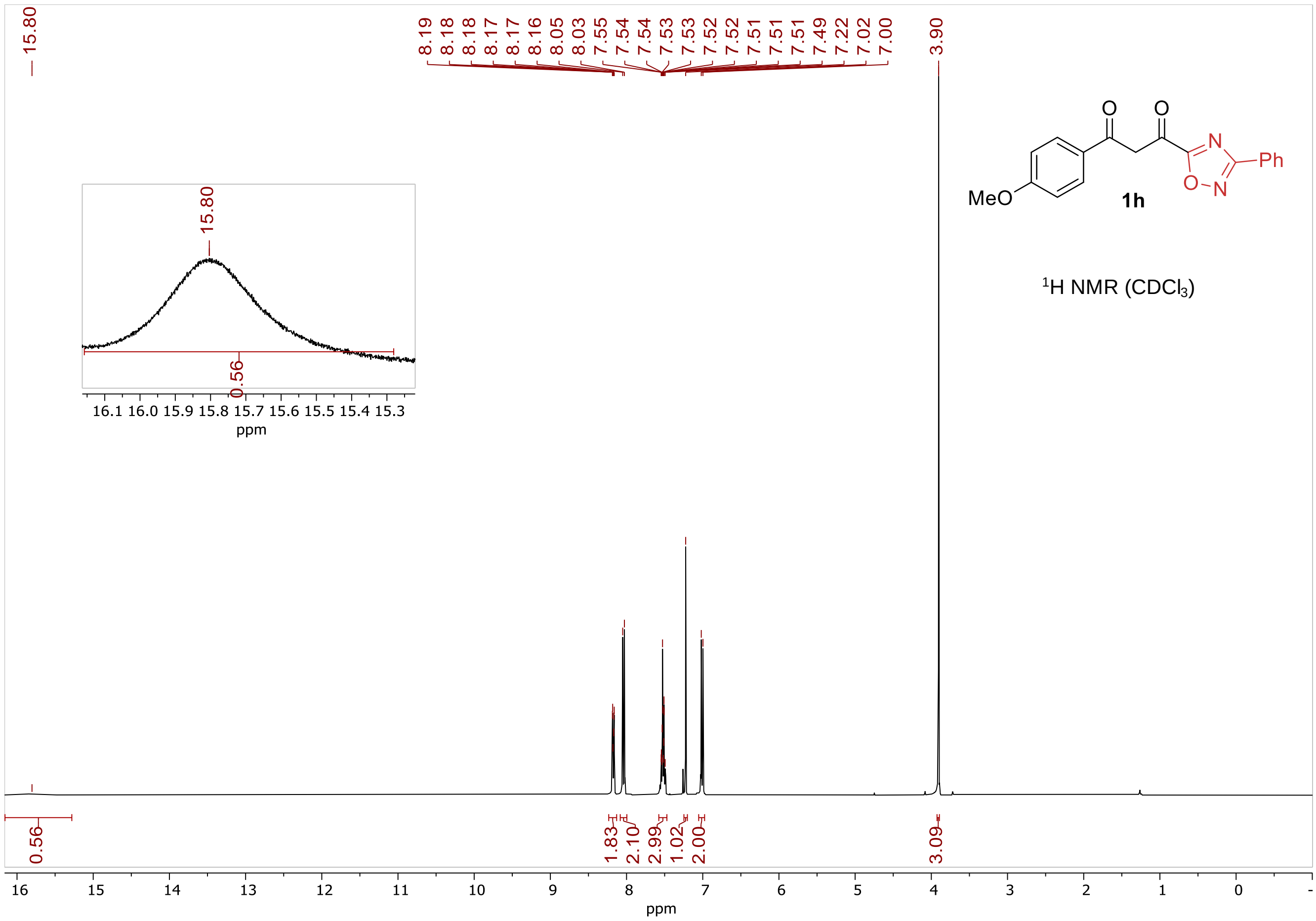

MeO
1h
Ph
^{1}H NMR (CDCl$_3$)
ppm

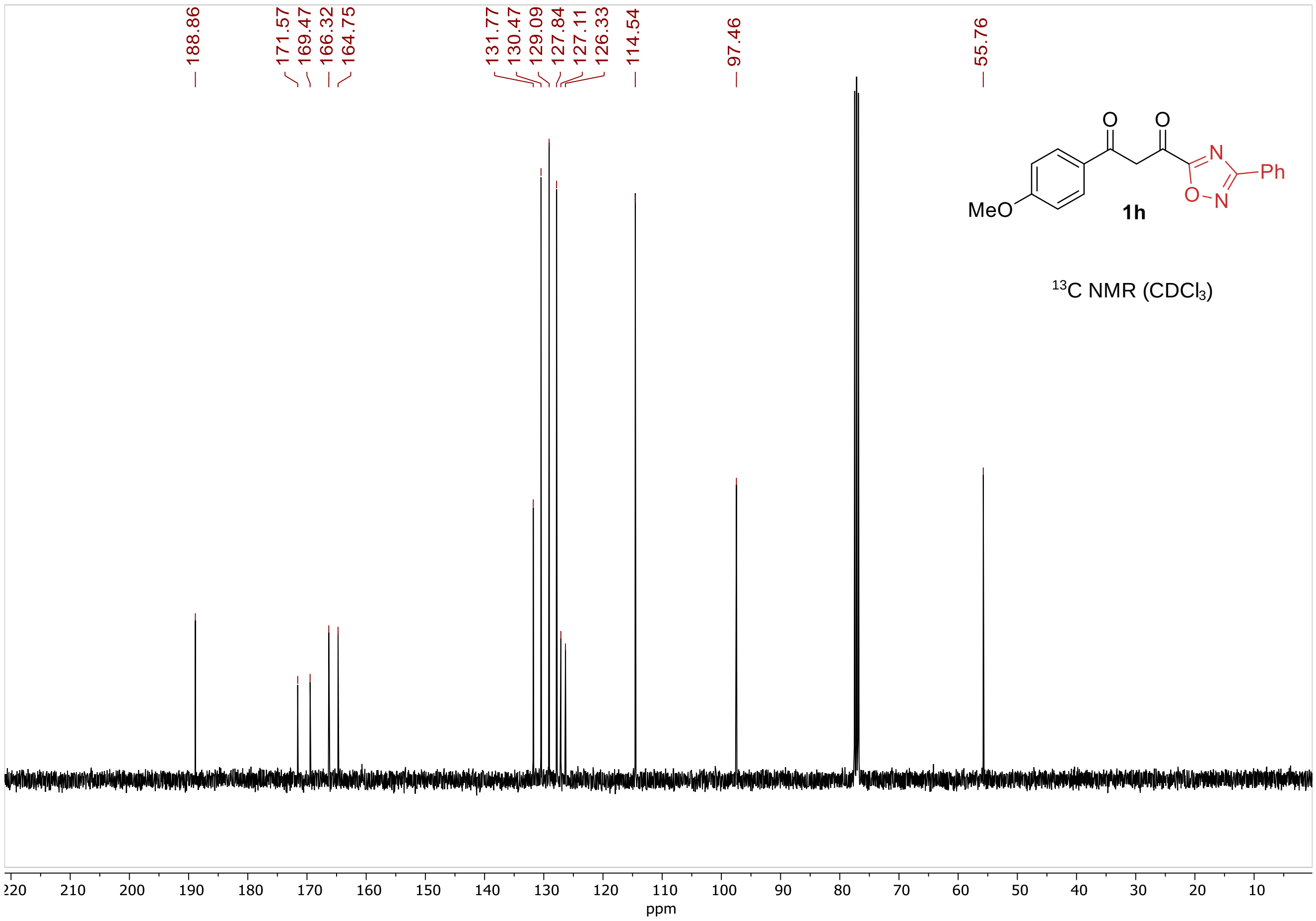

MeO
1h
Ph
^{13}C NMR (CDCl$_3$)
ppm

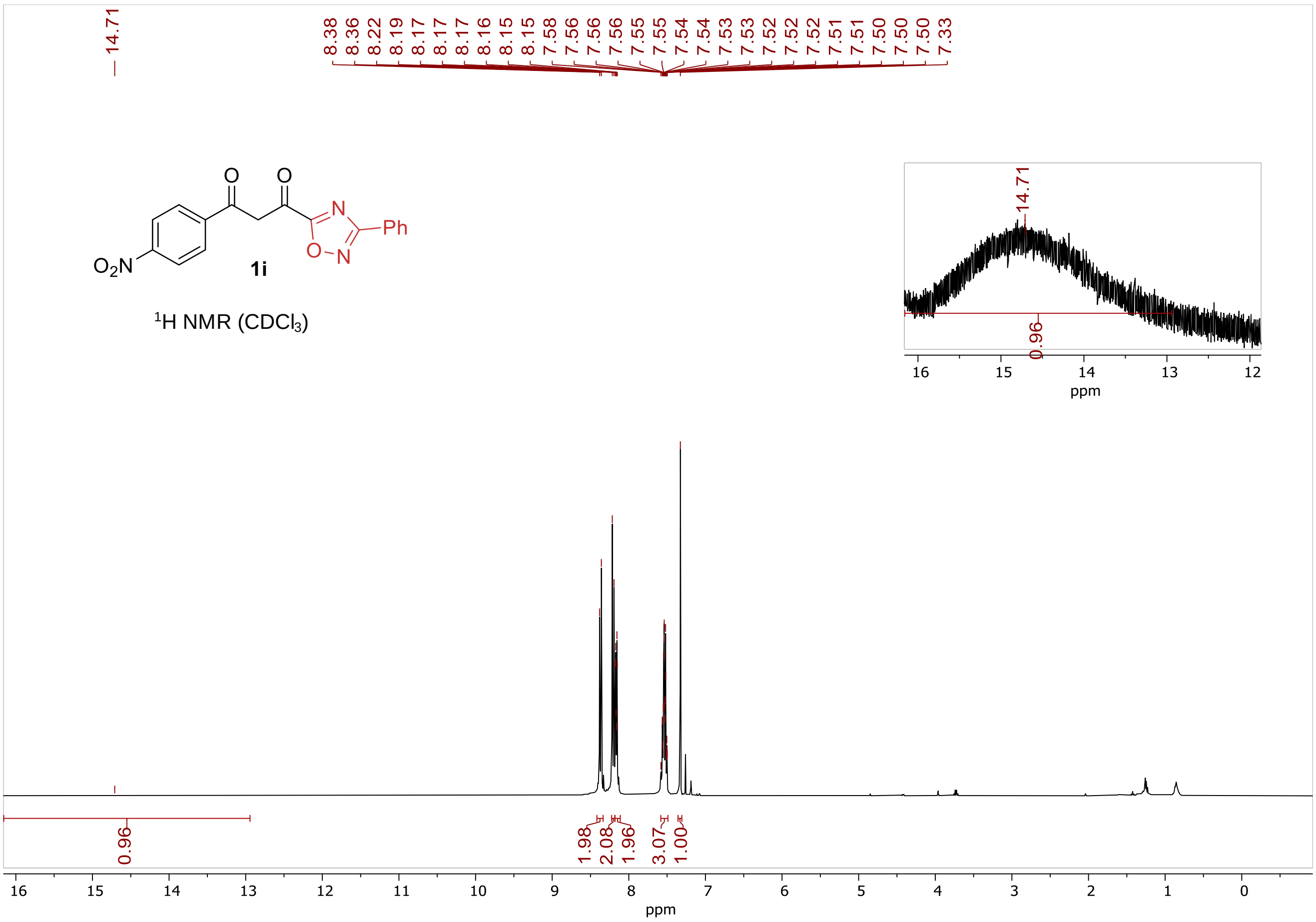
14.71
8.38 8.36 8.22 8.19 8.17 8.17 8.17 8.16 8.15 8.15 7.58 7.56 7.56 7.56 7.55 7.55 7.54 7.54 7.53 7.53 7.52 7.52 7.52 7.51 7.51 7.50 7.50 7.50 7.33
O
O
N
Ph
O
N
O2N
1i
1H NMR (CDCl3)
14.71
0.96
16
15
14
13
12
ppm
0.96
1.98
2.08
1.96
3.07
1.00
16 15 14 13 12 11 10 9 8 7 6 5 4 3 2 1 0
ppm

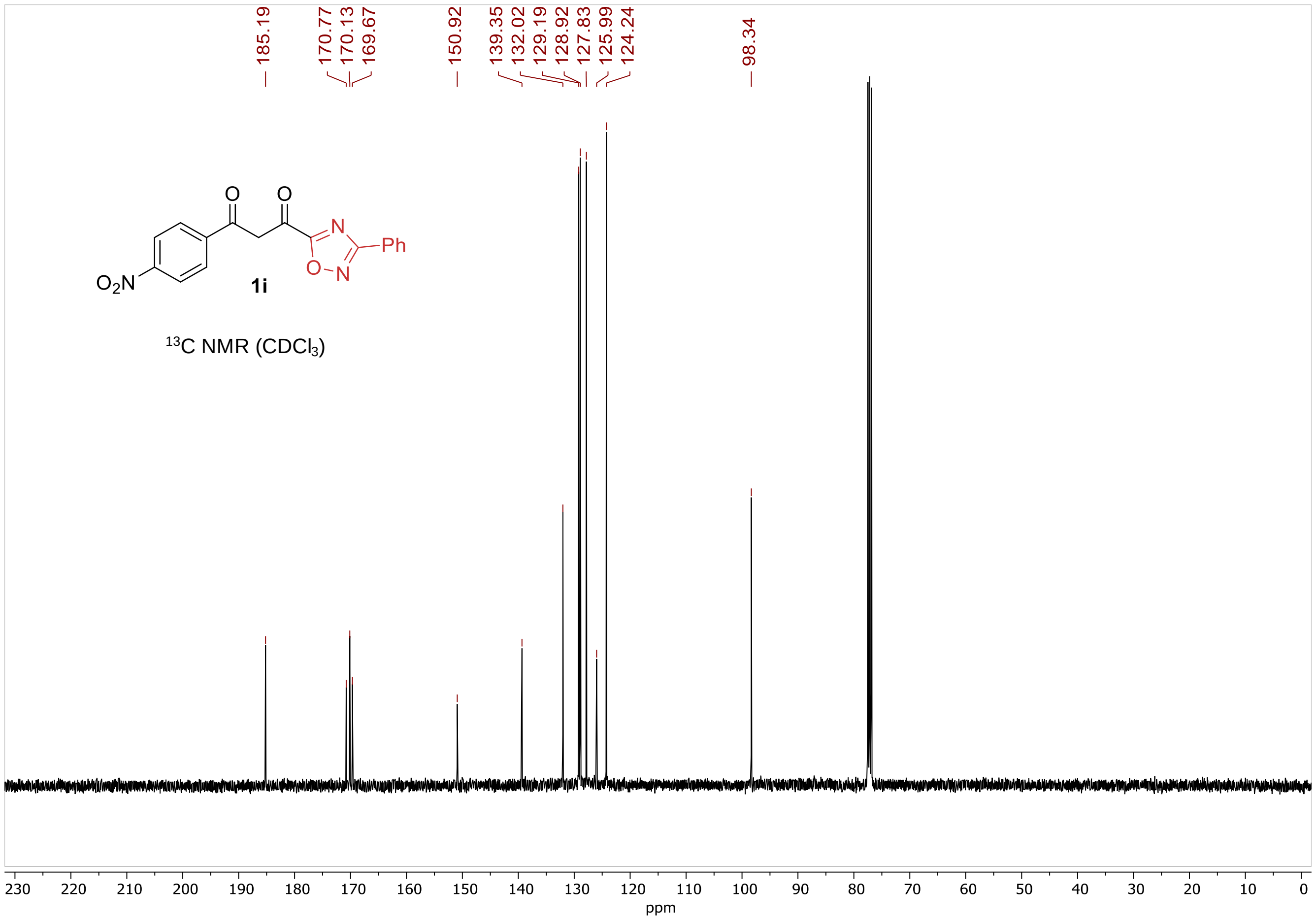
185.19
170.77 170.13 169.67
150.92
139.35 132.02 129.19 128.92 127.83 125.99 124.24
98.34
O
O
N
Ph
O
N
O2N
1i
13C NMR (CDCl3)
230 220 210 200 190 180 170 160 150 140 130 120 110 100 90 80 70 60 50 40 30 20 10 0
ppm

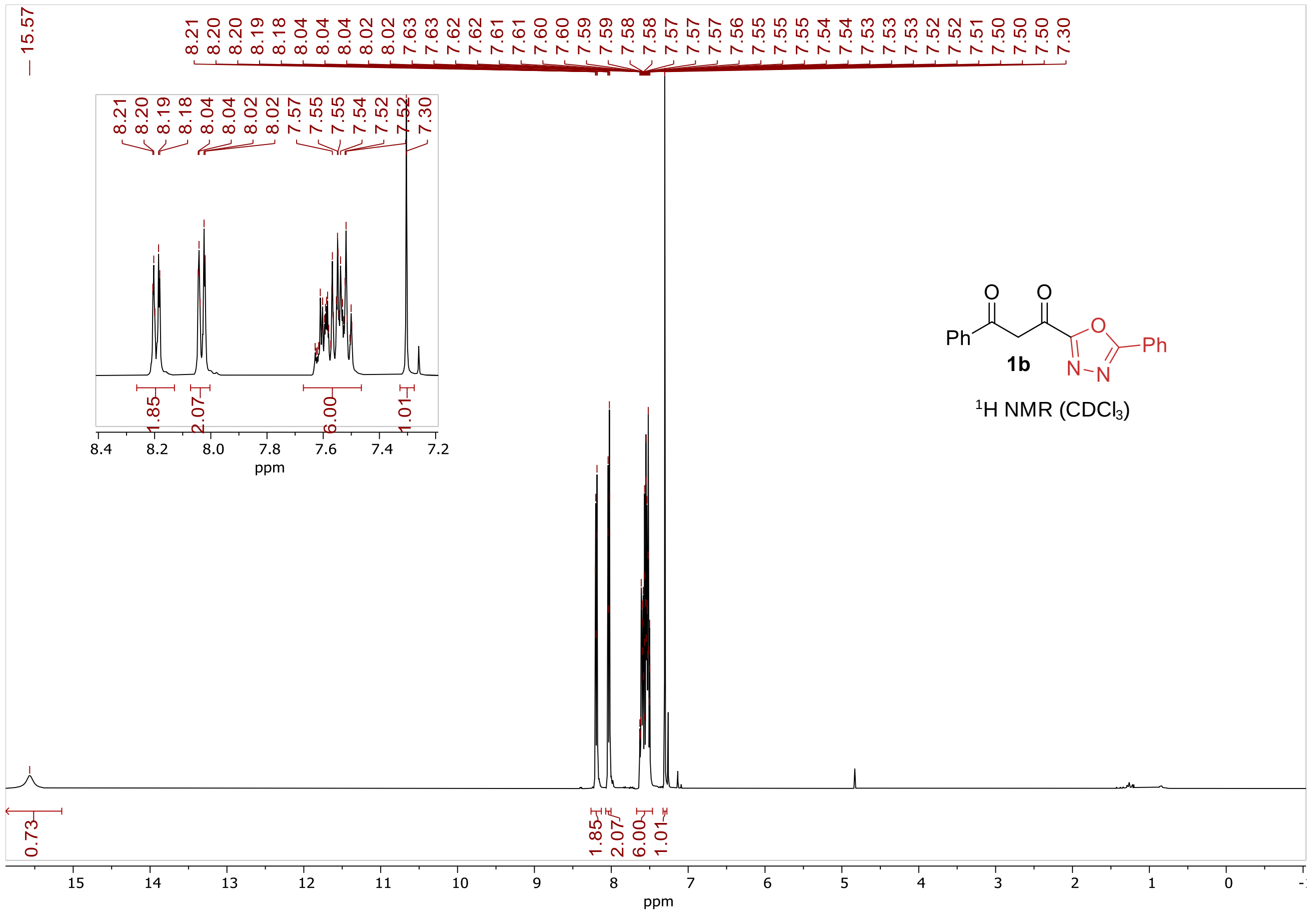
15.57
8.21 8.20 8.20 8.19 8.18 8.04 8.04 8.04 8.02 8.02 8.03 7.63 7.62 7.62 7.61 7.61 7.60 7.60 7.59 7.59 7.58 7.58 7.57 7.57 7.56 7.55 7.55 7.55 7.54 7.54 7.53 7.53 7.53 7.52 7.52 7.51 7.50 7.50 7.50 7.30
8.21 8.20 8.19 8.18 8.04 8.04 8.02 8.02 7.57 7.55 7.55 7.54 7.52 7.52 7.30
1.85 2.07 6.00 1.01
0.73
ppm
Ph
1b
Ph
¹H NMR (CDCl₃)

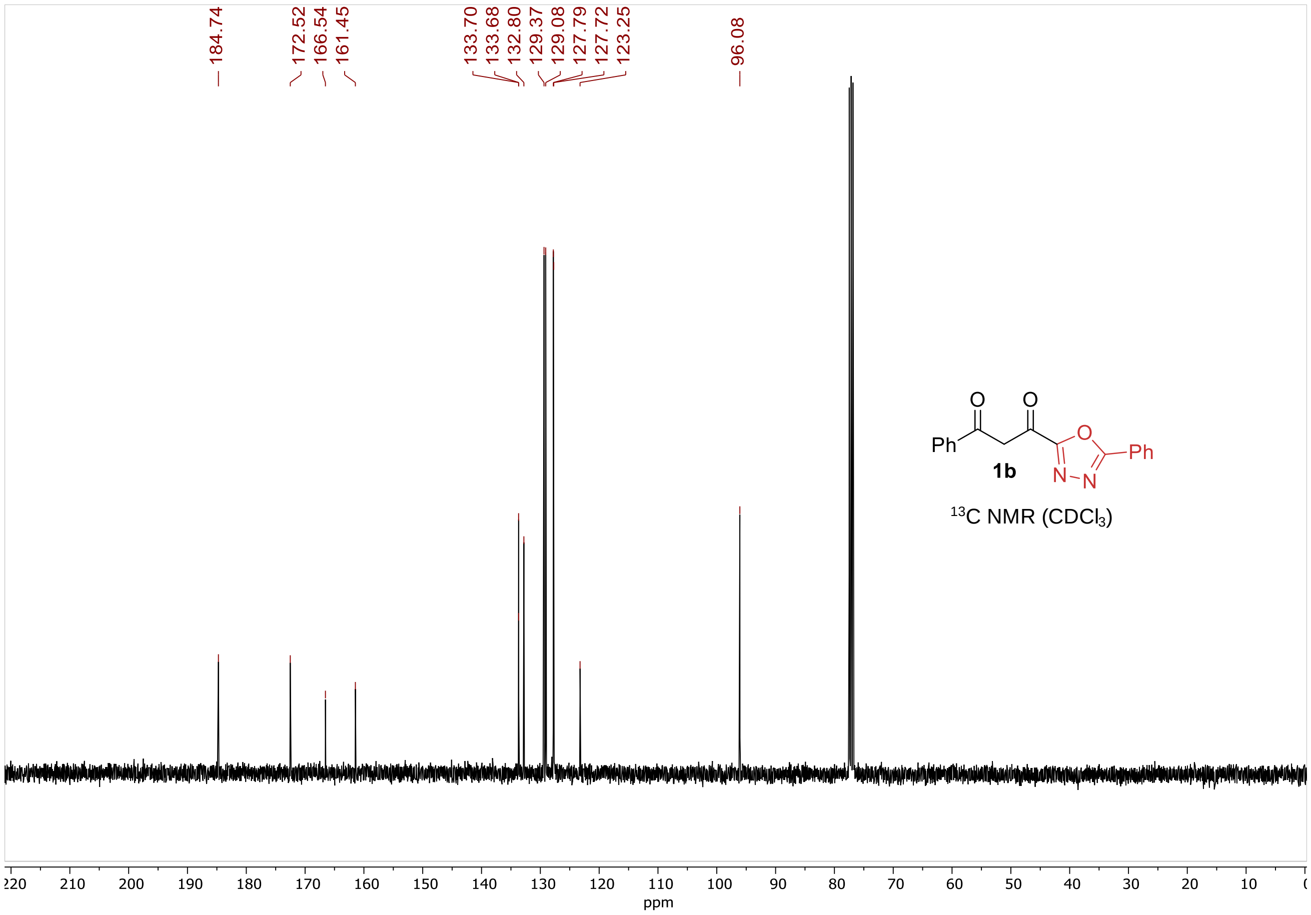
184.74
172.52 166.54 161.45
133.70 133.68 132.80 129.37 129.08 127.79 127.72 123.25
96.08
ppm
Ph
1b
Ph
¹³C NMR (CDCl₃)

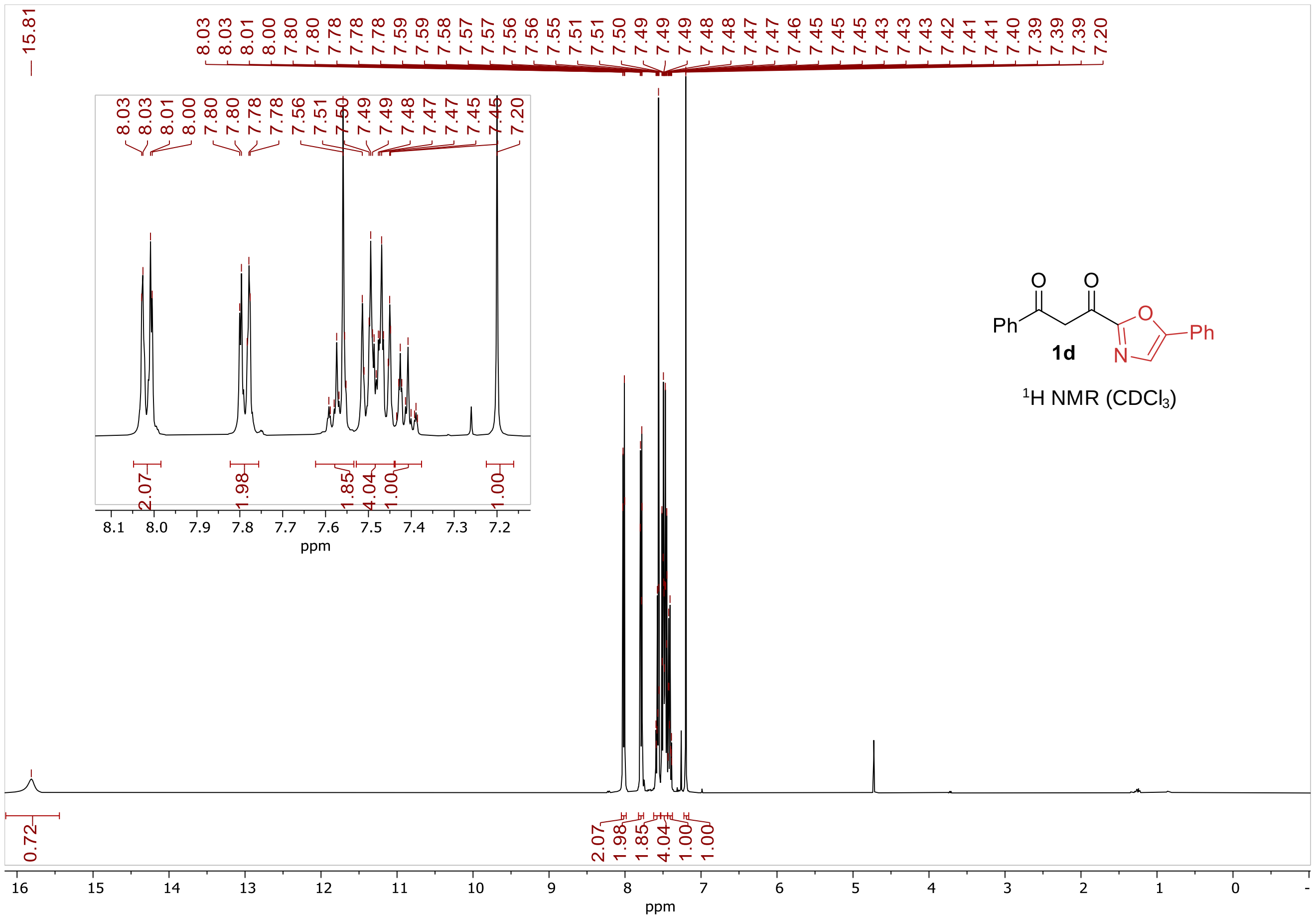

15.81
8.03
8.03
8.01
8.00
7.80
7.80
7.78
7.78
7.59
7.59
7.58
7.57
7.57
7.56
7.56
7.55
7.51
7.51
7.50
7.49
7.49
7.49
7.48
7.48
7.47
7.47
7.46
7.45
7.45
7.45
7.43
7.43
7.43
7.42
7.41
7.41
7.40
7.39
7.39
7.39
7.20
2.07
1.98
1.85
4.04
1.00
1.00
0.72
ppm
Ph
O
O
O
N
Ph
1d
^{1}H NMR ($CDCl_3$)

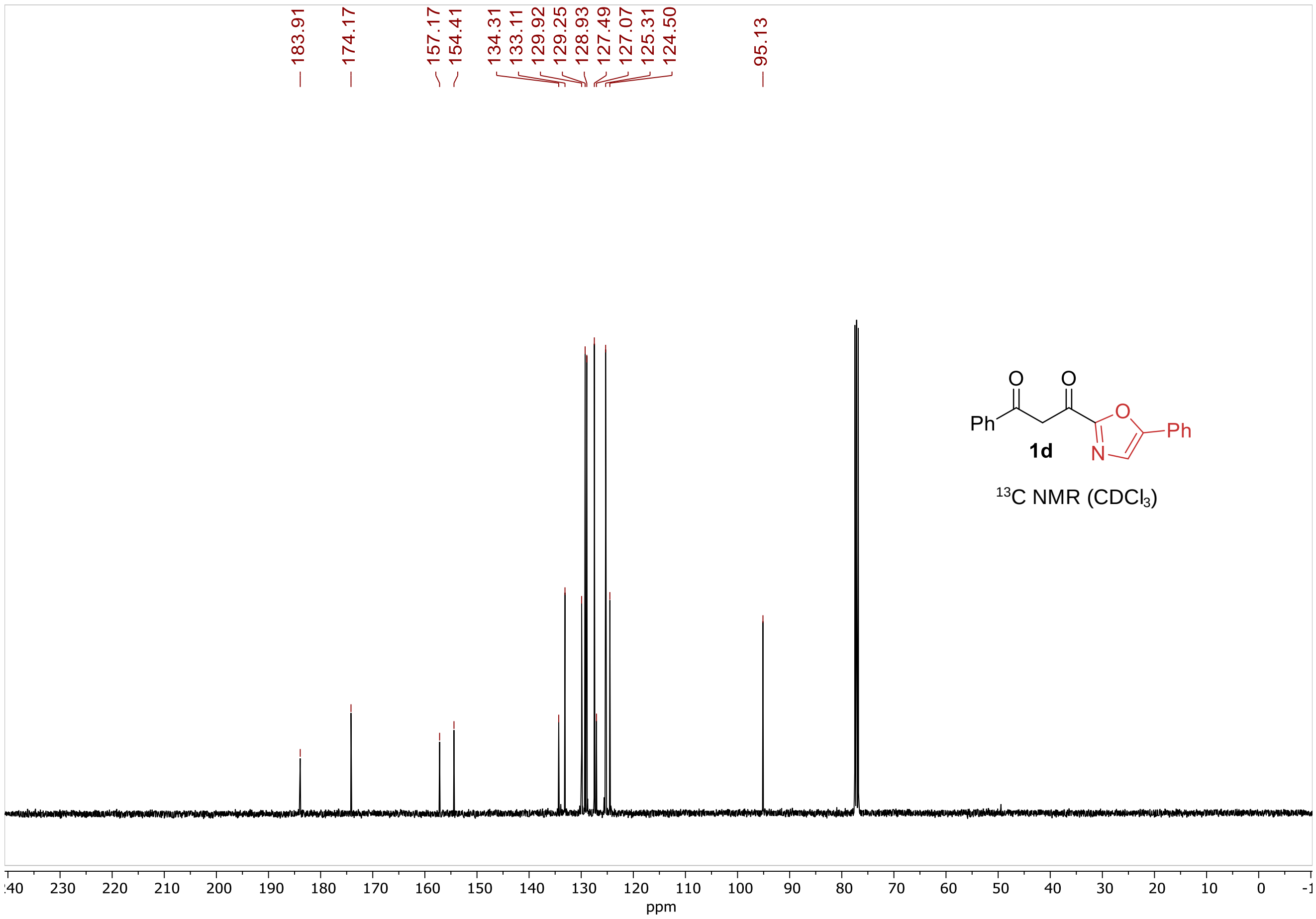

183.91
174.17
157.17
154.41
134.31
133.11
129.92
129.25
128.93
127.49
127.07
125.31
124.50
95.13
ppm
Ph
O
O
O
N
Ph
1d
^{13}C NMR ($CDCl_3$)

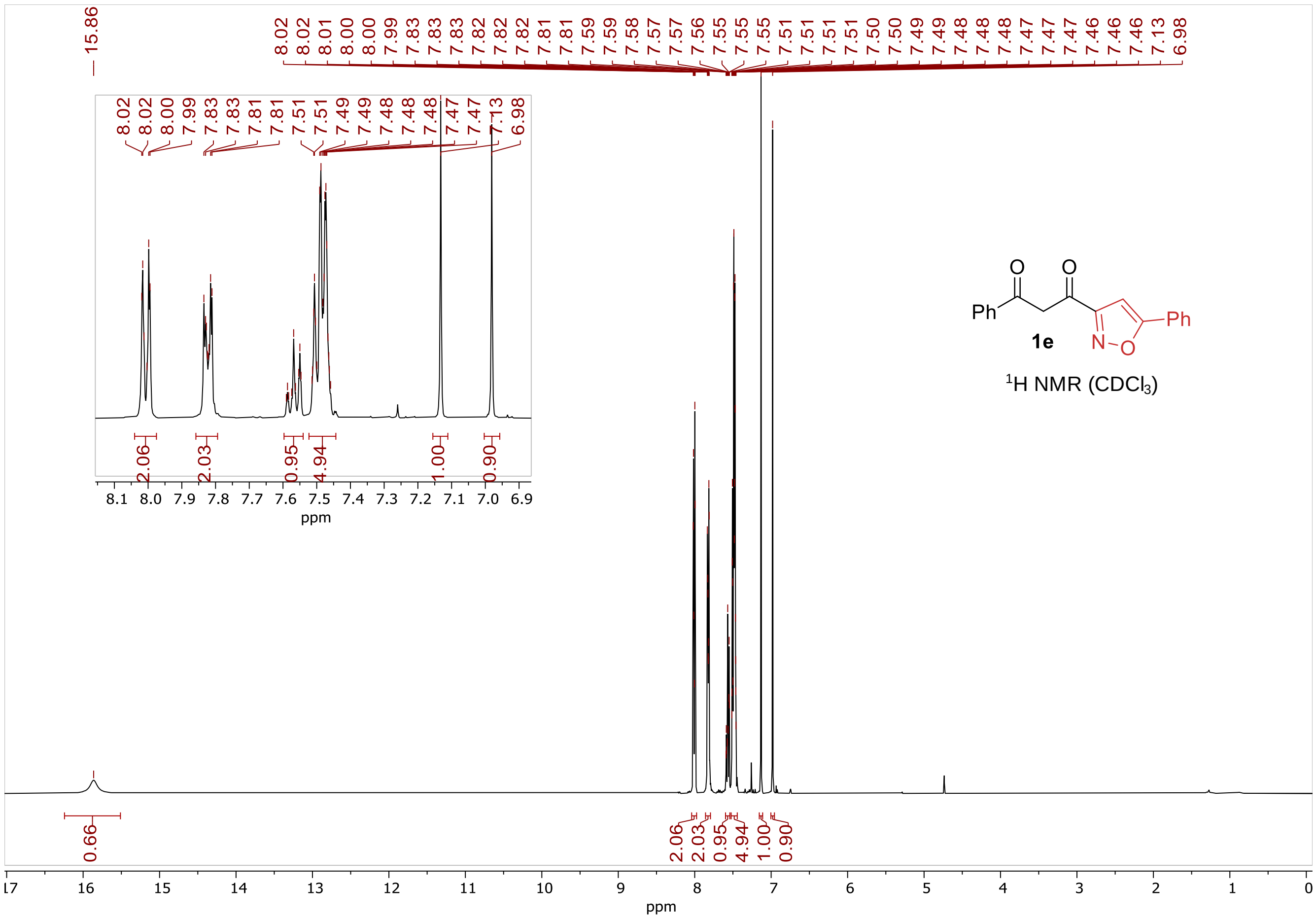
15.86
8.02 8.02 8.01 8.00 8.00 7.99 7.83 7.83 7.82 7.82 7.82 7.81 7.81 7.59 7.59 7.58 7.57 7.57 7.56 7.55 7.55 7.55 7.51 7.51 7.51 7.50 7.49 7.49 7.48 7.48 7.48 7.47 7.47 7.47 7.46 7.46 7.46 7.13 6.98
Ph
1e
N
O
Ph
^{1}H NMR (CDCl$_3$)
0.66
2.06 2.03 0.95 4.94 1.00 0.90
ppm

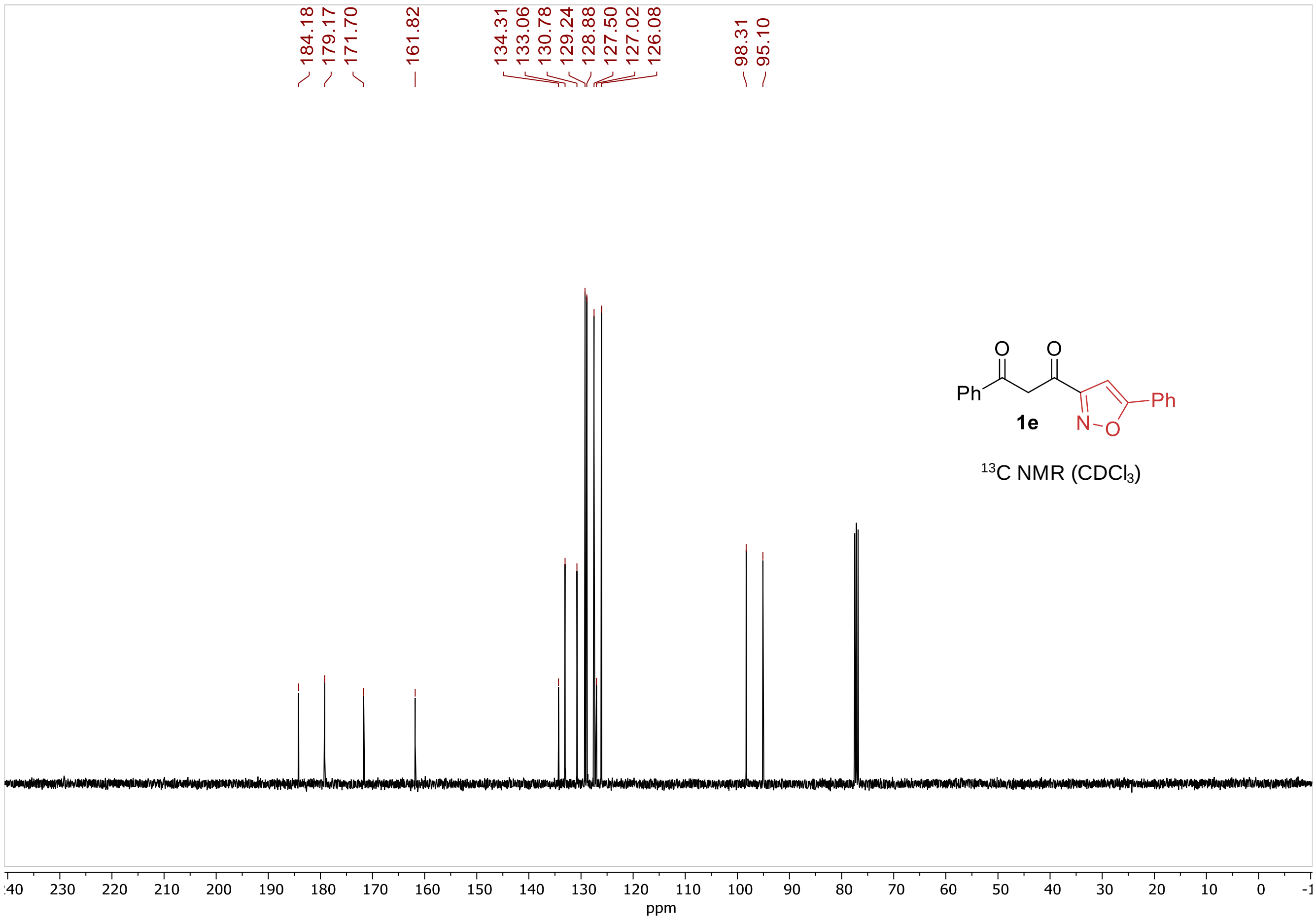
184.18 179.17 171.70 161.82
134.31 133.06 130.78 129.24 128.88 127.50 127.02 126.08
98.31 95.10
Ph
1e
N
O
Ph
^{13}C NMR (CDCl$_3$)
ppm

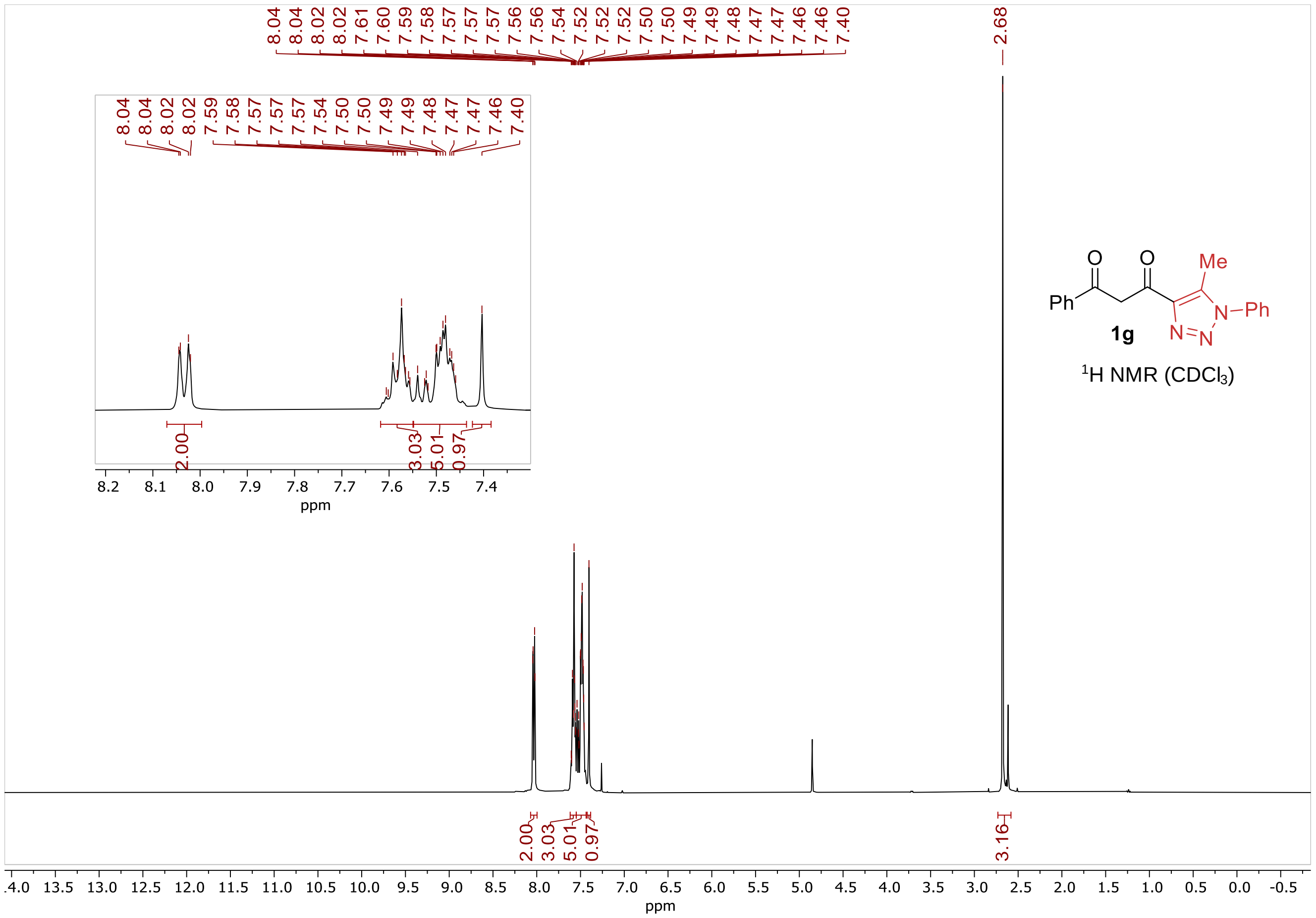
8.04 8.04 8.02 8.02 7.61 7.60 7.59 7.58 7.57 7.57 7.57 7.56 7.56 7.54 7.52 7.52 7.52 7.50 7.50 7.49 7.49 7.48 7.47 7.47 7.46 7.46 7.40
2.68
8.04 8.04 8.02 8.02 7.59 7.58 7.57 7.57 7.57 7.54 7.50 7.50 7.49 7.49 7.48 7.47 7.47 7.46 7.40
2.00
3.03
5.01
0.97
3.16
ppm
O
O
Me
Ph
N–Ph
N=N
1g
1H NMR (CDCl3)

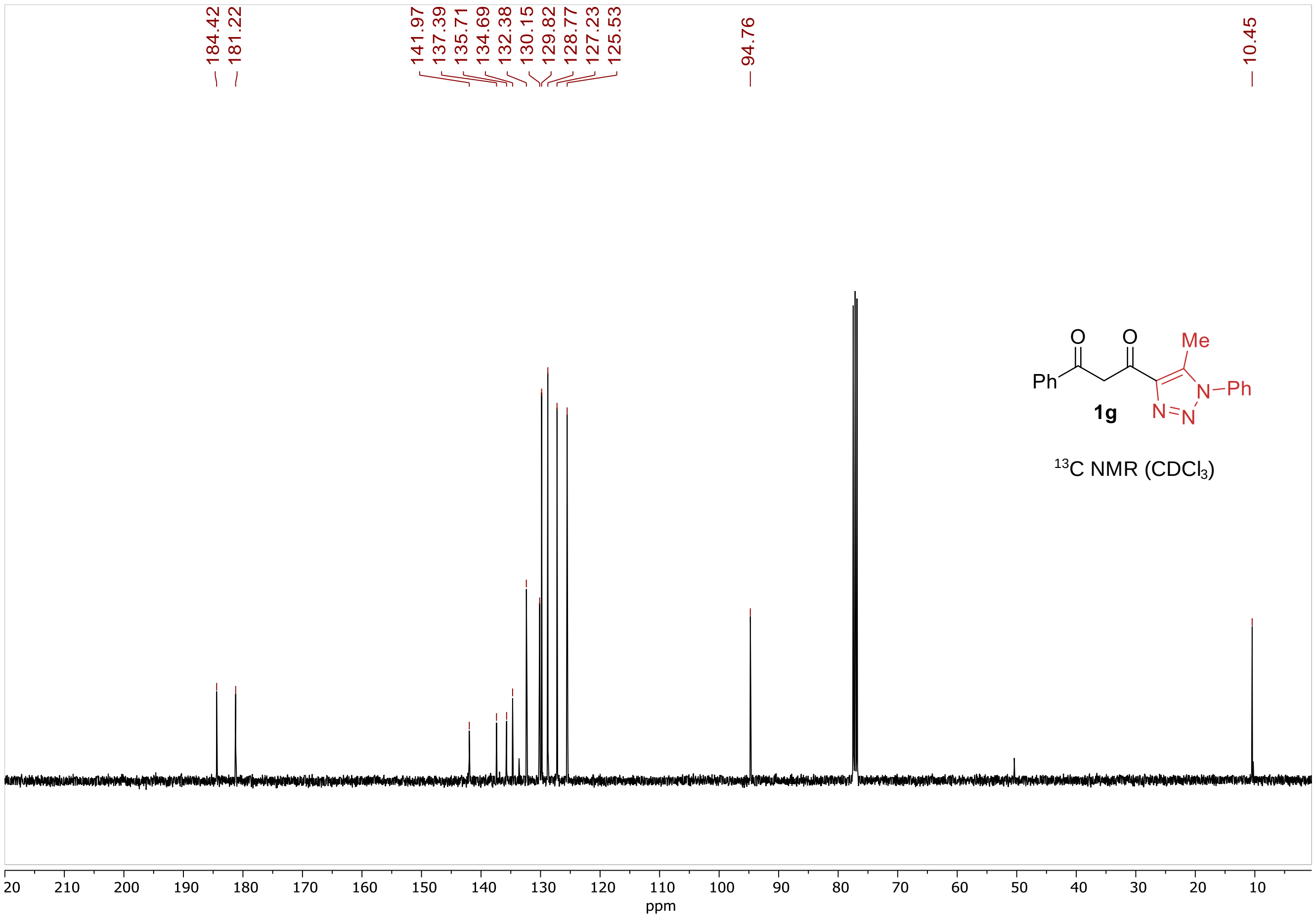
184.42
181.22
141.97 137.39 135.71 134.69 132.38 130.15 129.82 128.77 127.23 125.53
94.76
10.45
ppm
O
O
Me
Ph
N–Ph
N=N
1g
13C NMR (CDCl3)

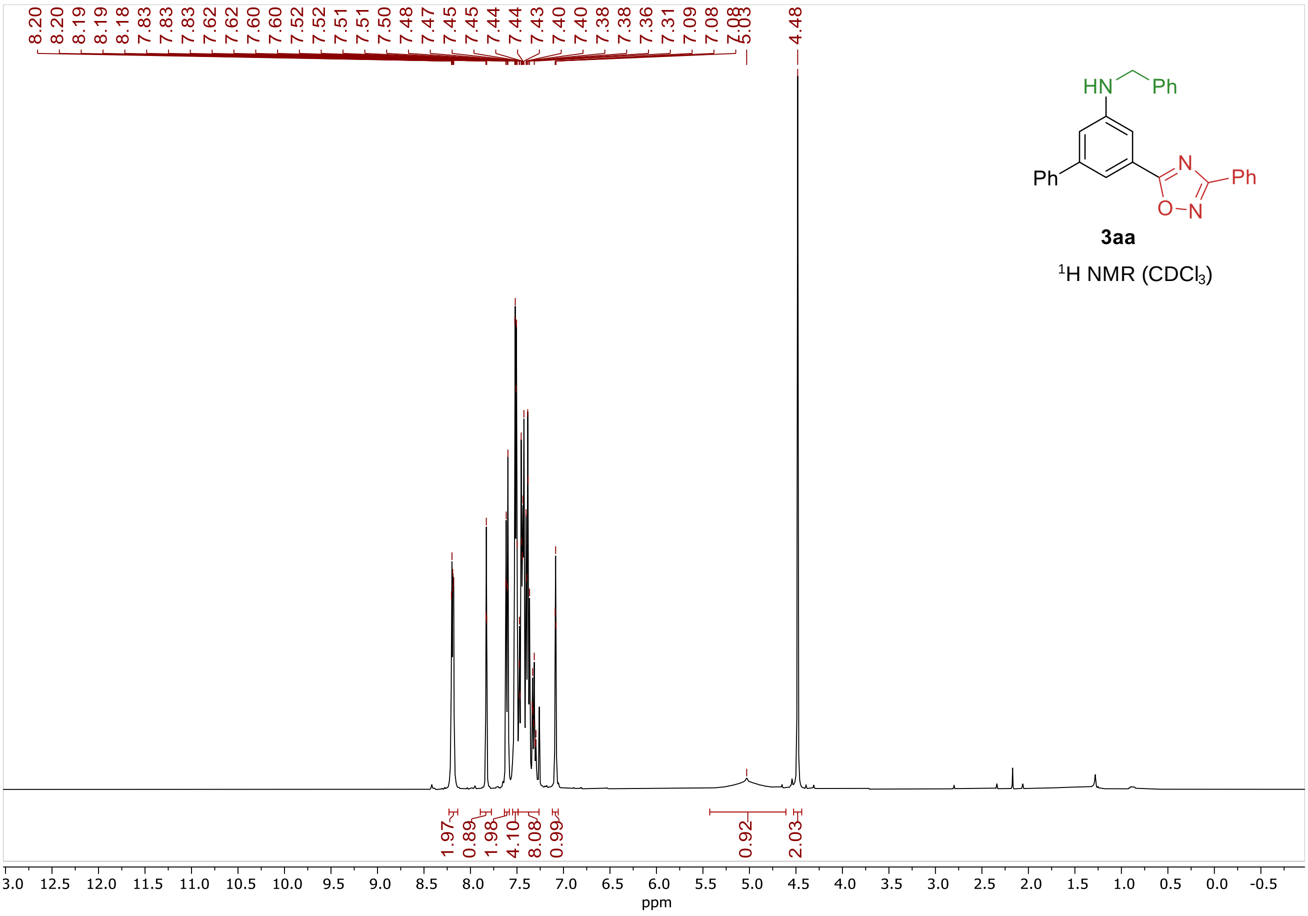
8.20 8.20 8.19 8.19 8.18 7.83 7.83 7.83 7.62 7.62 7.60 7.60 7.52 7.52 7.51 7.51 7.50 7.48 7.47 7.45 7.45 7.44 7.44 7.43 7.40 7.40 7.38 7.38 7.36 7.31 7.09 7.08 7.08 5.03
4.48
HN
Ph
Ph
N
Ph
O
N
3aa
^{1}H NMR ($CDCl_3$)
1.97 0.89 1.98 4.10 8.08 0.99
0.92
2.03
ppm

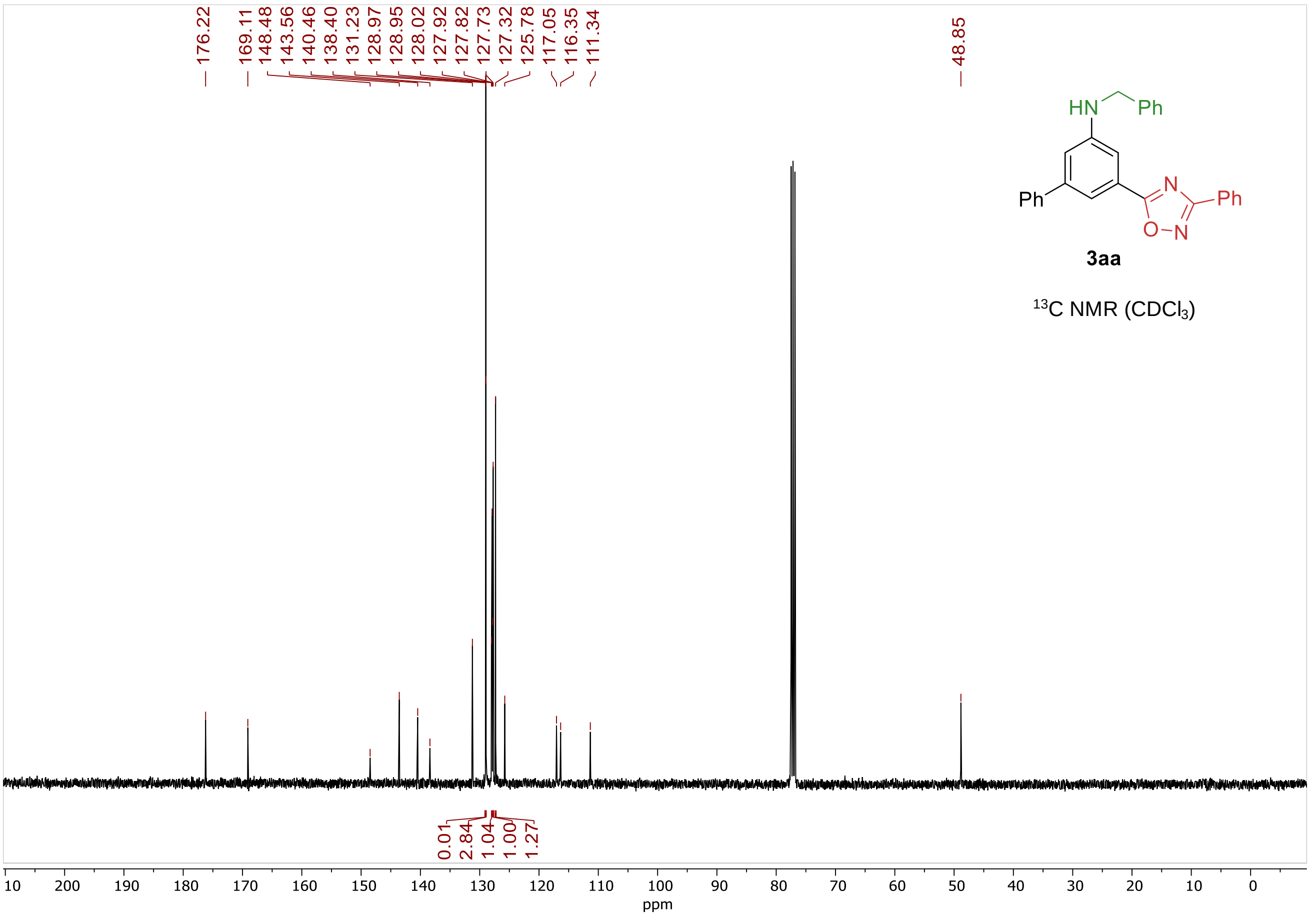
176.22
169.11 148.48 143.56 140.46 138.40 131.23 128.97 128.95 128.02 127.92 127.82 127.73 127.32 125.78 117.05 116.35 111.34
48.85
HN
Ph
Ph
N
Ph
O
N
3aa
^{13}C NMR ($CDCl_3$)
0.01 2.84 1.04 1.00 1.27
ppm

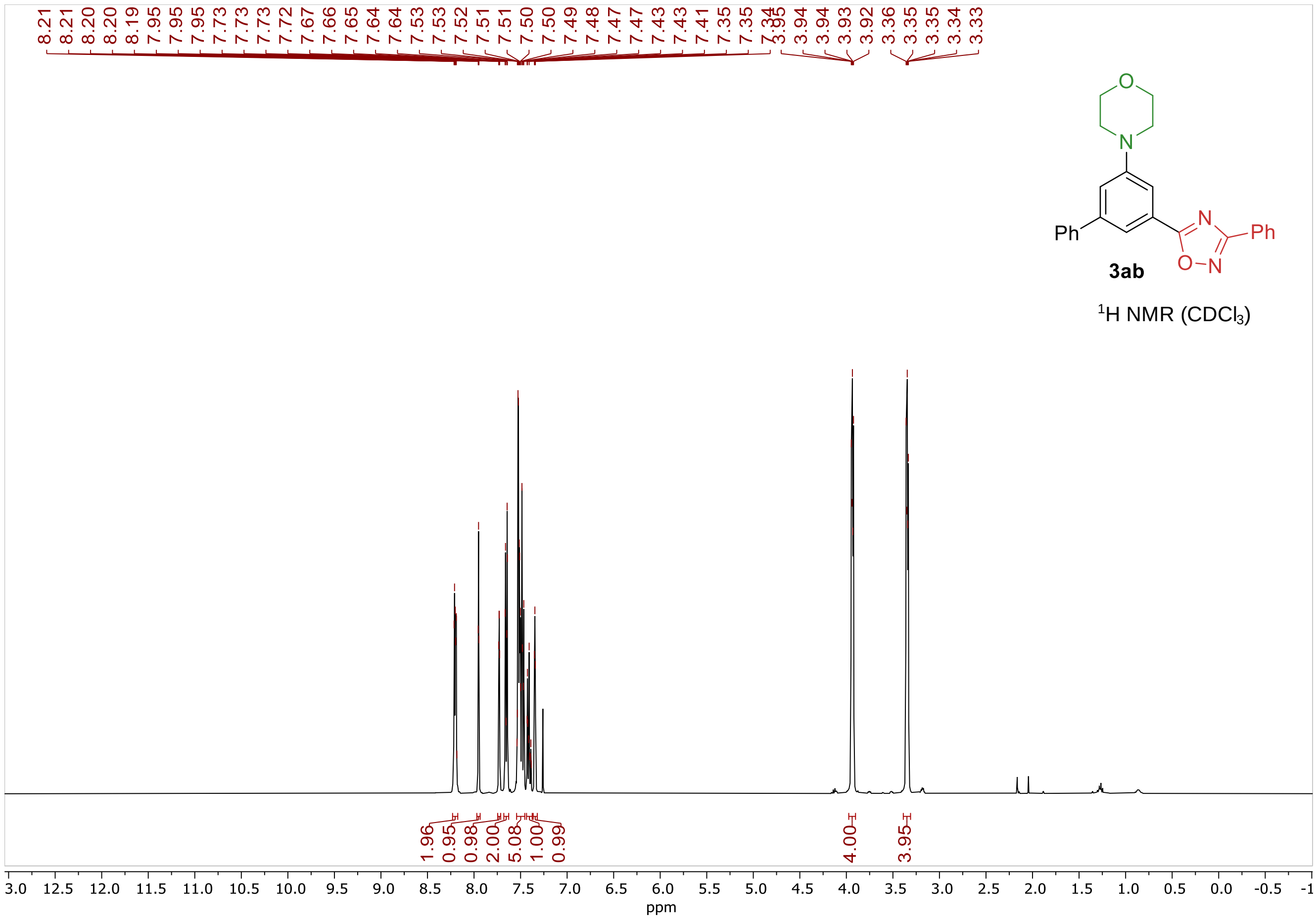
8.21 8.21 8.20 8.20 8.19 7.95 7.95 7.95 7.73 7.73 7.73 7.73 7.72 7.67 7.66 7.65 7.64 7.64 7.53 7.53 7.52 7.51 7.51 7.50 7.50 7.49 7.48 7.47 7.47 7.43 7.43 7.41 7.35 7.35 7.34 3.95 3.94 3.94 3.93 3.92 3.36 3.35 3.35 3.34 3.33
O
N
Ph
N
Ph
O–N
3ab
1H NMR (CDCl3)
1.96 0.95 0.98 2.00 5.08 1.00 0.99
4.00
3.95
ppm

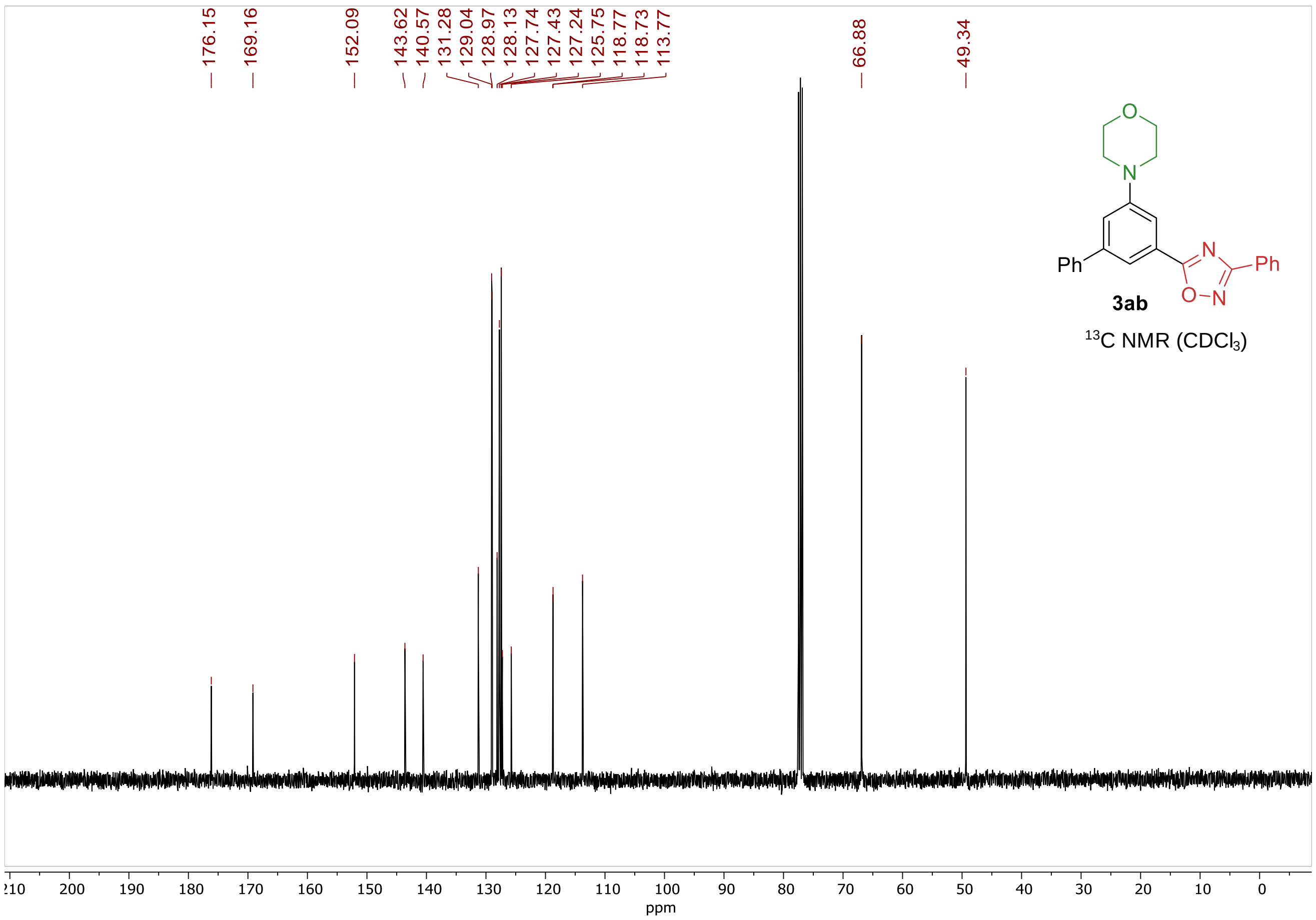
176.15
169.16
152.09
143.62 140.57 131.28 129.04 128.97 128.13 127.74 127.43 127.24 125.75 118.77 118.73 113.77
66.88
49.34
O
N
Ph
N
Ph
O–N
3ab
13C NMR (CDCl3)
ppm

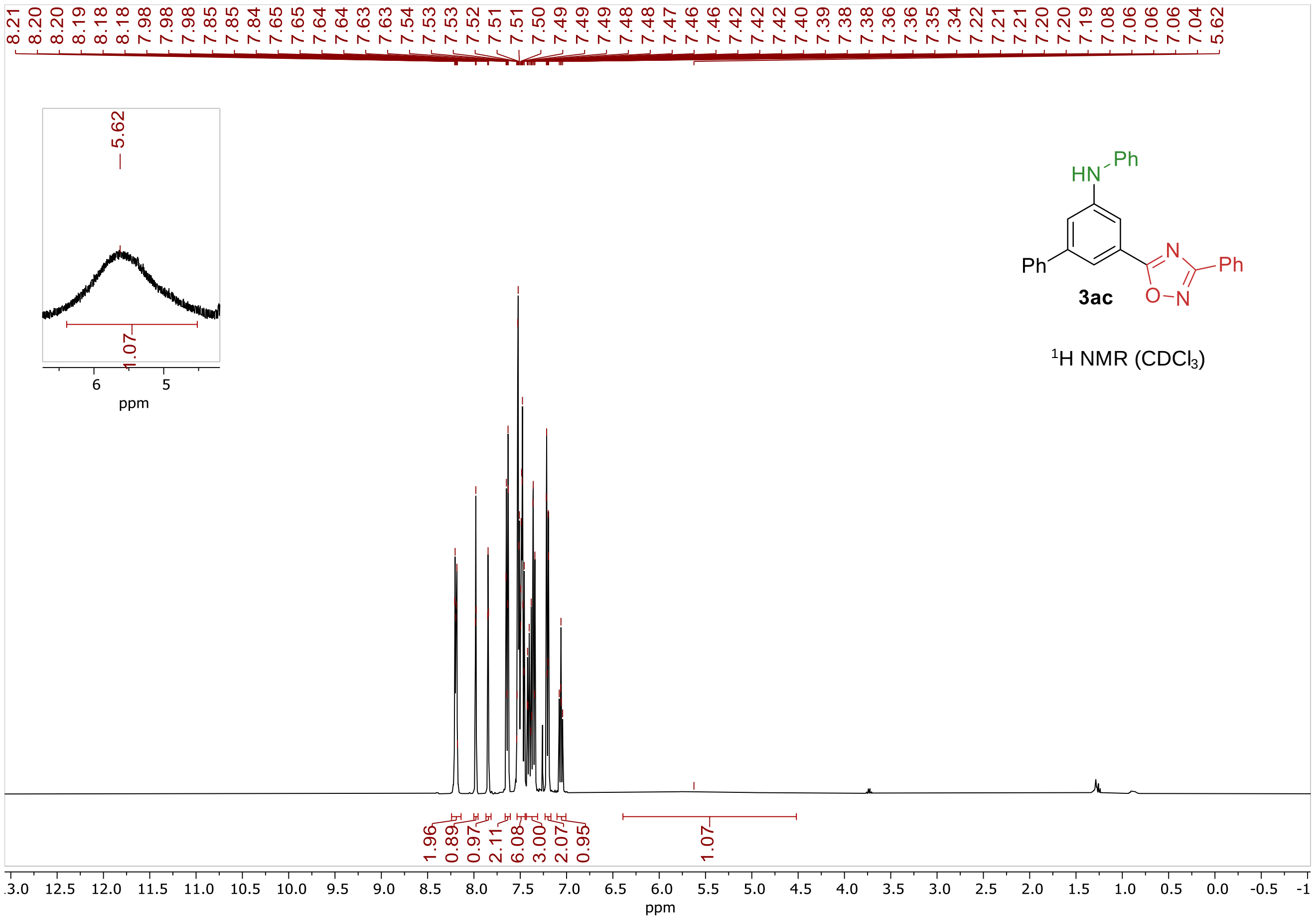

8.21 8.20 8.20 8.19 8.18 8.18 7.98 7.98 7.98 7.85 7.85 7.84 7.65 7.65 7.64 7.64 7.63 7.63 7.54 7.53 7.53 7.52 7.51 7.51 7.50 7.49 7.49 7.49 7.48 7.48 7.47 7.46 7.46 7.42 7.42 7.42 7.40 7.39 7.38 7.38 7.36 7.36 7.35 7.34 7.22 7.21 7.21 7.20 7.20 7.19 7.08 7.06 7.06 7.06 7.04 5.62
5.62
1.07
ppm
HN
Ph
Ph
N
Ph
O
N
3ac
^{1}H NMR ($CDCl_3$)
1.96 0.89 0.97 2.11 6.08 3.00 2.07 0.95
1.07
ppm

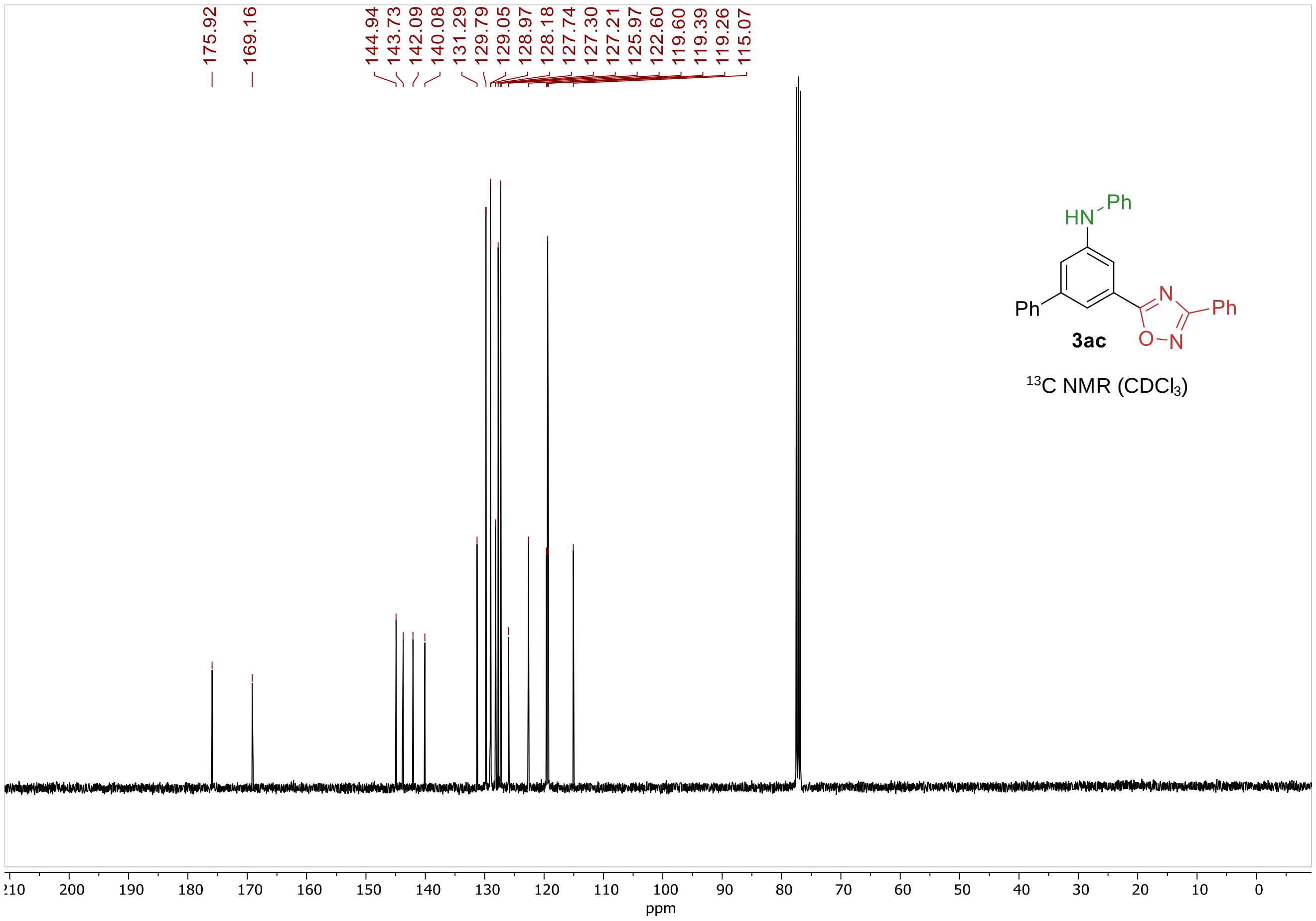

175.92
169.16
144.94 143.73 142.09 140.08 131.29 129.79 129.05 128.97 128.18 127.74 127.30 127.21 125.97 122.60 119.60 119.39 119.26 115.07
HN
Ph
Ph
N
Ph
O
N
3ac
^{13}C NMR ($CDCl_3$)
ppm

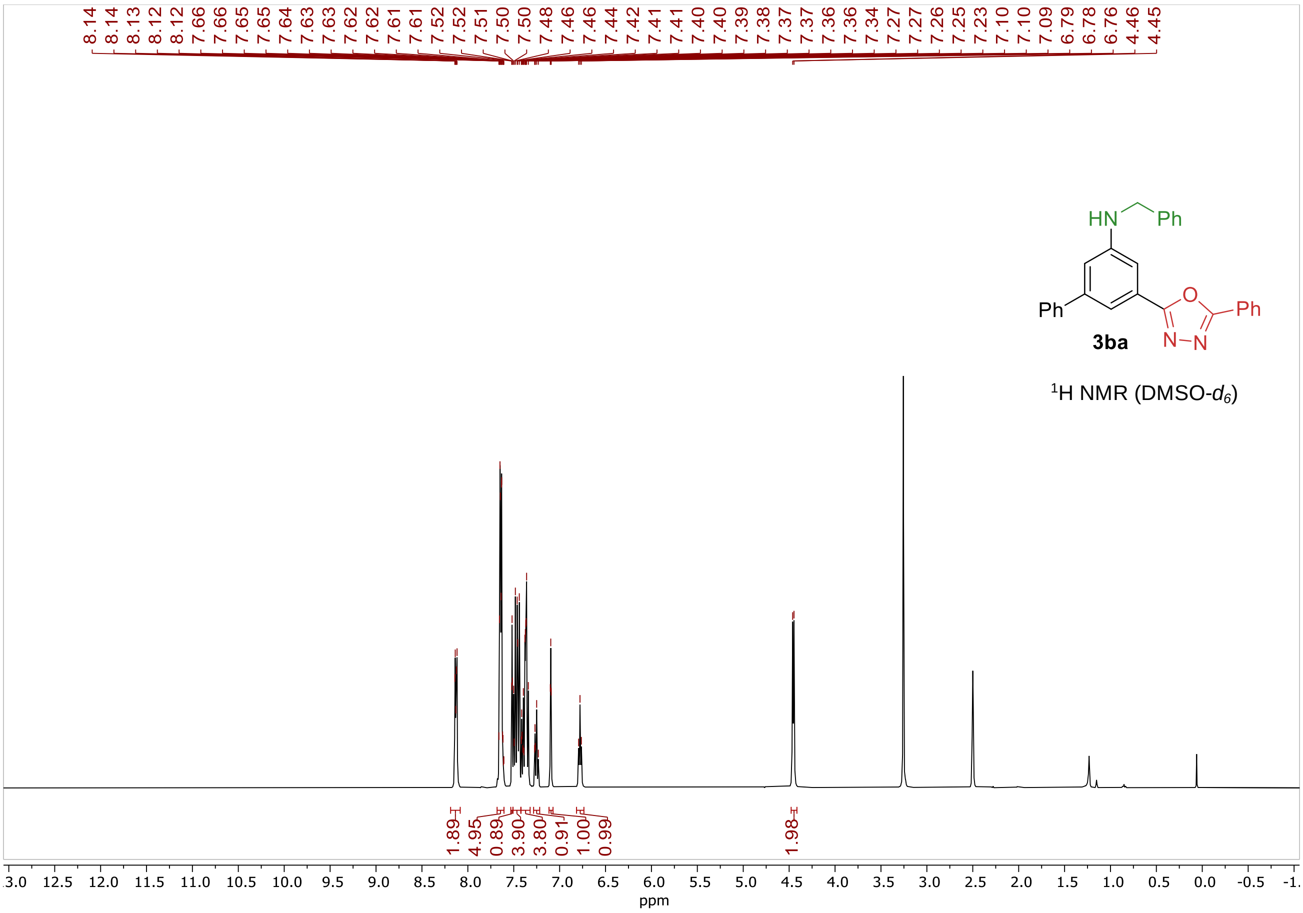
3ba
¹H NMR (DMSO-d6)
ppm

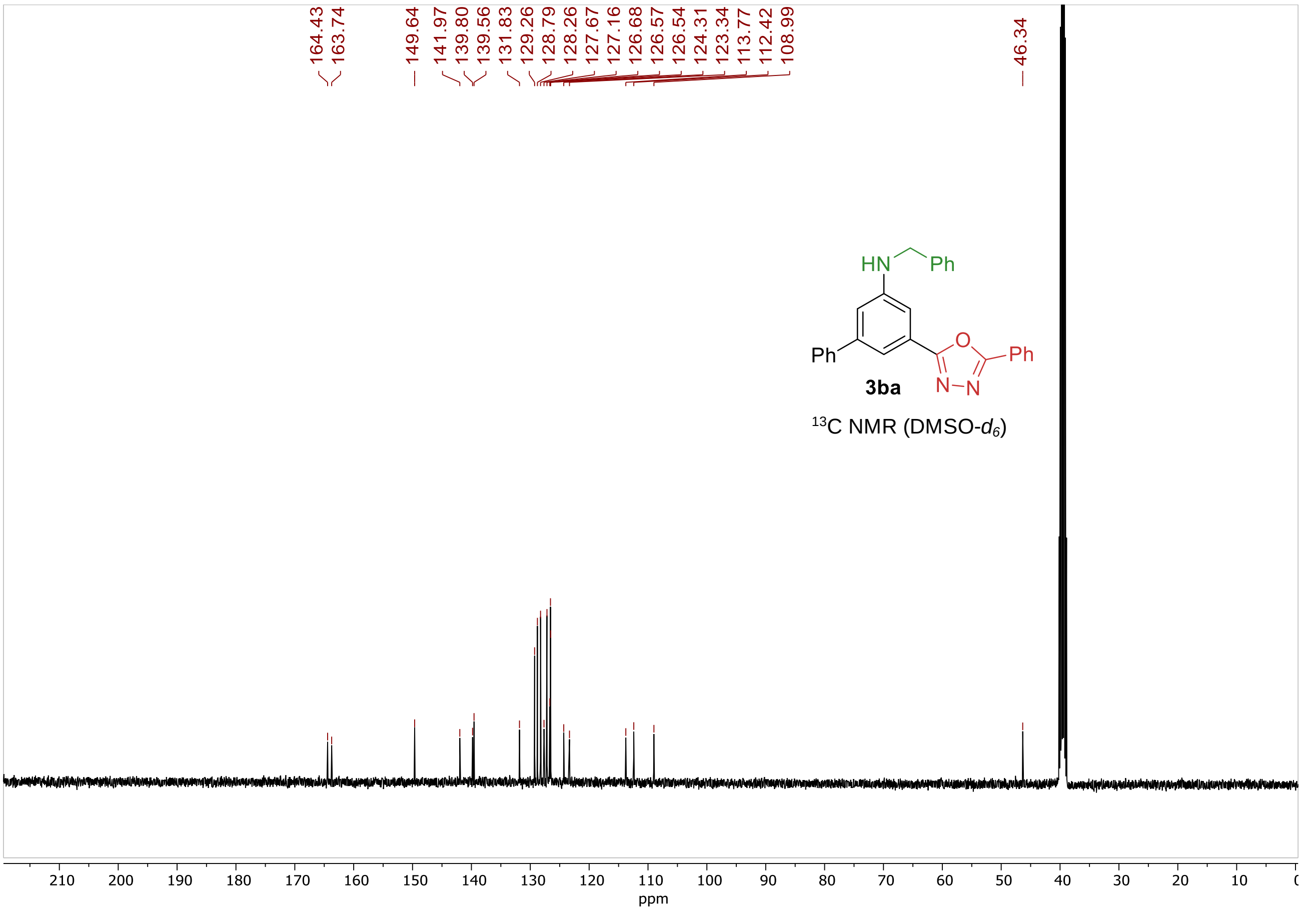
164.43
163.74
149.64
141.97
139.80
139.56
131.83
129.26
128.79
128.26
127.67
127.16
126.68
126.57
126.54
124.31
123.34
113.77
112.42
108.99
46.34
3ba
¹³C NMR (DMSO-d6)
ppm

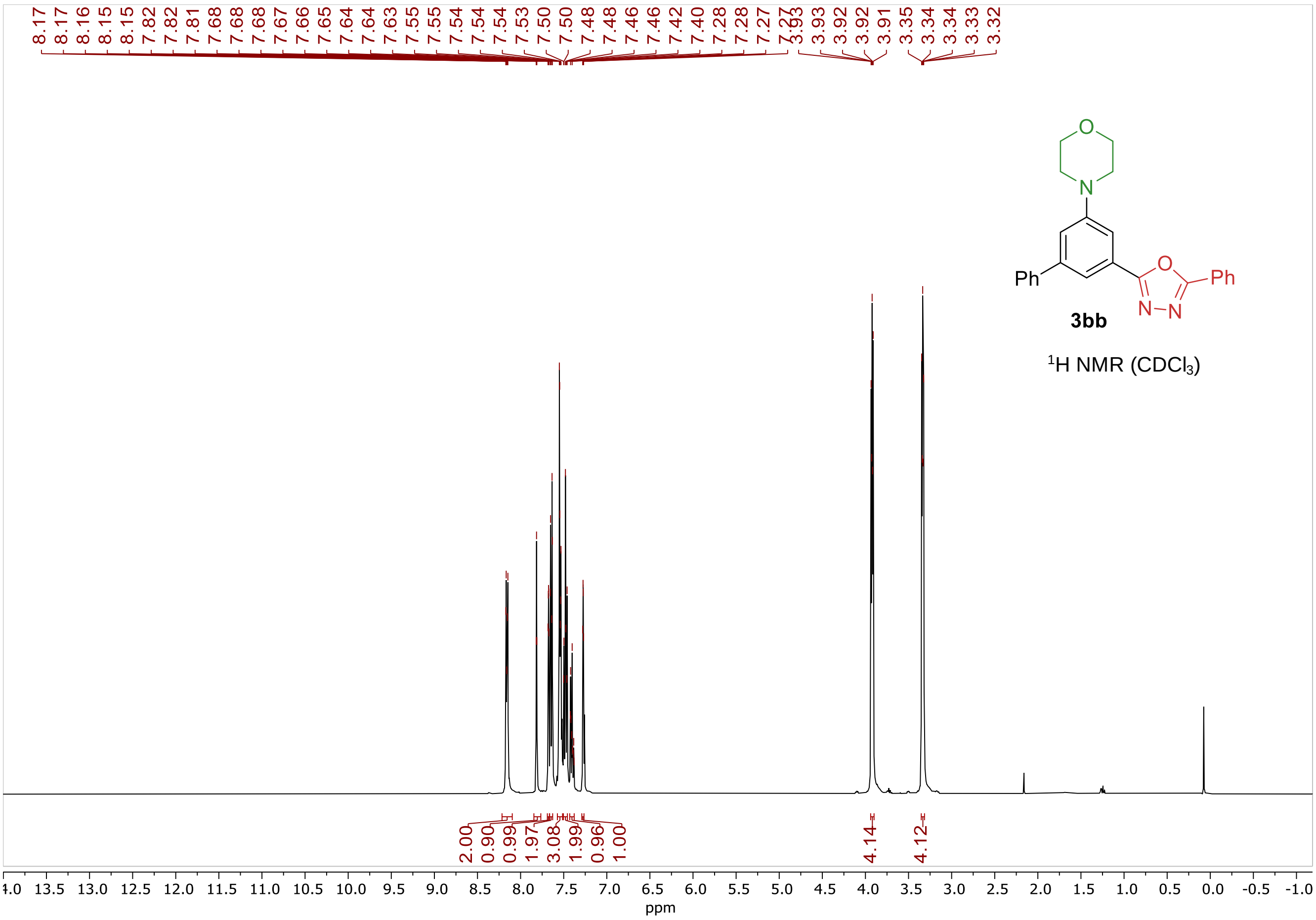
8.17 8.17 8.16 8.15 8.15 7.82 7.82 7.81 7.68 7.68 7.68 7.67 7.66 7.65 7.64 7.64 7.63 7.55 7.55 7.54 7.54 7.54 7.53 7.50 7.50 7.48 7.48 7.46 7.46 7.42 7.40 7.28 7.28 7.27 7.27
3.93 3.93 3.92 3.92 3.91 3.35 3.34 3.34 3.33 3.32
O
N
Ph
O
Ph
N–N
3bb
^{1}H NMR ($CDCl_3$)
2.00 0.90 0.99 1.97 3.08 1.99 0.96 1.00
4.14
4.12
ppm

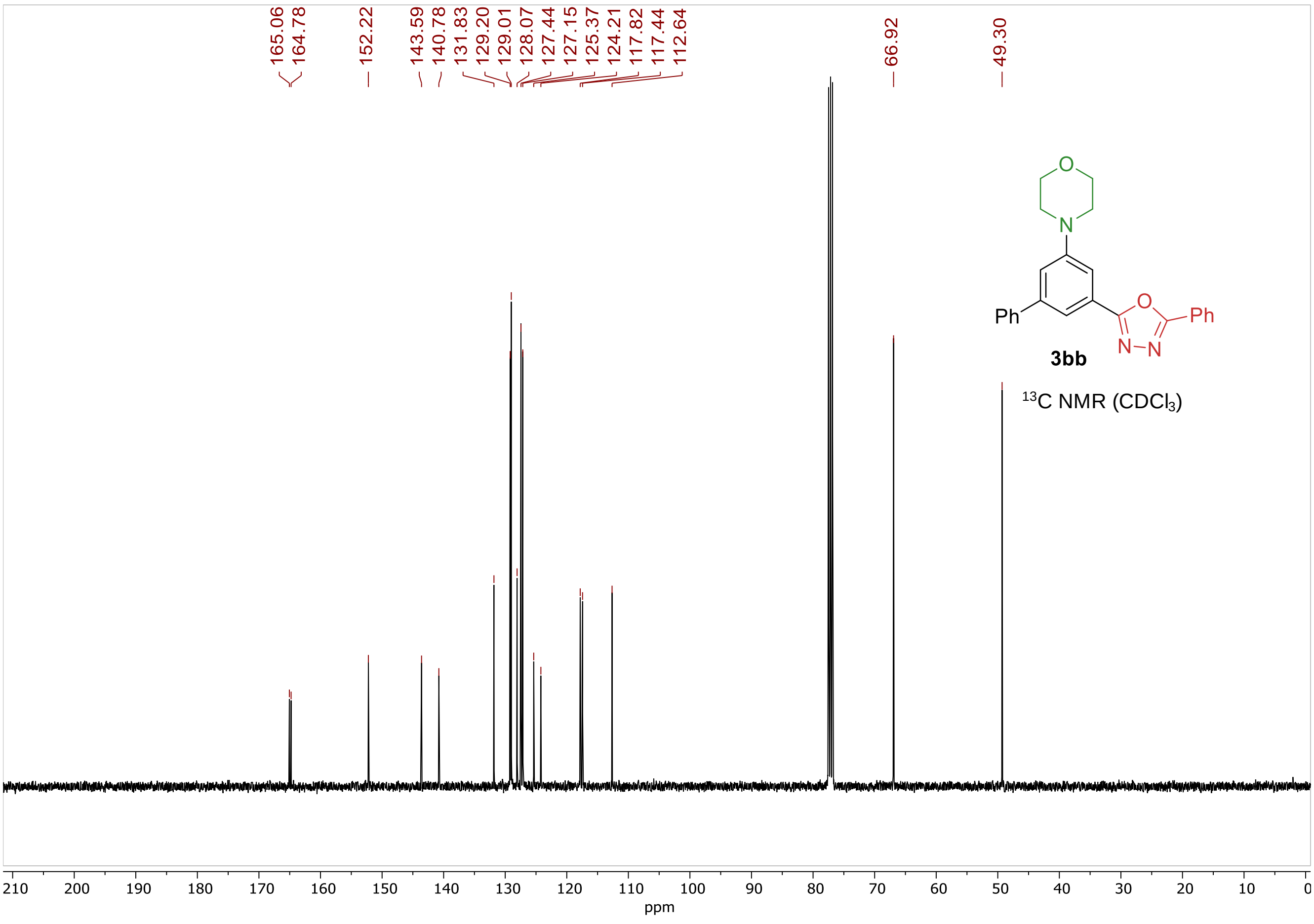
165.06 164.78
152.22
143.59 140.78 131.83 129.20 129.01 128.07 127.44 127.15 125.37 124.21 117.82 117.44 112.64
66.92
49.30
O
N
Ph
O
Ph
N–N
3bb
^{13}C NMR ($CDCl_3$)
ppm

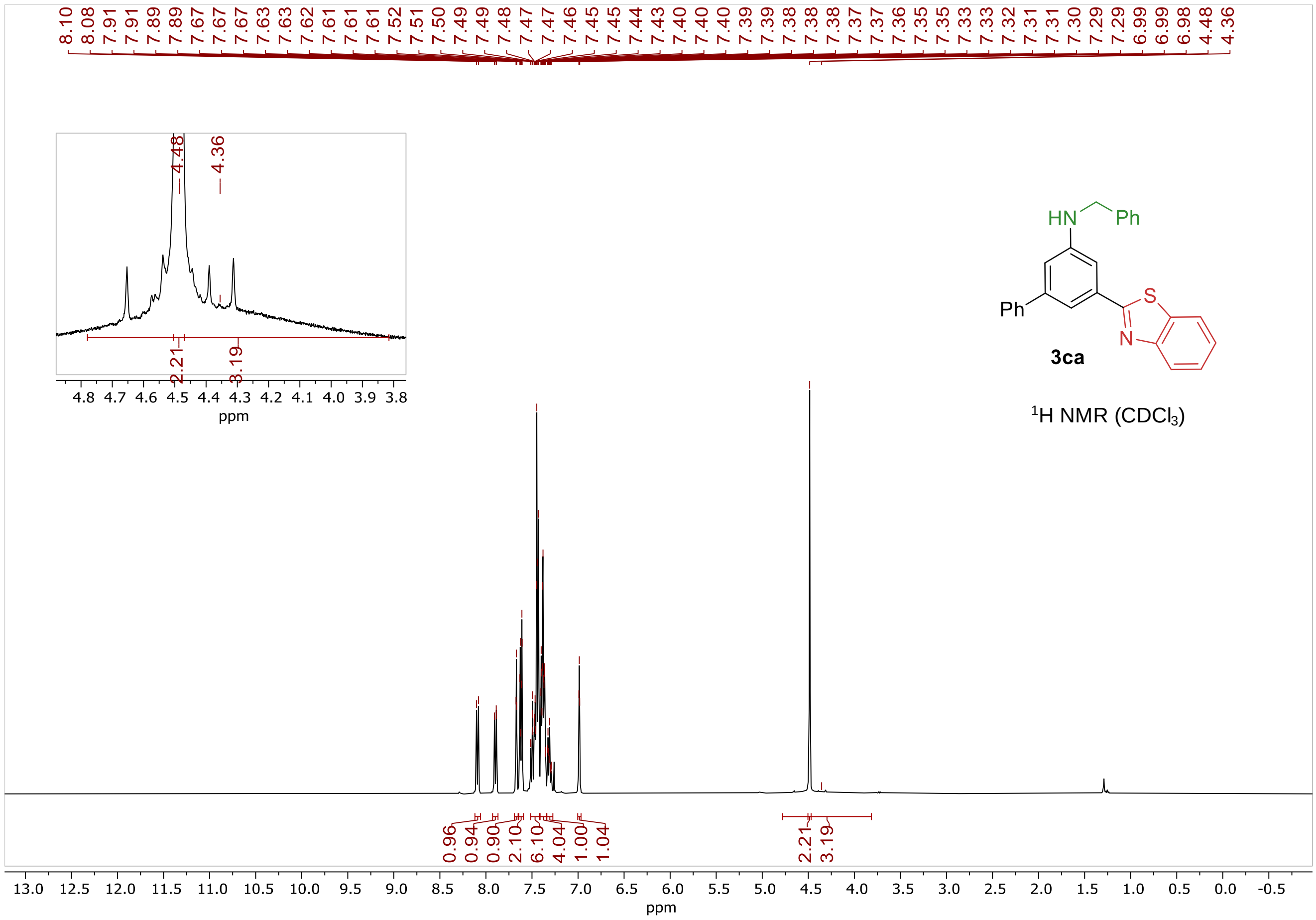
8.10
8.08
7.91
7.91
7.89
7.89
7.67
7.67
7.67
7.63
7.63
7.62
7.61
7.61
7.61
7.61
7.52
7.51
7.50
7.49
7.49
7.48
7.47
7.47
7.46
7.45
7.45
7.44
7.43
7.40
7.40
7.39
7.39
7.38
7.38
7.38
7.37
7.37
7.36
7.35
7.35
7.33
7.33
7.32
7.31
7.31
7.30
7.29
7.29
6.99
6.99
6.98
4.48
4.36
HN
Ph
Ph
S
N
3ca
^{1}H NMR (CDCl$_3$)
0.96
0.94
0.90
2.10
6.10
4.04
1.00
1.04
2.21
3.19
ppm

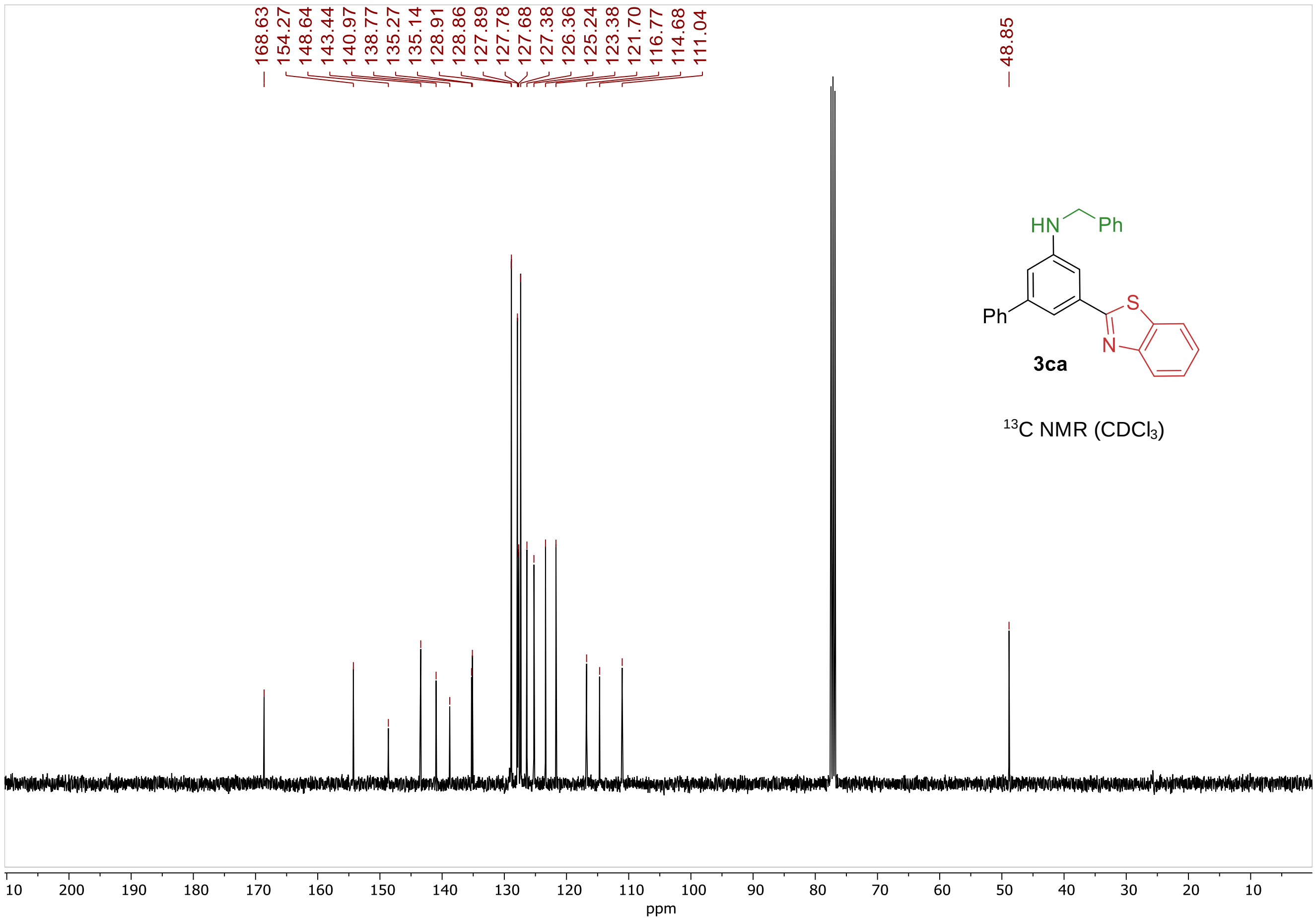
168.63
154.27
148.64
143.44
140.97
138.77
135.27
135.14
128.91
128.86
127.89
127.78
127.68
127.38
126.36
125.24
123.38
121.70
116.77
114.68
111.04
48.85
HN
Ph
Ph
S
N
3ca
^{13}C NMR (CDCl$_3$)
ppm

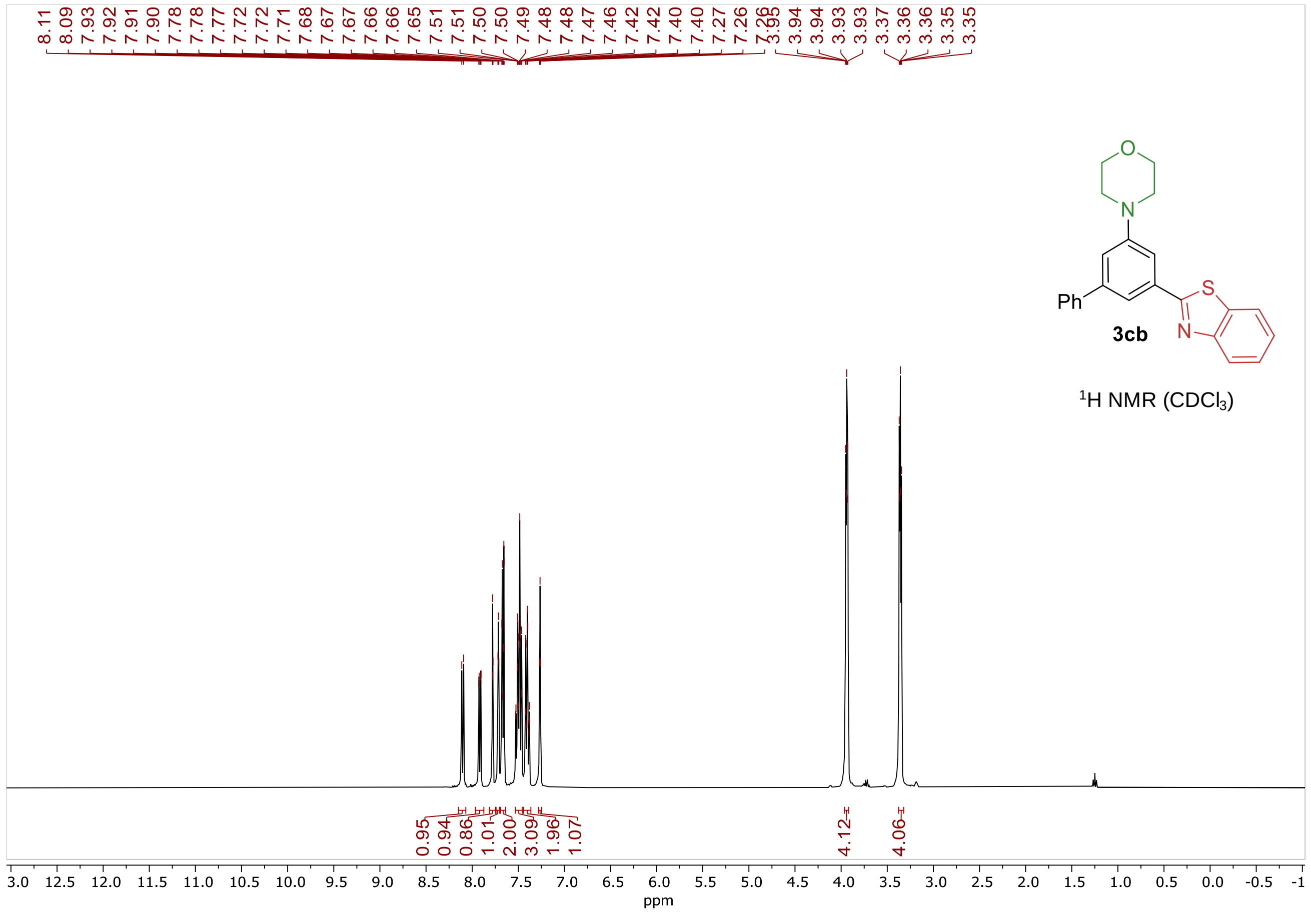
3cb
Ph
O
N
S
N
^{1}H NMR ($CDCl_3$)
ppm

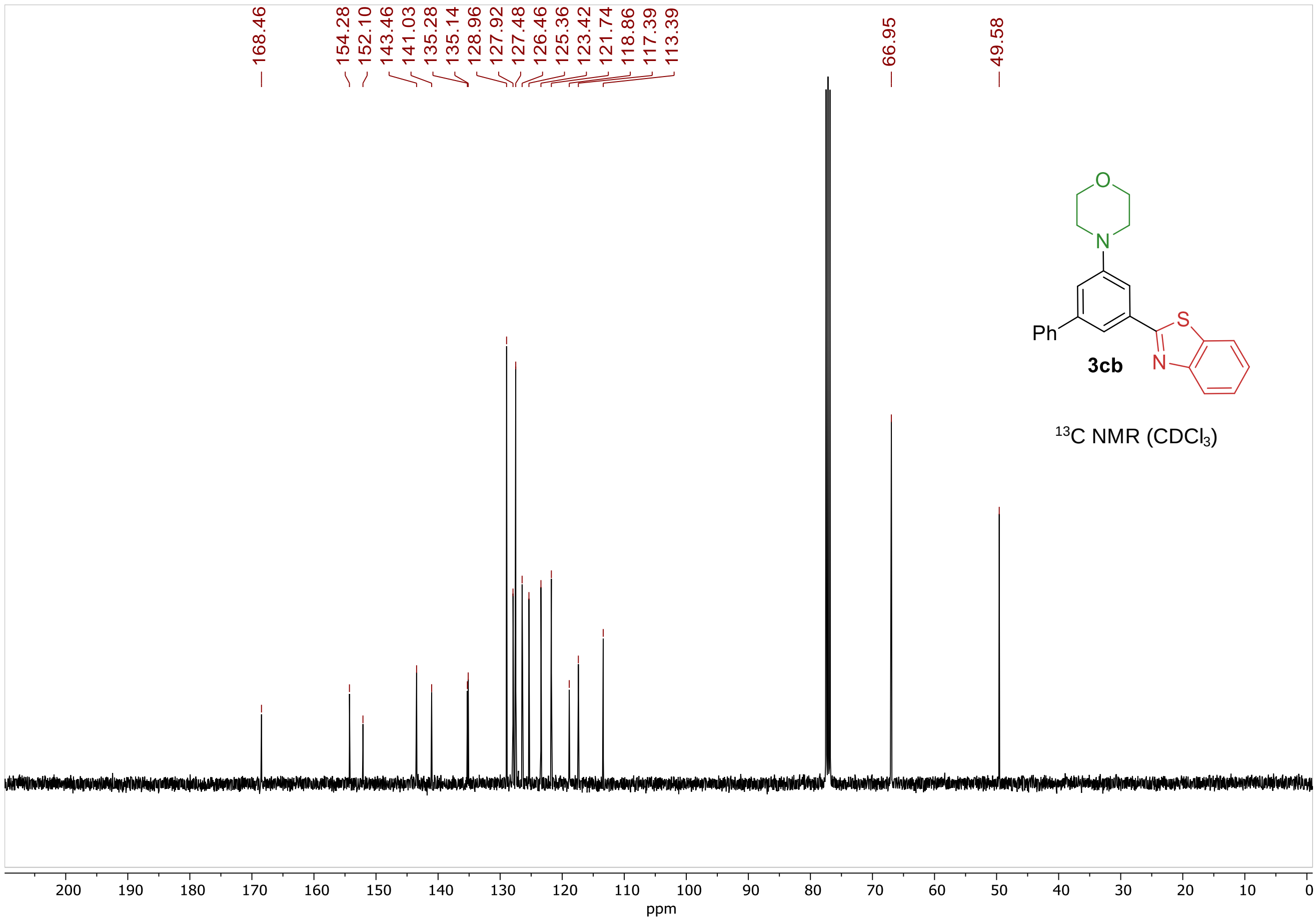
168.46
154.28
152.10
143.46
141.03
135.28
135.14
128.96
127.92
127.48
126.46
125.36
123.42
121.74
118.86
117.39
113.39
66.95
49.58
3cb
Ph
^{13}C NMR ($CDCl_3$)
ppm

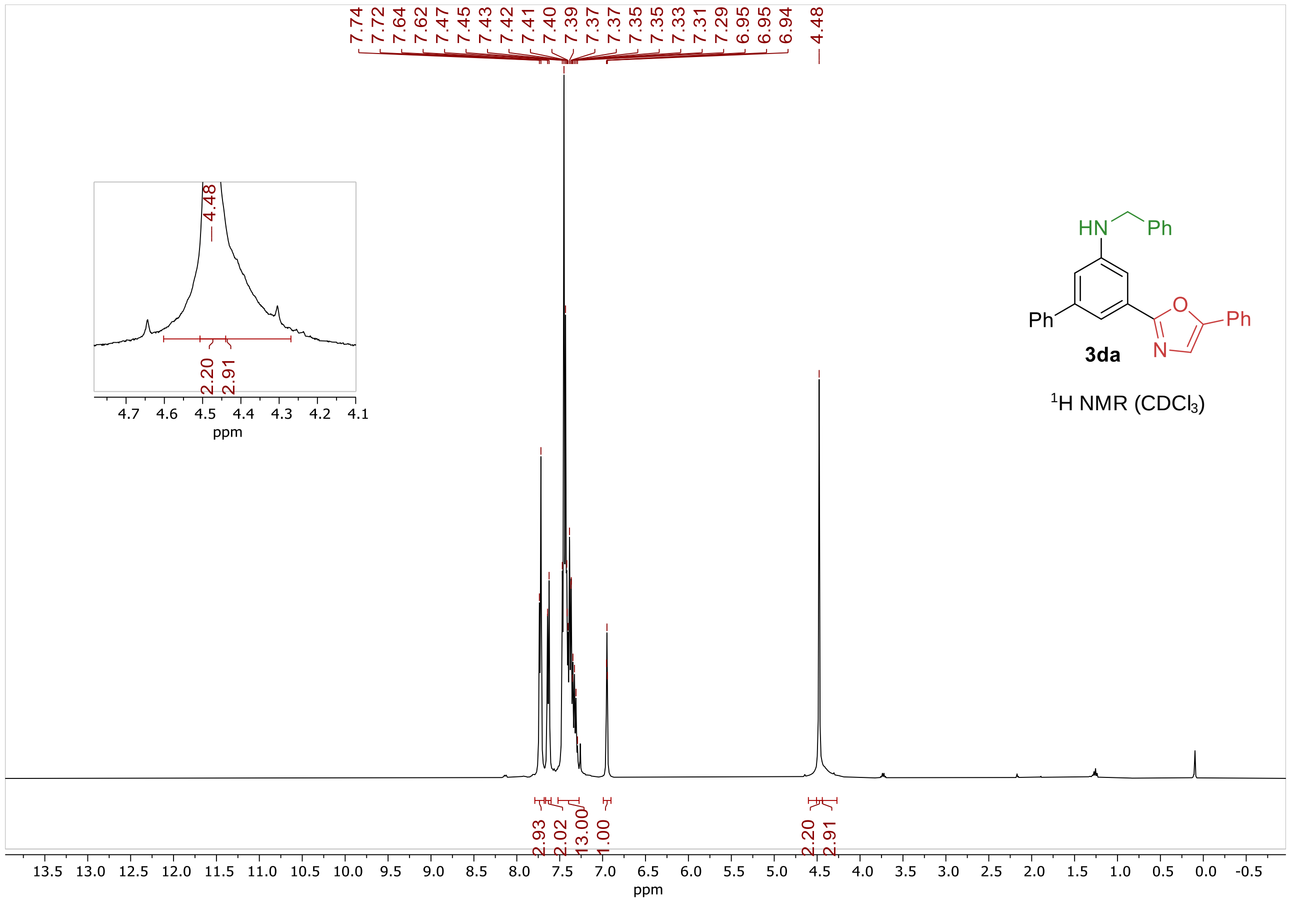
7.74 7.72 7.64 7.62 7.47 7.45 7.43 7.42 7.41 7.40 7.39 7.37 7.37 7.35 7.35 7.33 7.31 7.29 6.95 6.95 6.94
4.48
2.93 2.02 13.00 1.00 2.20 2.91
HN Ph
Ph
O
N
Ph
3da
^{1}H NMR ($CDCl_3$)
ppm

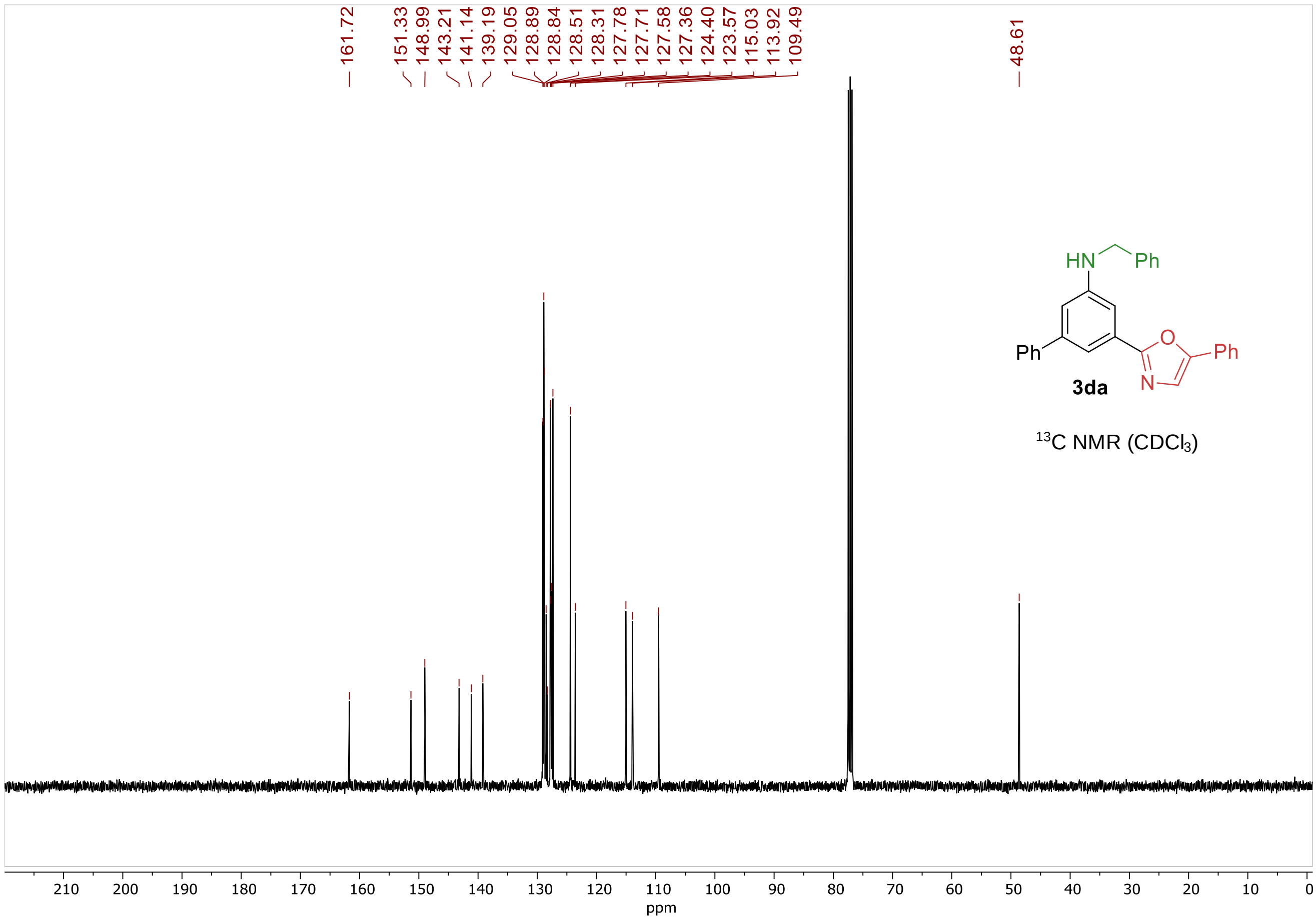
161.72
151.33 148.99 143.21 141.14 139.19 129.05 128.89 128.84 128.51 128.31 127.78 127.71 127.58 127.36 124.40 123.57 115.03 113.92 109.49
48.61
HN Ph
Ph
O
N
Ph
3da
^{13}C NMR ($CDCl_3$)
ppm

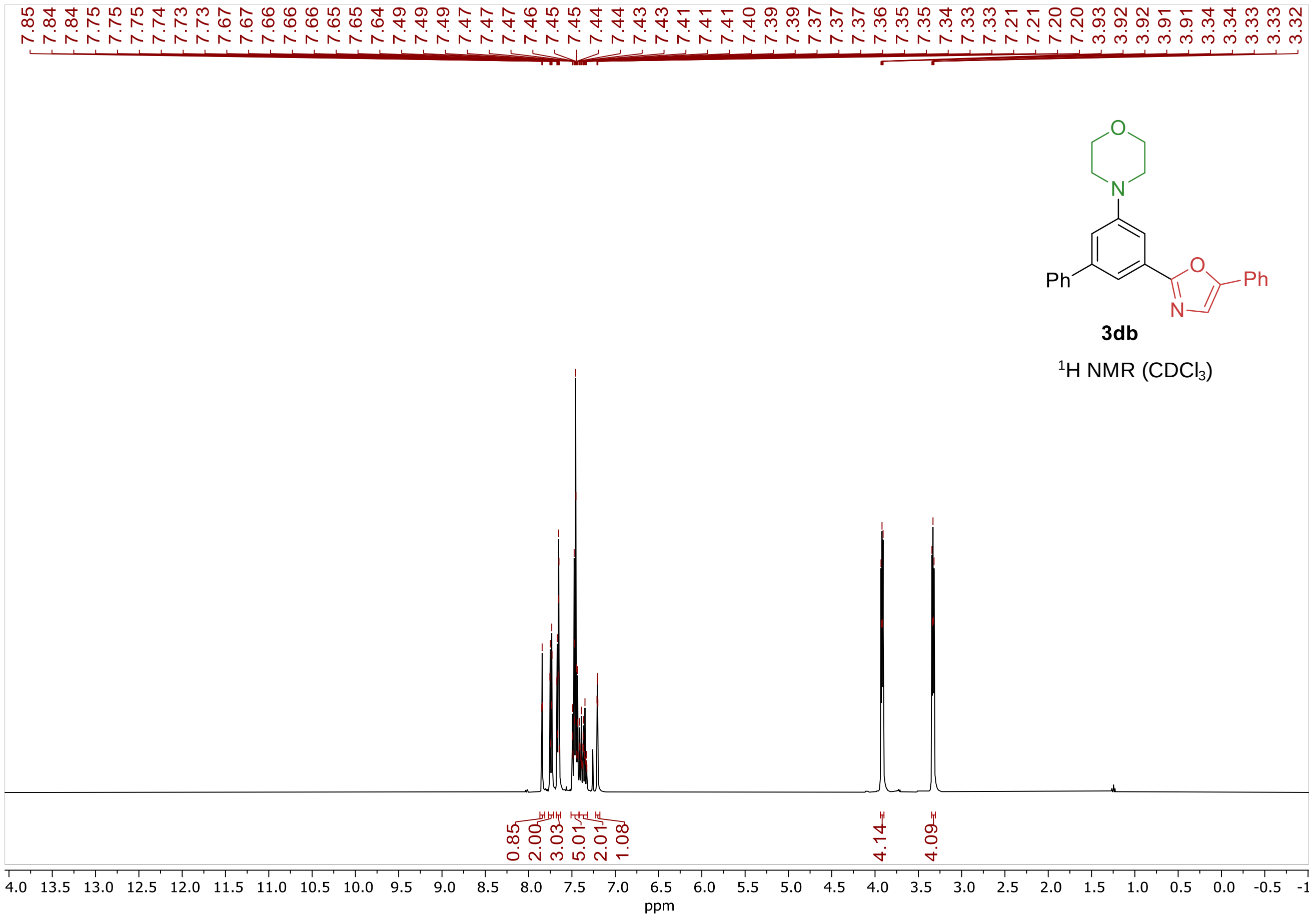
7.85
7.84
7.84
7.75
7.75
7.74
7.73
7.73
7.67
7.66
7.66
7.66
7.65
7.65
7.64
7.49
7.49
7.49
7.47
7.47
7.47
7.46
7.45
7.45
7.44
7.44
7.43
7.43
7.41
7.41
7.41
7.40
7.39
7.39
7.37
7.37
7.37
7.36
7.35
7.35
7.34
7.33
7.33
7.21
7.21
7.20
7.20
3.93
3.92
3.92
3.91
3.91
3.34
3.34
3.33
3.33
3.32
O
N
Ph
O
Ph
N
3db
1H NMR (CDCl3)
0.85
2.00
3.03
5.01
2.01
1.08
4.14
4.09
ppm

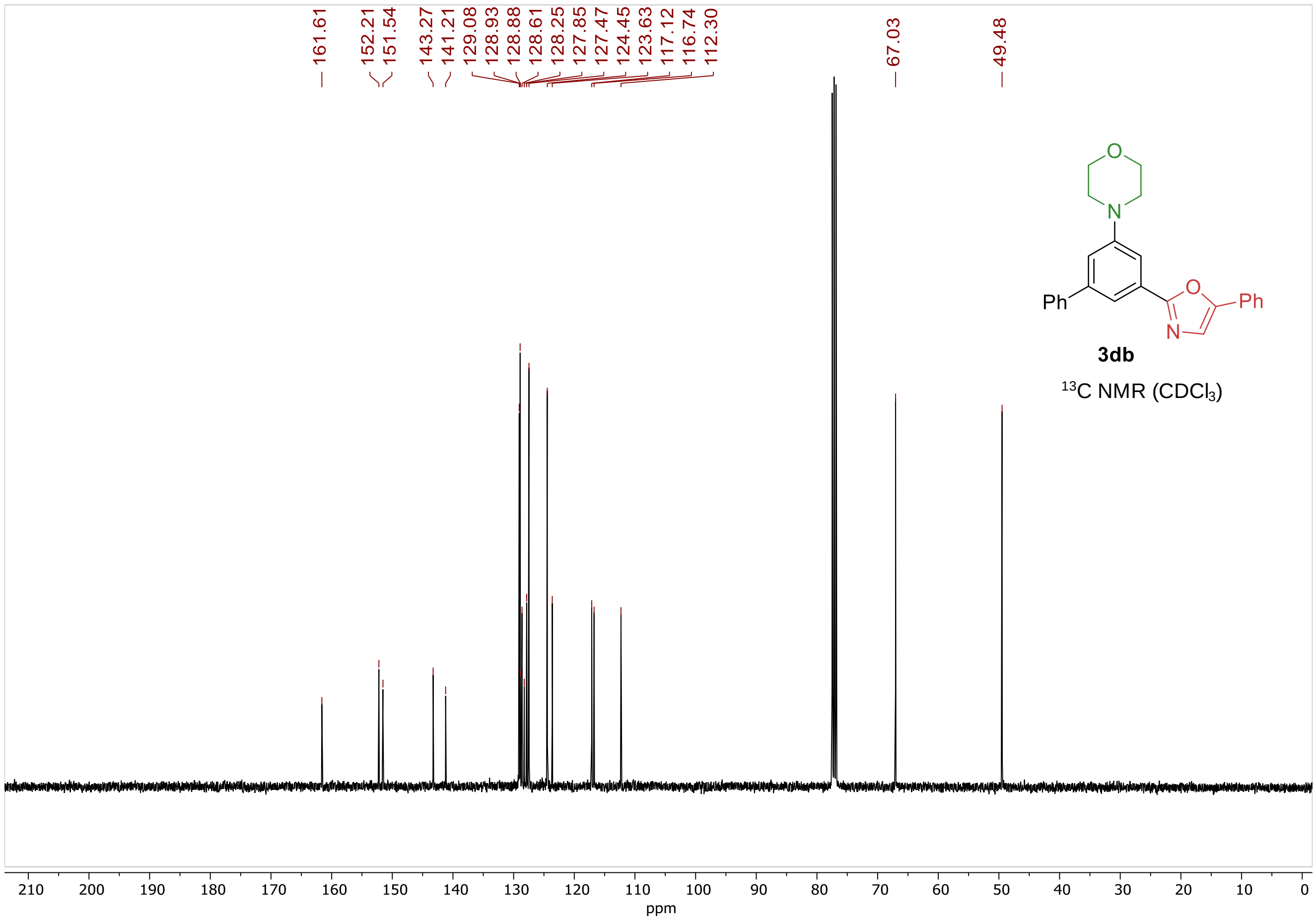
161.61
152.21
151.54
143.27
141.21
129.08
128.93
128.88
128.61
128.25
127.85
127.47
124.45
123.63
117.12
116.74
112.30
67.03
49.48
O
N
Ph
O
Ph
N
3db
13C NMR (CDCl3)
ppm

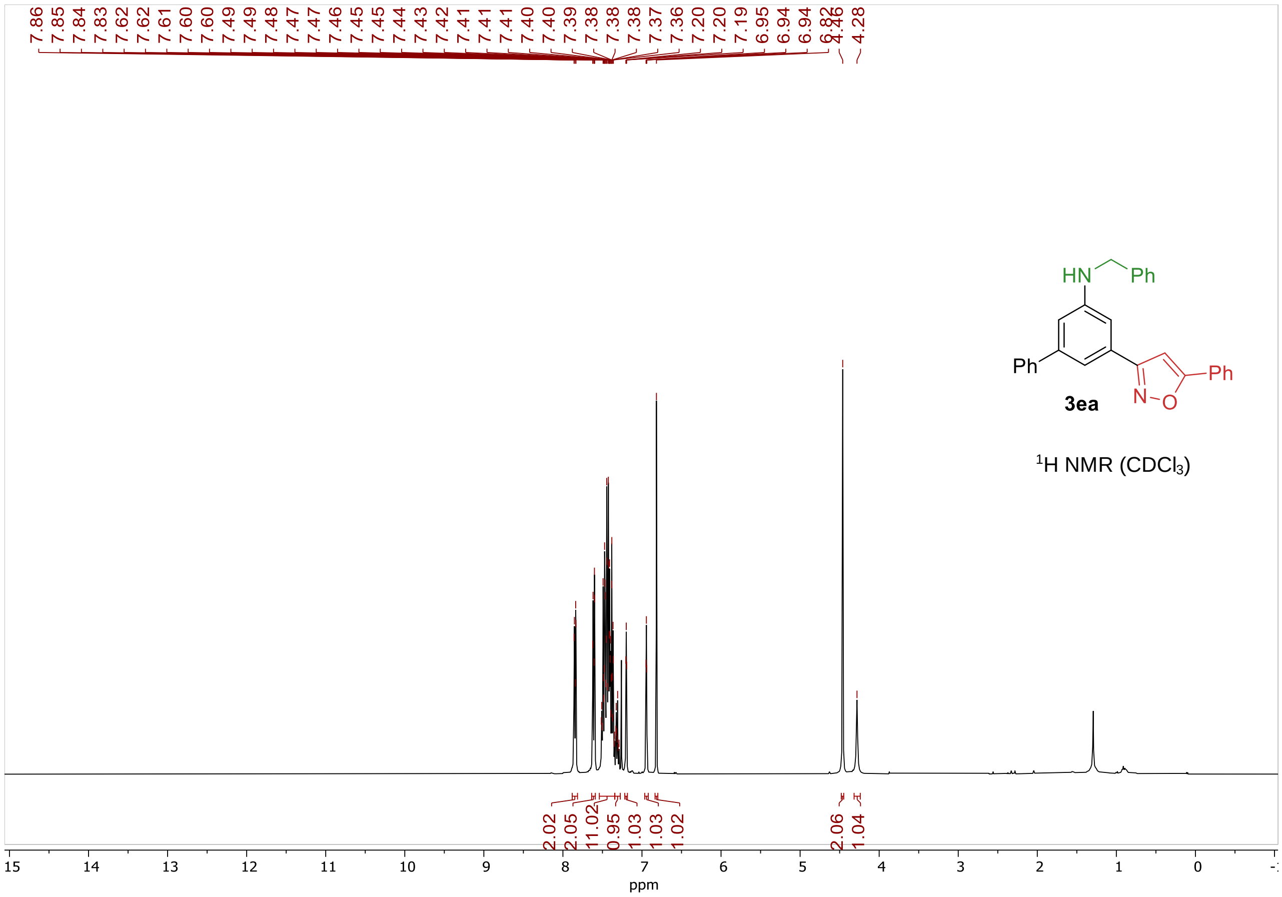
7.86 7.85 7.84 7.83 7.62 7.61 7.60 7.60 7.49 7.49 7.48 7.47 7.47 7.46 7.45 7.45 7.44 7.43 7.42 7.41 7.41 7.41 7.40 7.40 7.39 7.38 7.38 7.38 7.37 7.36 7.20 7.20 7.19 6.95 6.94 6.94 6.82 4.46 4.28
HN
Ph
Ph
Ph
N
O
3ea
^{1}H NMR ($CDCl_3$)
2.02 2.05 11.02 0.95 1.03 1.03 1.02 2.06 1.04
15 14 13 12 11 10 9 8 7 6 5 4 3 2 1 0
ppm

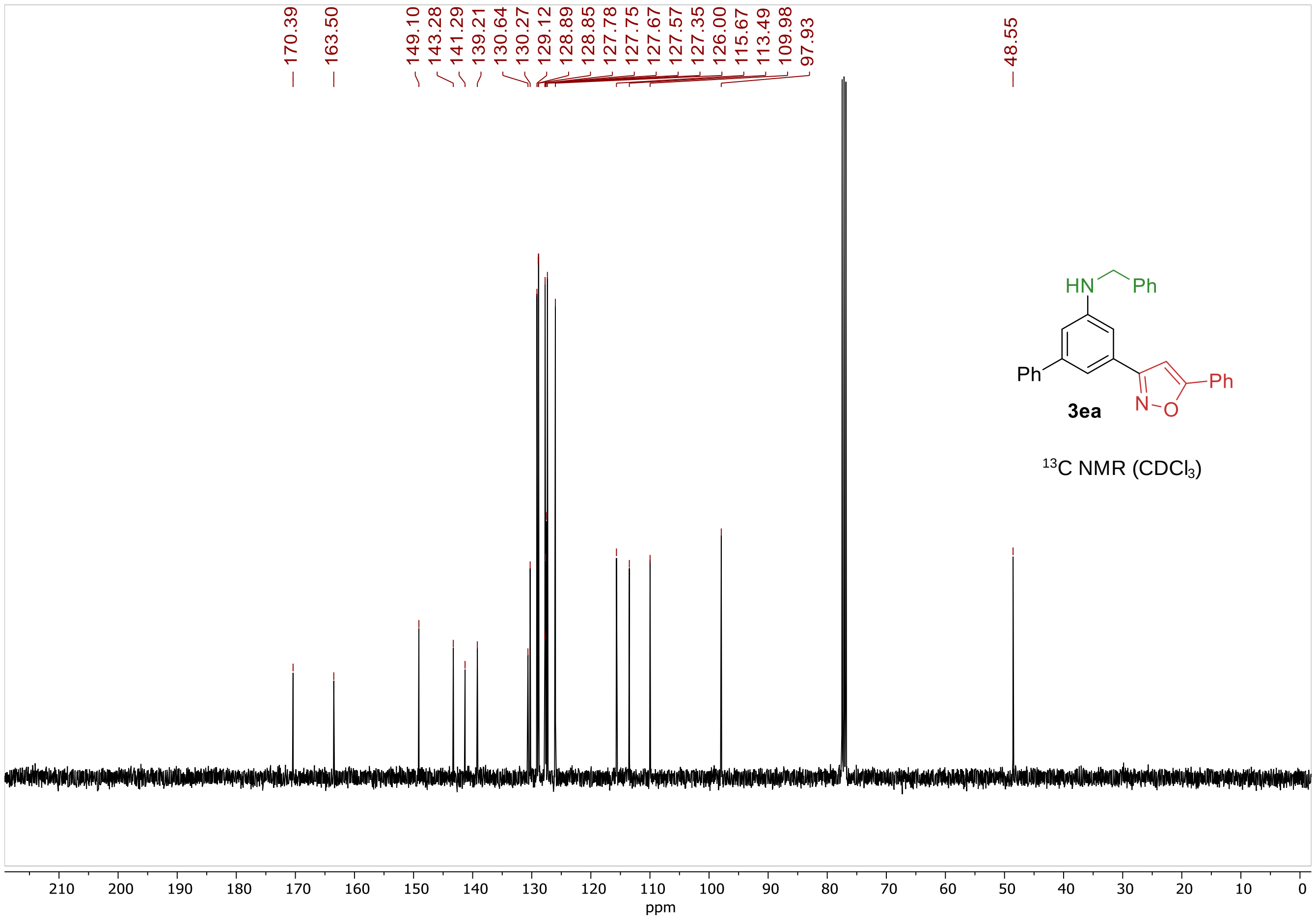
170.39 163.50 149.10 143.28 141.29 139.21 130.64 130.27 129.12 128.89 128.85 127.78 127.75 127.67 127.57 127.35 126.00 115.67 113.49 109.98 97.93 48.55
HN
Ph
Ph
Ph
N
O
3ea
^{13}C NMR ($CDCl_3$)
210 200 190 180 170 160 150 140 130 120 110 100 90 80 70 60 50 40 30 20 10 0
ppm

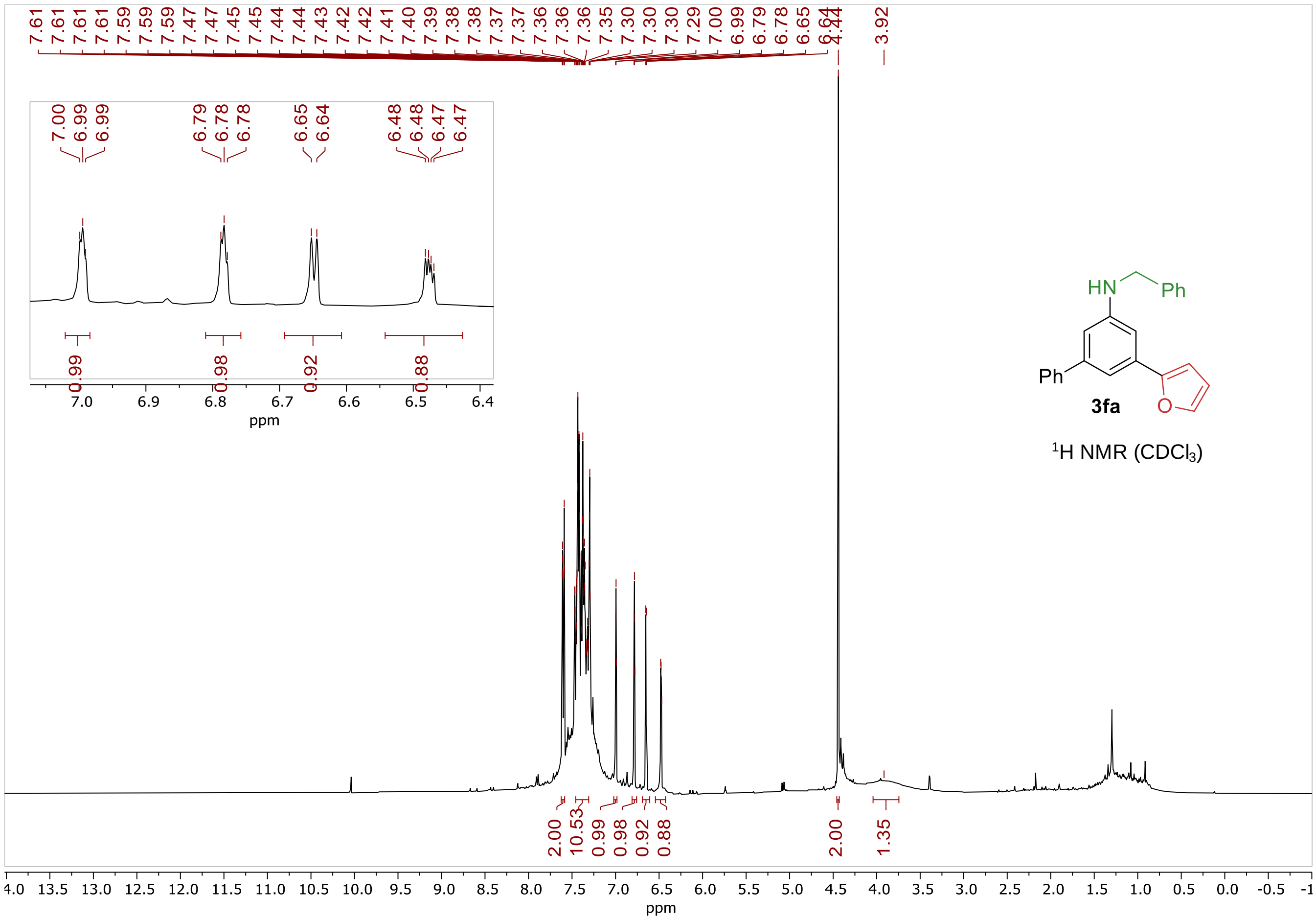
7.61 7.61 7.61 7.61 7.59 7.59 7.59 7.47 7.47 7.45 7.45 7.44 7.44 7.43 7.42 7.42 7.41 7.40 7.39 7.38 7.38 7.37 7.37 7.36 7.36 7.36 7.35 7.30 7.30 7.30 7.29 7.00 6.99 6.79 6.78 6.65 6.64 4.44 3.92
7.00 6.99 6.99 6.79 6.78 6.78 6.65 6.64 6.48 6.48 6.47 6.47
0.99 0.98 0.92 0.88
ppm
2.00 10.53 0.99 0.98 0.92 0.88 2.00 1.35
ppm
HN
Ph
Ph
3fa
O
^{1}H NMR ($CDCl_3$)

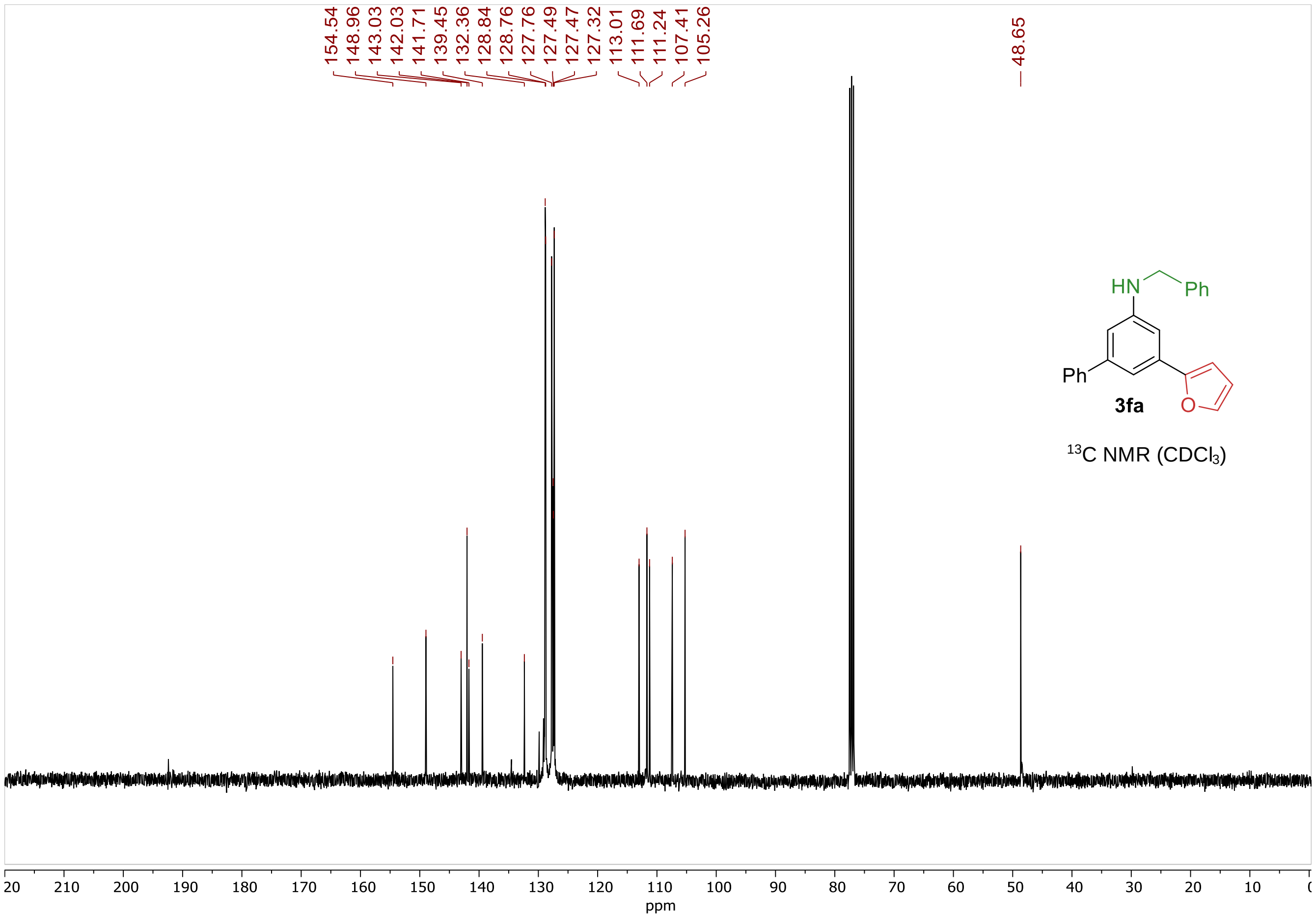
154.54 148.96 143.03 142.03 141.71 139.45 132.36 128.84 128.76 127.76 127.49 127.47 127.32 113.01 111.69 111.24 107.41 105.26 48.65
HN
Ph
Ph
3fa
O
^{13}C NMR ($CDCl_3$)
ppm

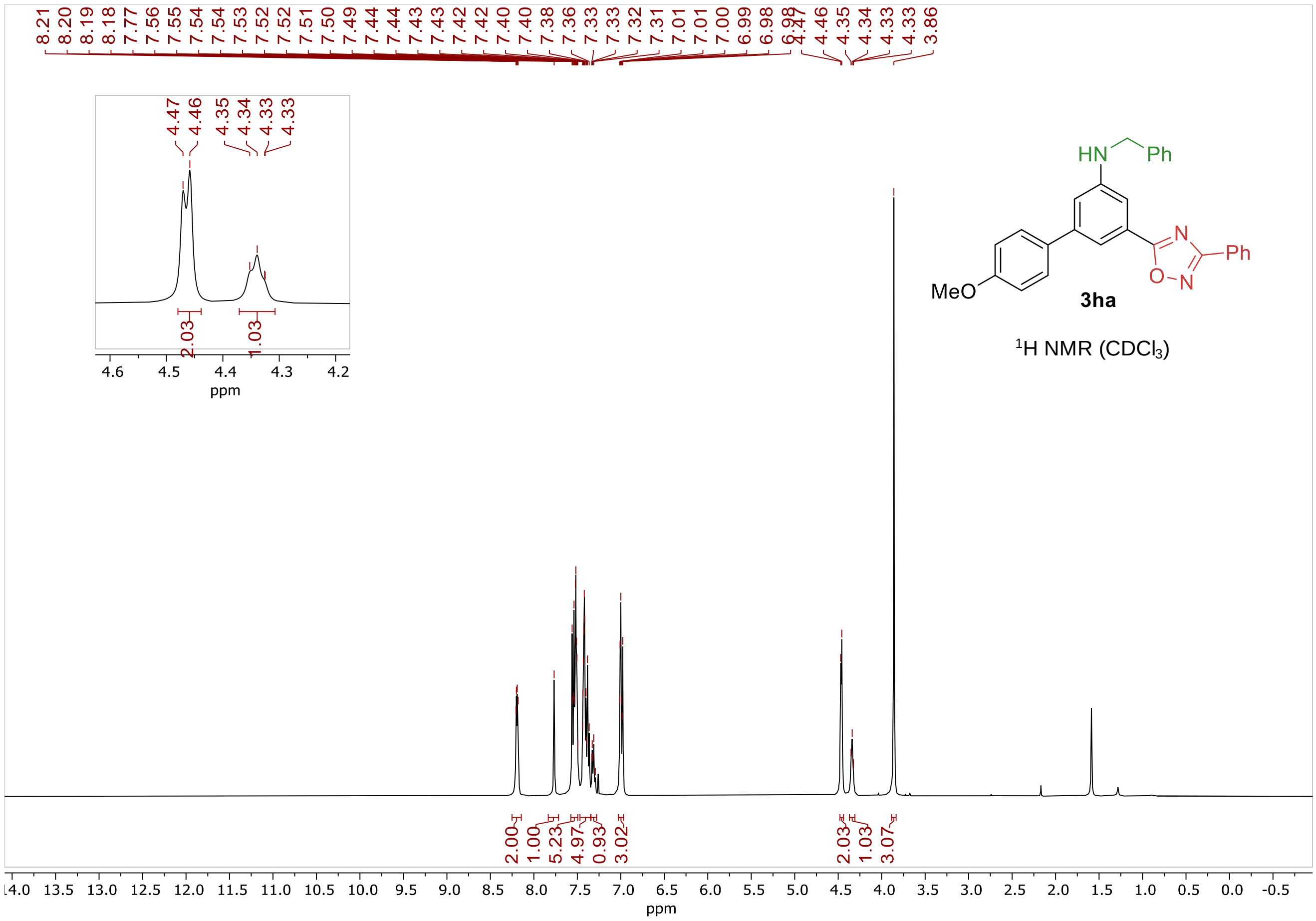
8.21 8.20 8.19 8.18 7.77 7.56 7.55 7.54 7.54 7.53 7.52 7.52 7.51 7.50 7.49 7.44 7.44 7.43 7.43 7.42 7.42 7.40 7.40 7.38 7.36 7.33 7.33 7.32 7.31 7.01 7.01 7.00 6.99 6.98 4.47 4.46 4.35 4.34 4.33 4.33 3.86
4.47 4.46 4.35 4.34 4.33 4.33
2.03 1.03
HN Ph
MeO
O N N Ph
3ha
^{1}H NMR ($CDCl_3$)
2.00 1.00 5.23 4.97 0.93 3.02 2.03 1.03 3.07
ppm

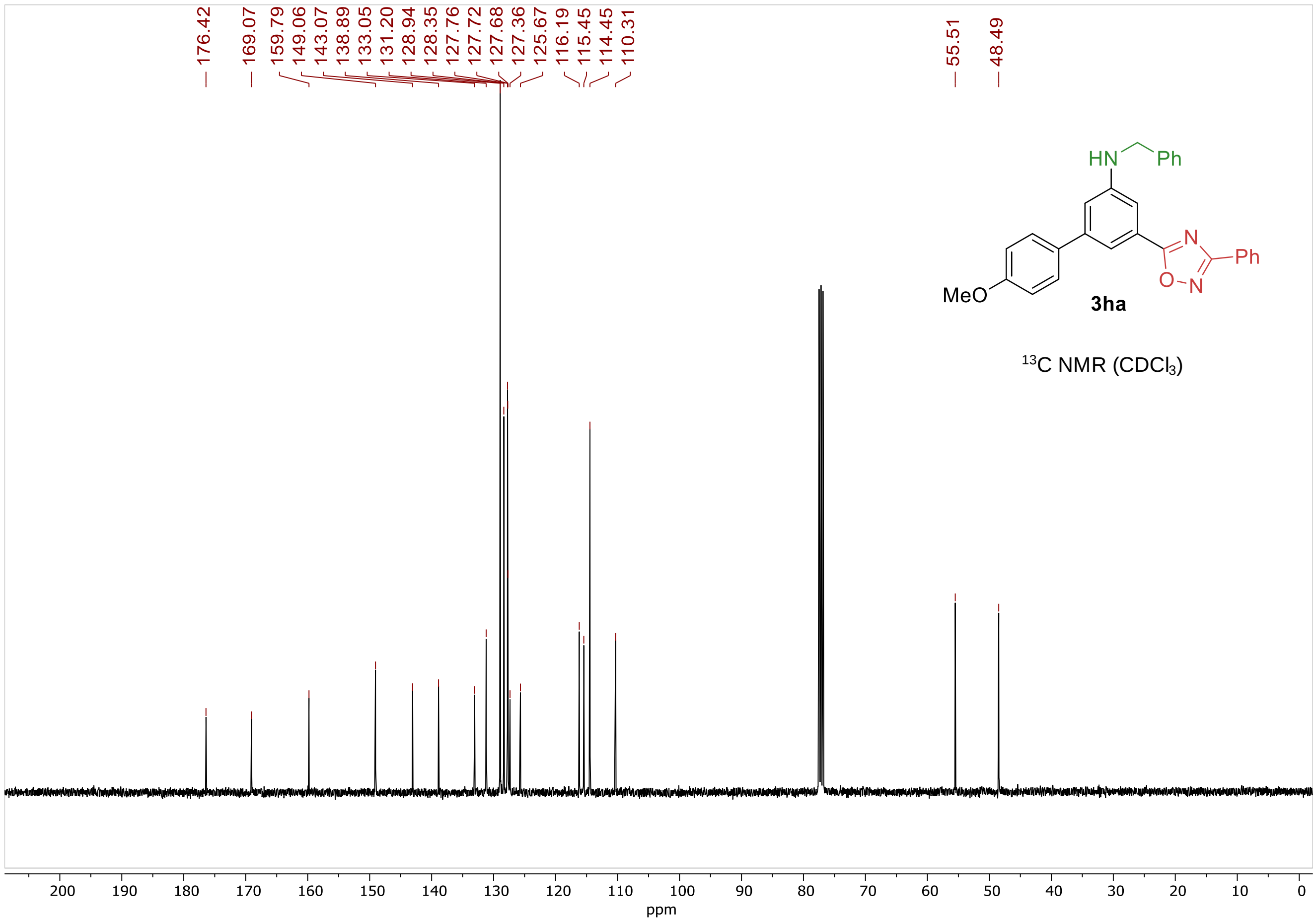
176.42 169.07 159.79 149.06 143.07 138.89 133.05 131.20 128.94 128.35 127.76 127.72 127.68 127.36 125.67 116.19 115.45 114.45 110.31 55.51 48.49
HN Ph
MeO
O N N Ph
3ha
^{13}C NMR ($CDCl_3$)
ppm

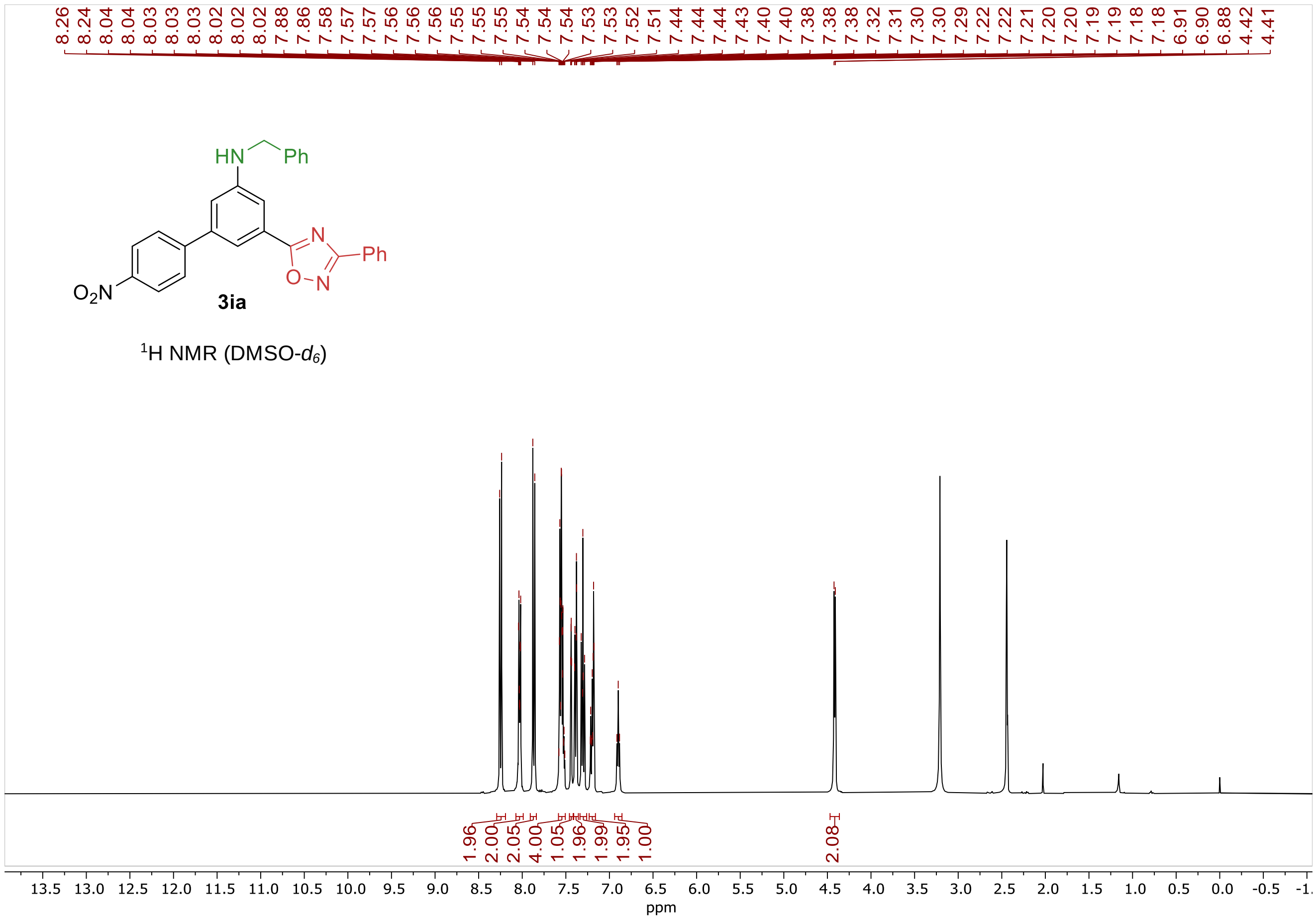
8.26 8.24 8.04 8.04 8.03 8.03 8.03 8.02 8.02 8.02 7.88 7.86 7.58 7.57 7.57 7.56 7.56 7.56 7.55 7.55 7.55 7.54 7.54 7.54 7.53 7.53 7.52 7.51 7.44 7.44 7.44 7.43 7.40 7.40 7.38 7.38 7.38 7.32 7.31 7.30 7.30 7.29 7.22 7.22 7.21 7.20 7.20 7.19 7.19 7.18 7.18 6.91 6.90 6.88 4.42 4.41
HN
Ph
N
Ph
O
N
O2N
3ia
1H NMR (DMSO-d6)
1.96 2.00 2.05 4.00 1.05 1.96 1.99 1.95 1.00
2.08
13.5 13.0 12.5 12.0 11.5 11.0 10.5 10.0 9.5 9.0 8.5 8.0 7.5 7.0 6.5 6.0 5.5 5.0 4.5 4.0 3.5 3.0 2.5 2.0 1.5 1.0 0.5 0.0 -0.5
ppm

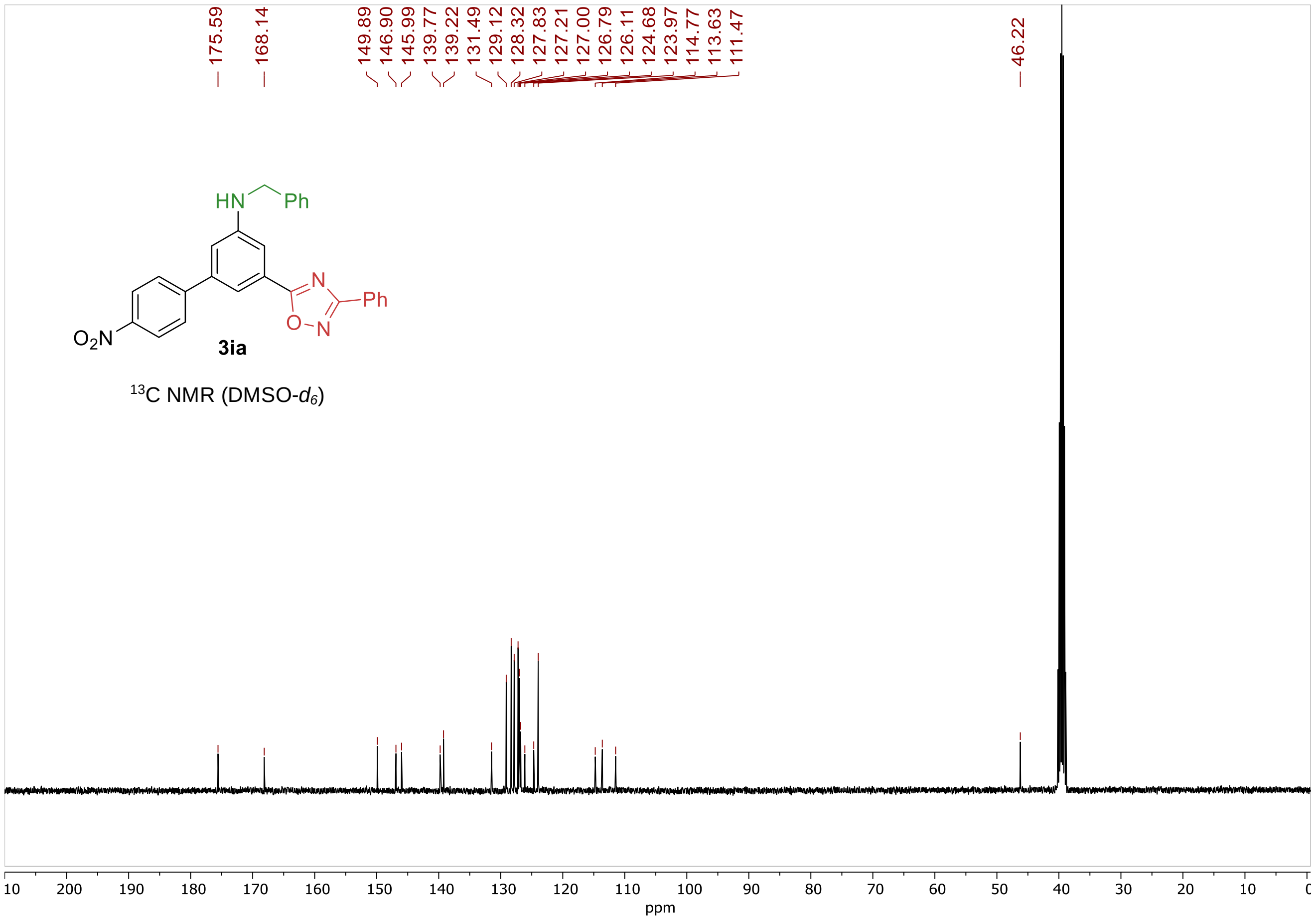
175.59
168.14
149.89 146.90 145.99 139.77 139.22 131.49 129.12 128.32 127.83 127.21 127.00 126.79 126.11 124.68 123.97 114.77 113.63 111.47
46.22
HN
Ph
N
Ph
O
N
O2N
3ia
13C NMR (DMSO-d6)
200 190 180 170 160 150 140 130 120 110 100 90 80 70 60 50 40 30 20 10
ppm

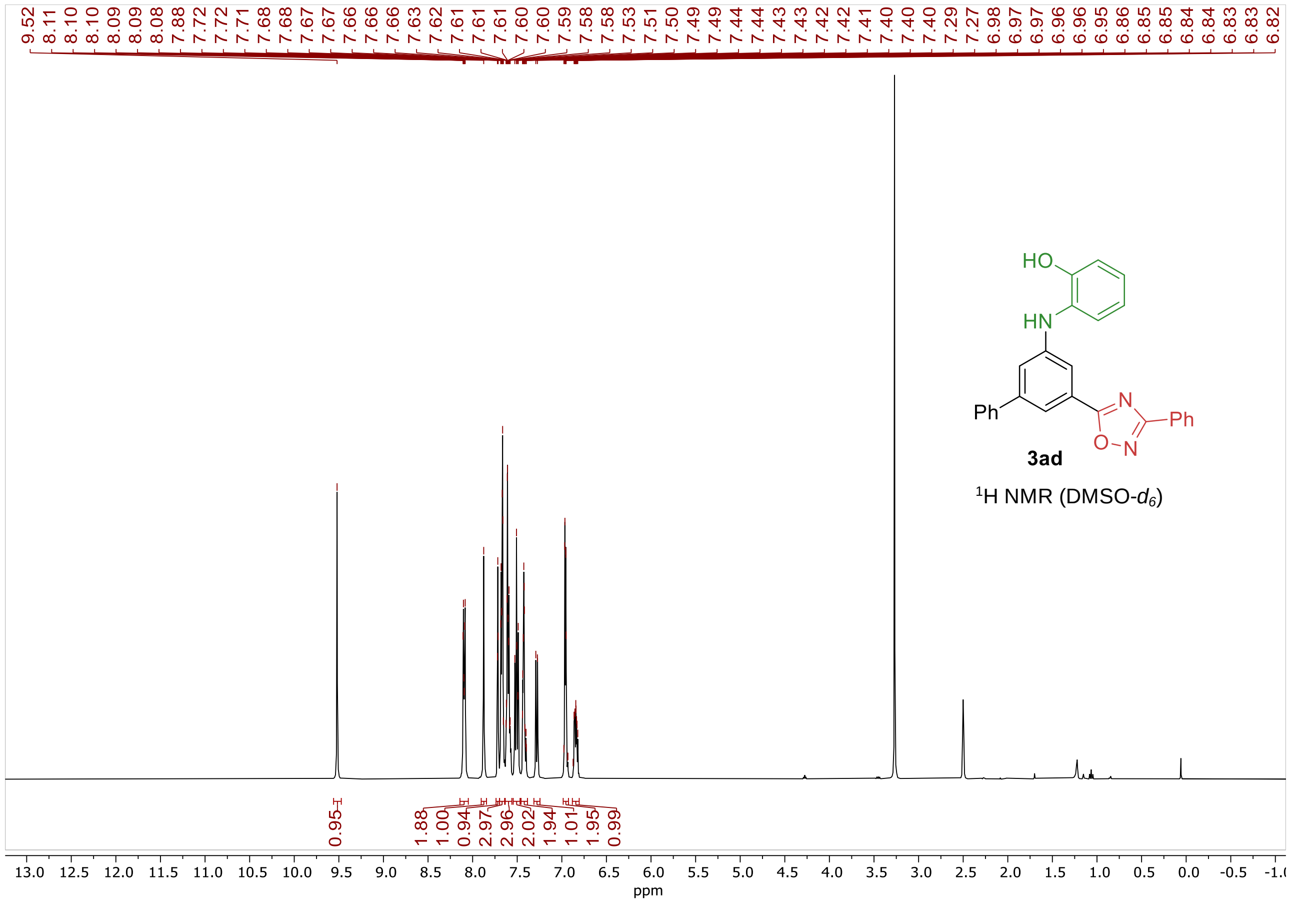
9.52 8.11 8.10 8.10 8.09 8.09 8.08 7.88 7.72 7.72 7.71 7.68 7.68 7.67 7.67 7.66 7.66 7.66 7.63 7.62 7.61 7.61 7.61 7.61 7.60 7.60 7.59 7.58 7.58 7.53 7.51 7.50 7.49 7.49 7.44 7.44 7.43 7.43 7.42 7.42 7.41 7.40 7.40 7.40 7.29 7.27 6.98 6.97 6.97 6.96 6.96 6.95 6.86 6.85 6.85 6.84 6.84 6.83 6.83 6.82
0.95 1.88 1.00 0.94 2.97 2.96 2.02 1.94 1.01 1.95 0.99
HO
HN
Ph
N
Ph
O
N
3ad
1H NMR (DMSO-d6)
ppm

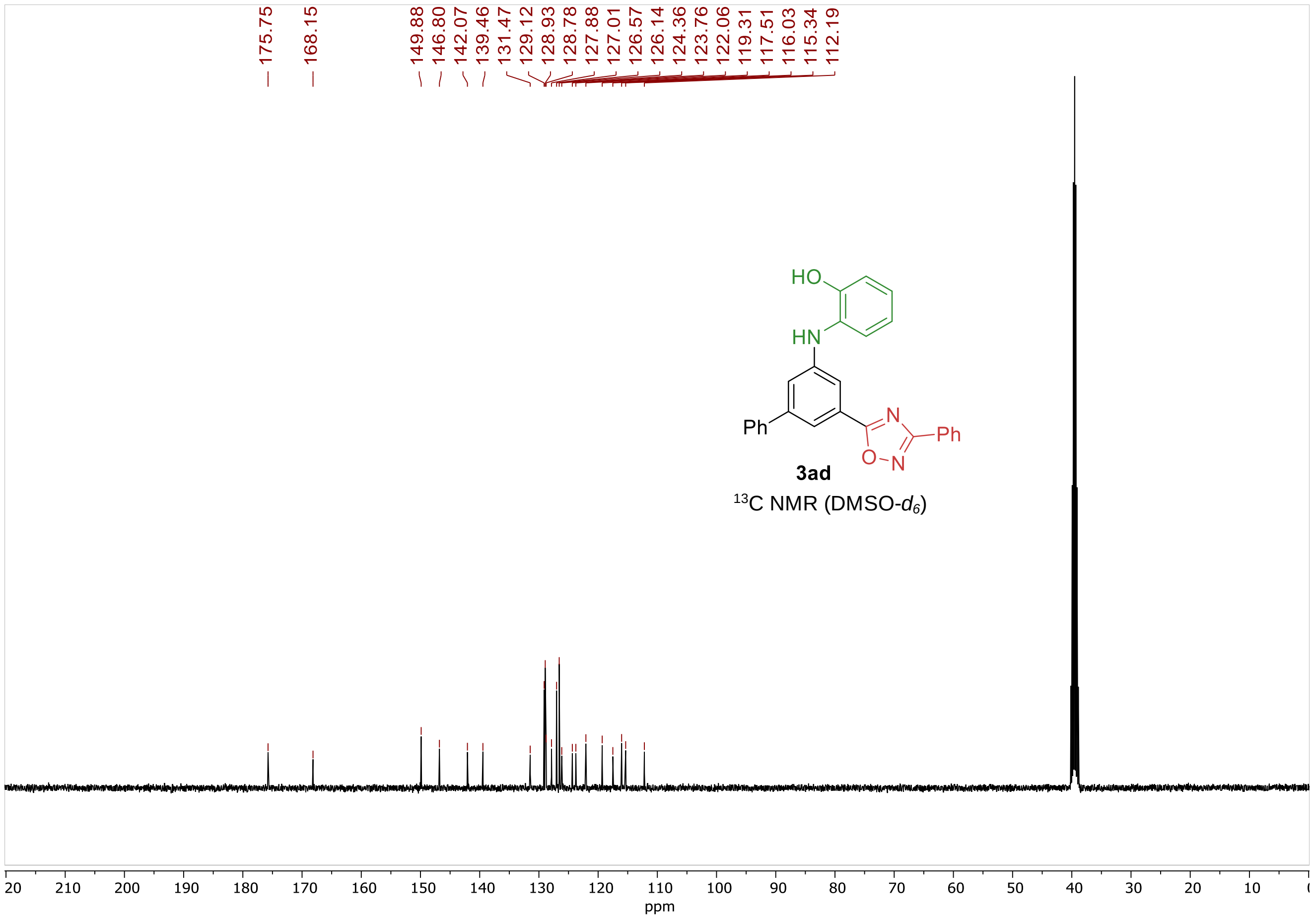
175.75 168.15 149.88 146.80 142.07 139.46 131.47 129.12 128.93 128.78 127.88 127.01 126.57 126.14 124.36 123.76 122.06 119.31 117.51 116.03 115.34 112.19
HO
HN
Ph
N
Ph
O
N
3ad
13C NMR (DMSO-d6)
ppm

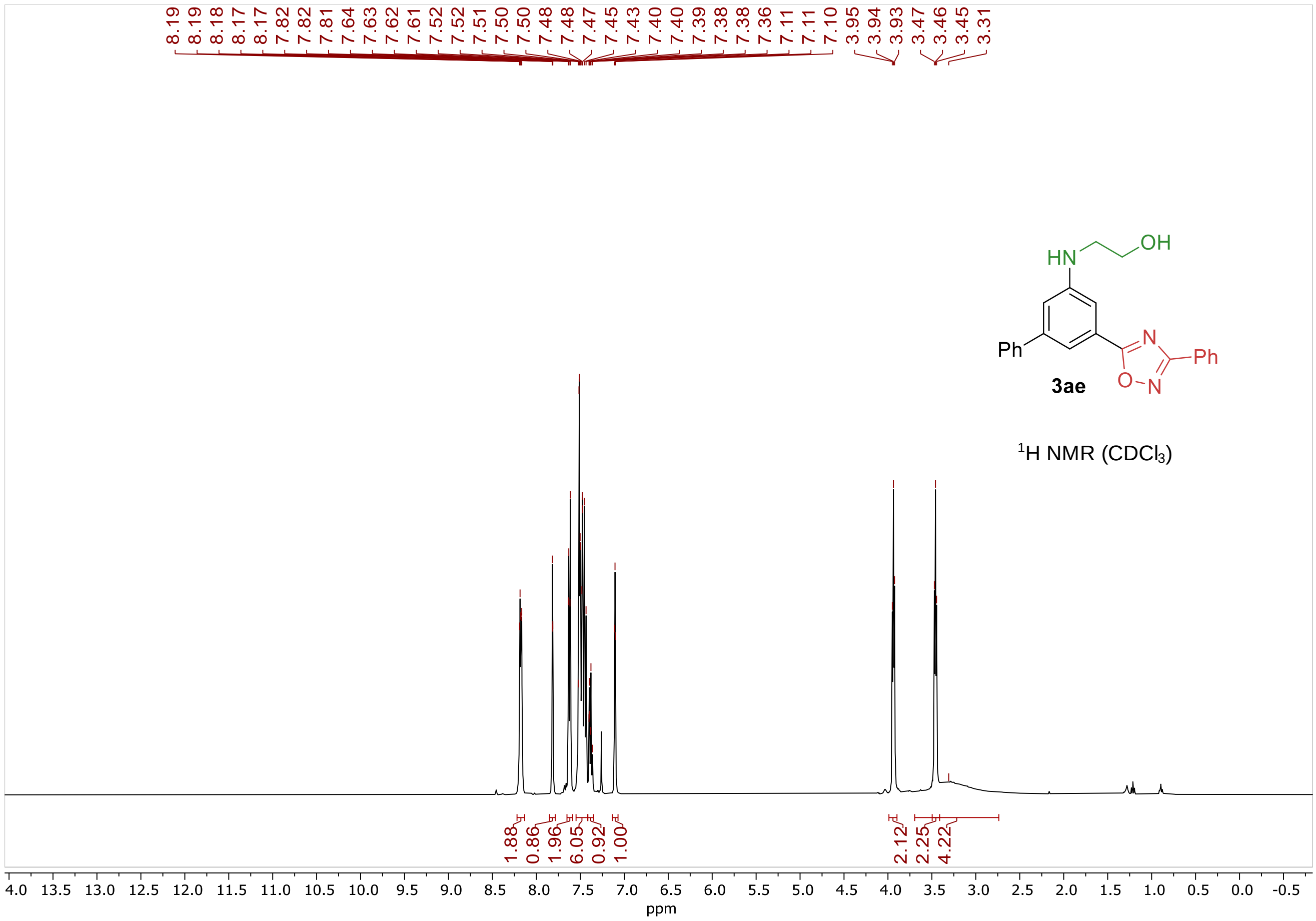

8.19
8.19
8.18
8.17
8.17
7.82
7.82
7.81
7.64
7.63
7.62
7.61
7.52
7.52
7.51
7.50
7.50
7.48
7.48
7.47
7.45
7.43
7.40
7.40
7.39
7.38
7.38
7.36
7.11
7.11
7.10
3.95
3.94
3.93
3.47
3.46
3.45
3.31
HN
OH
Ph
N
Ph
3ae
O
N
^{1}H NMR ($CDCl_3$)
1.88
0.86
1.96
6.05
0.92
1.00
2.12
2.25
4.22
ppm

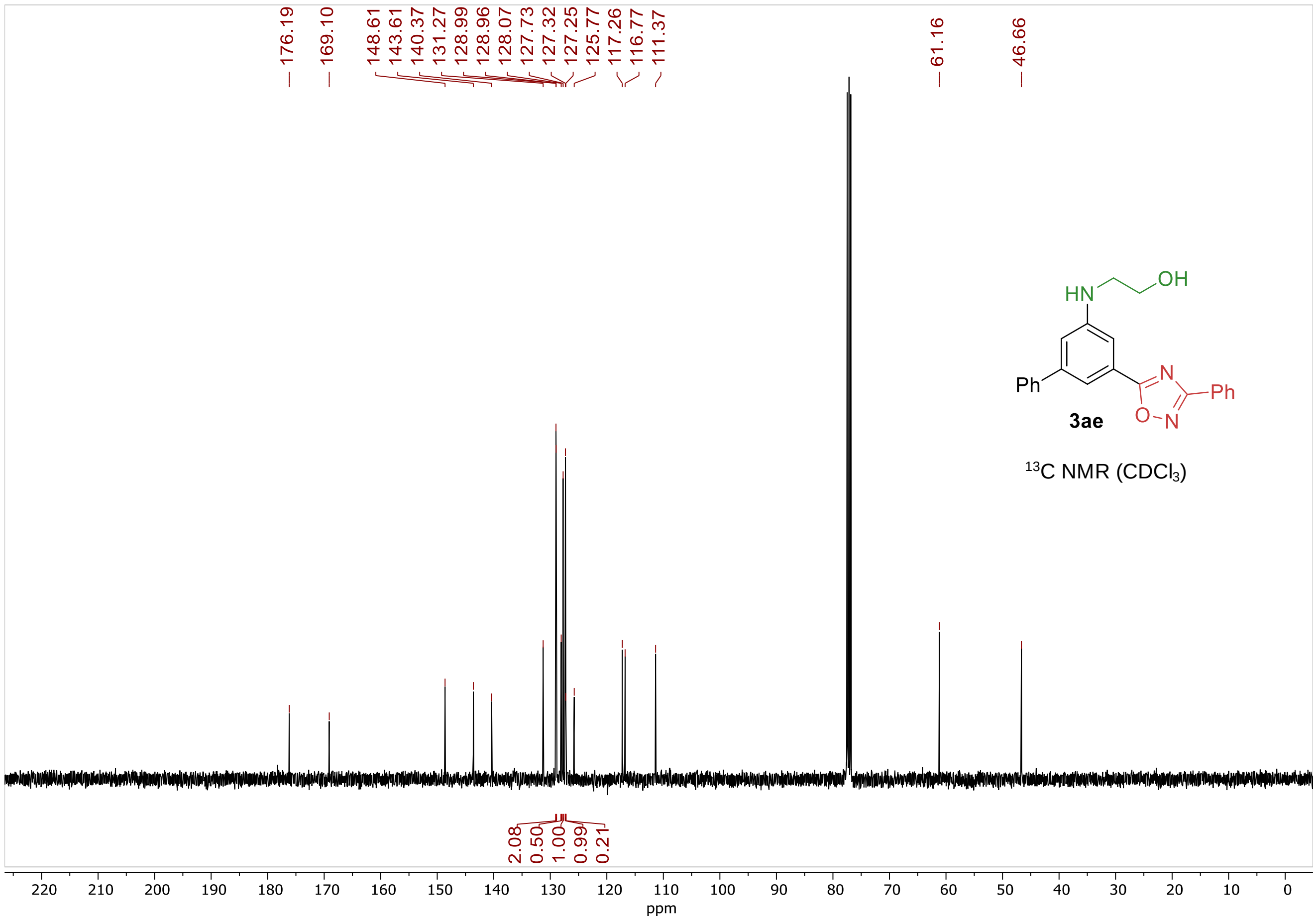

176.19
169.10
148.61
143.61
140.37
131.27
128.99
128.96
128.07
127.73
127.32
127.25
125.77
117.26
116.77
111.37
61.16
46.66
HN
OH
Ph
N
Ph
3ae
O
N
^{13}C NMR ($CDCl_3$)
2.08
0.50
1.00
0.99
0.21
ppm

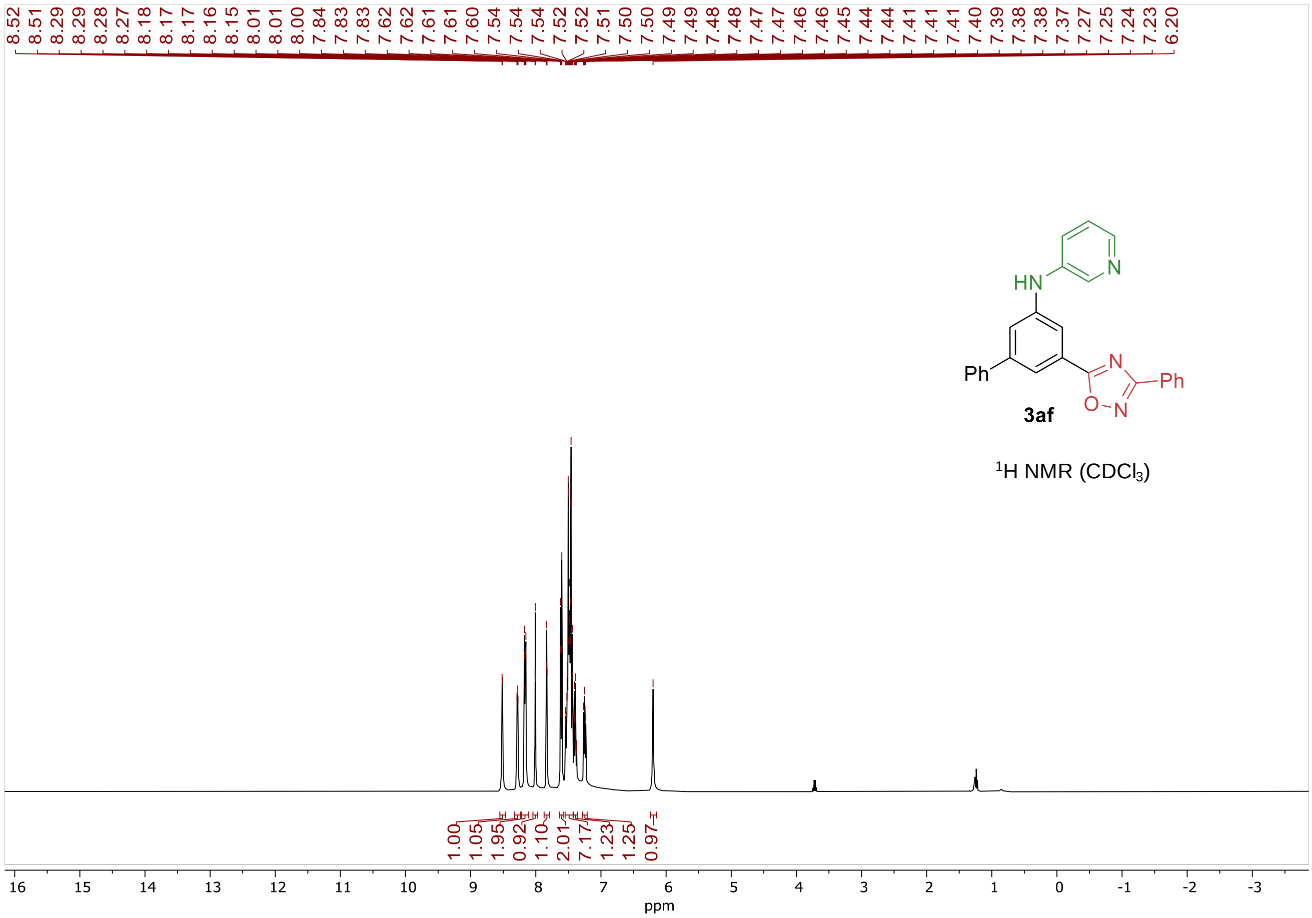
8.52
8.51
8.29
8.29
8.28
8.27
8.18
8.17
8.17
8.16
8.15
8.01
8.01
8.00
7.84
7.83
7.83
7.62
7.62
7.61
7.61
7.60
7.54
7.54
7.54
7.52
7.52
7.51
7.50
7.50
7.49
7.49
7.48
7.48
7.47
7.47
7.46
7.46
7.45
7.44
7.44
7.41
7.41
7.41
7.40
7.39
7.38
7.38
7.37
7.27
7.25
7.24
7.23
6.20
HN
N
Ph
N
Ph
O
N
3af
^{1}H NMR (CDCl3)
1.00
1.05
1.95
0.92
1.10
2.01
7.17
1.23
1.25
0.97
ppm

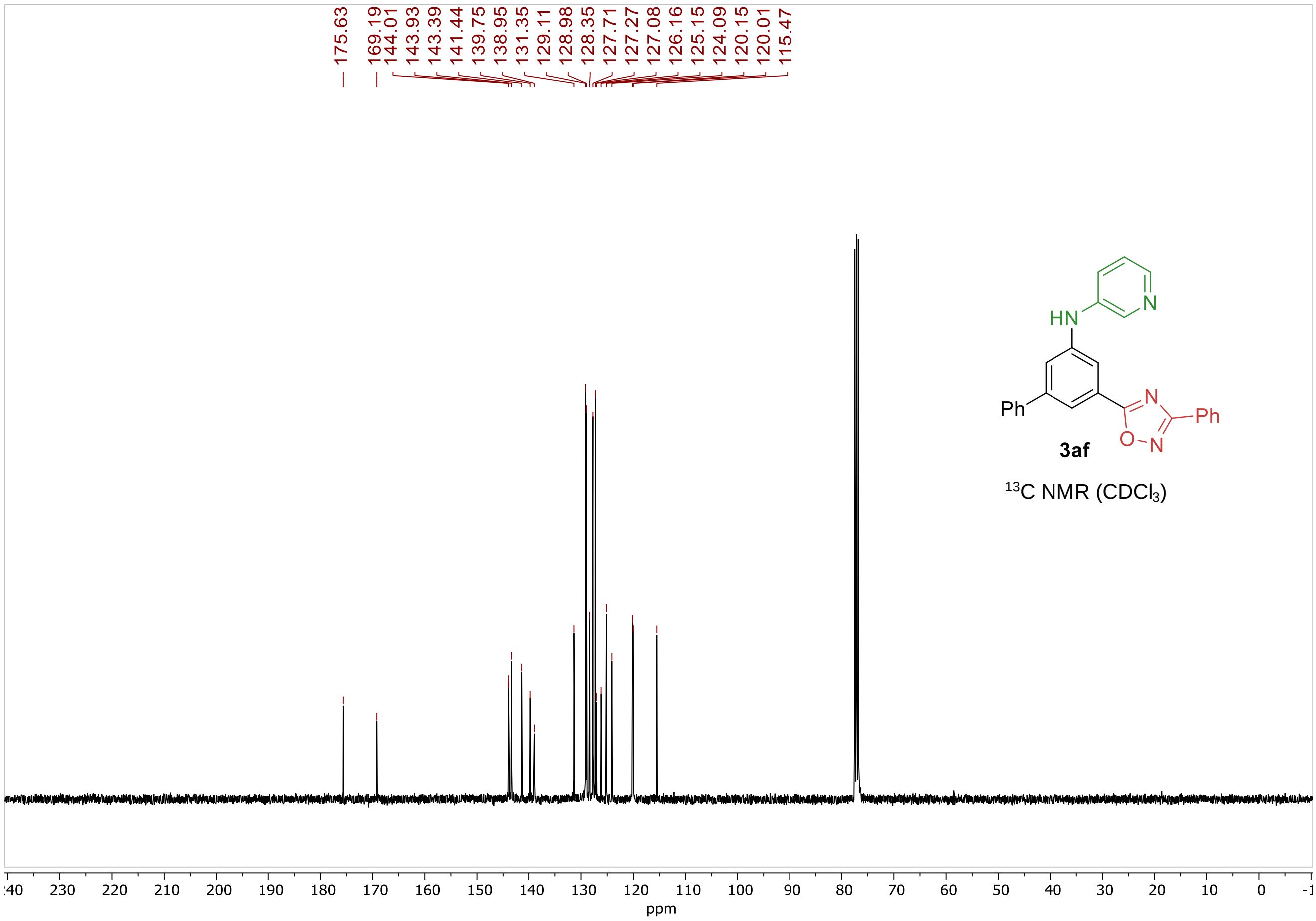
175.63
169.19
144.01
143.93
143.39
141.44
139.75
138.95
131.35
129.11
128.98
128.35
127.71
127.27
127.08
126.16
125.15
124.09
120.15
120.01
115.47
HN
N
Ph
N
Ph
O
N
3af
^{13}C NMR (CDCl3)
ppm

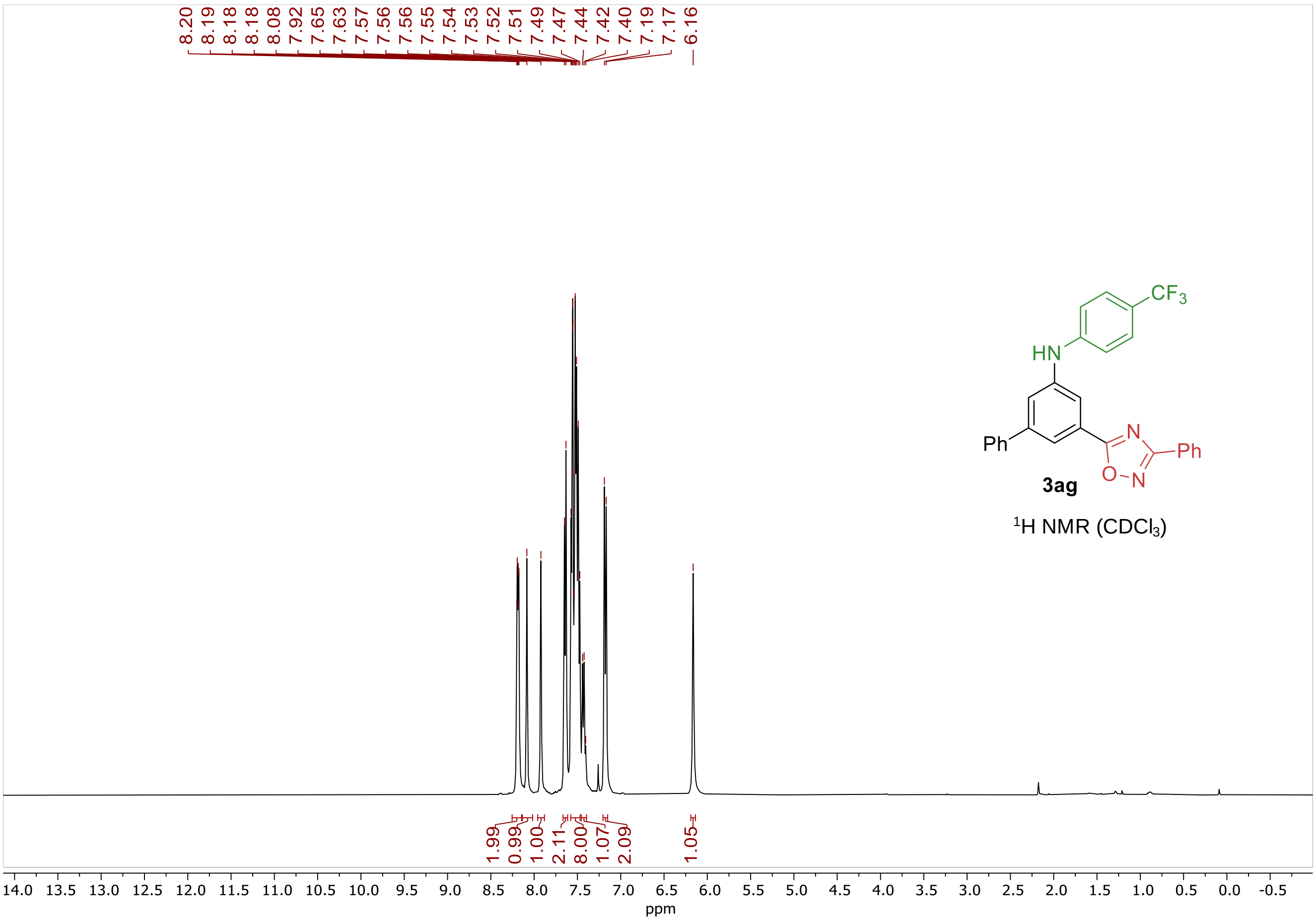
8.20 8.19 8.18 8.18 8.08 7.92 7.65 7.63 7.57 7.56 7.56 7.55 7.54 7.53 7.52 7.51 7.49 7.47 7.44 7.42 7.40 7.19 7.17 6.16
1.99 0.99 1.00 2.11 8.00 1.07 2.09 1.05
CF3
HN
Ph
N
O
N
Ph
3ag
1H NMR (CDCl3)
14.0 13.5 13.0 12.5 12.0 11.5 11.0 10.5 10.0 9.5 9.0 8.5 8.0 7.5 7.0 6.5 6.0 5.5 5.0 4.5 4.0 3.5 3.0 2.5 2.0 1.5 1.0 0.5 0.0 -0.5
ppm

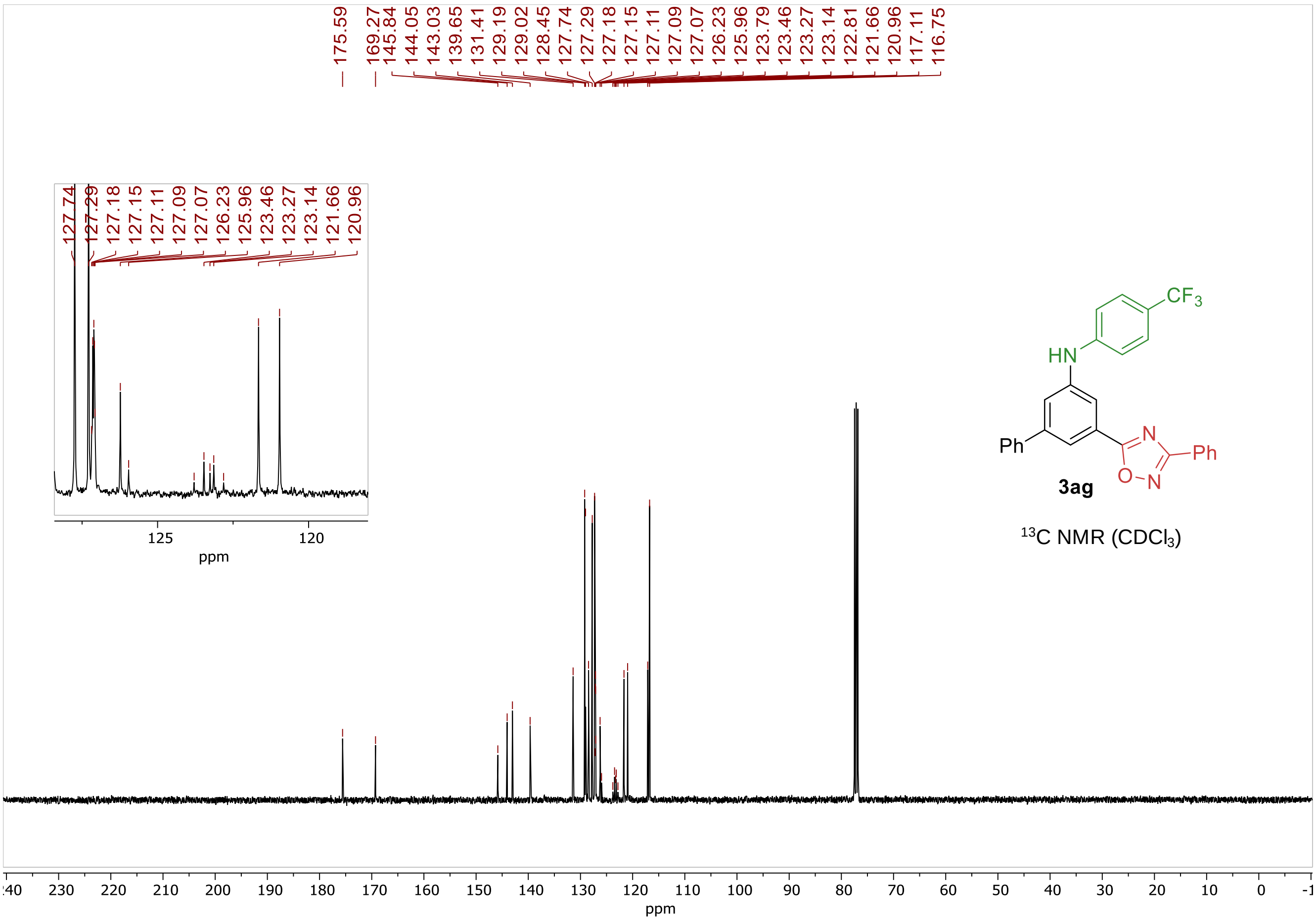
175.59 169.27 145.84 144.05 143.03 139.65 131.41 129.19 129.02 128.45 127.74 127.29 127.18 127.15 127.11 127.09 127.07 126.23 125.96 123.79 123.46 123.27 123.14 122.81 121.66 120.96 117.11 116.75
127.74 127.29 127.18 127.15 127.11 127.09 127.07 126.23 125.96 123.46 123.27 123.14 121.66 120.96
125 120
ppm
CF3
HN
Ph
N
O
N
Ph
3ag
13C NMR (CDCl3)
240 230 220 210 200 190 180 170 160 150 140 130 120 110 100 90 80 70 60 50 40 30 20 10 0 -1
ppm

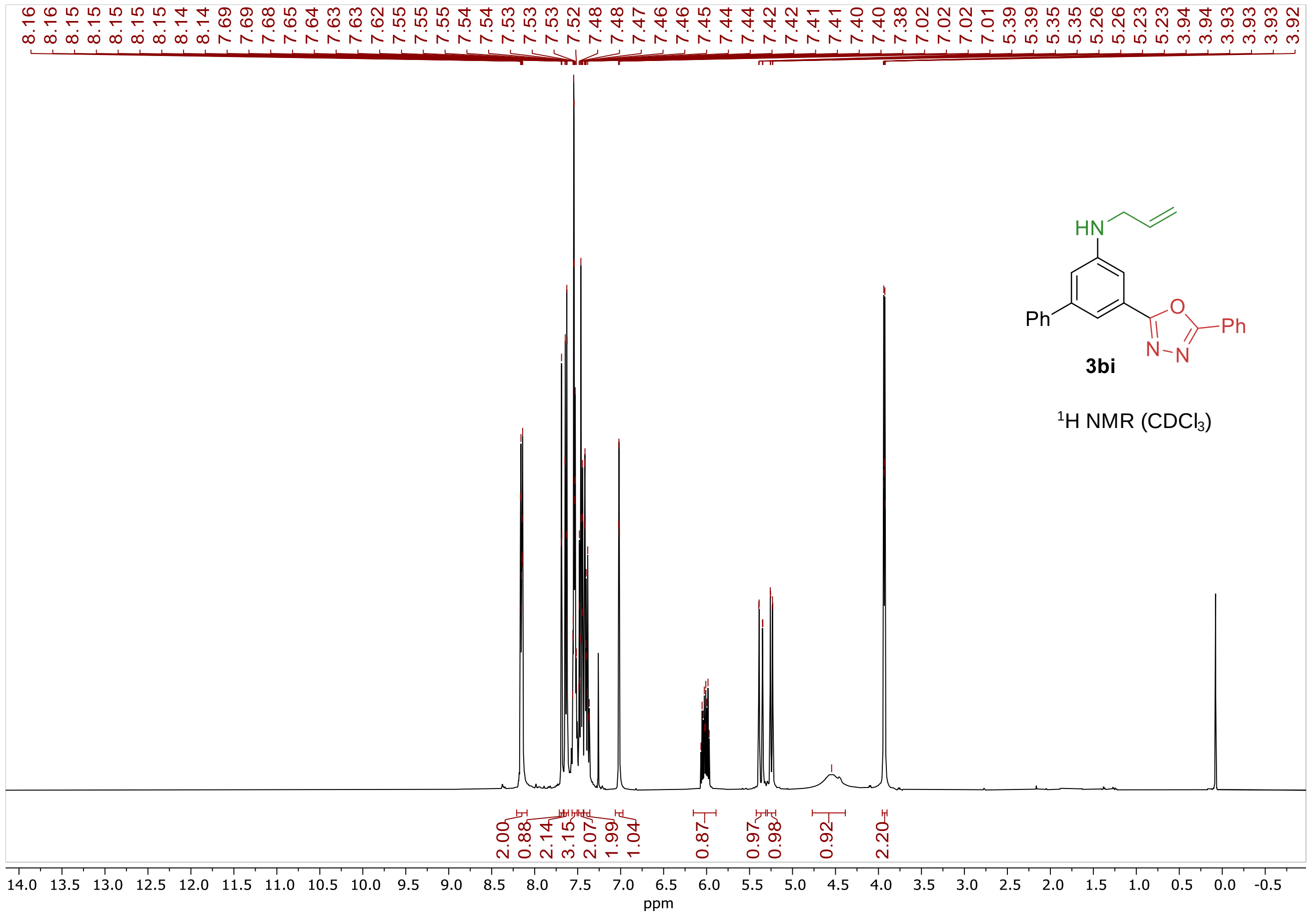
8.16
8.16
8.15
8.15
8.15
8.15
8.15
8.14
8.14
7.69
7.68
7.65
7.64
7.63
7.63
7.62
7.55
7.55
7.55
7.54
7.54
7.53
7.53
7.53
7.52
7.48
7.48
7.47
7.46
7.46
7.45
7.44
7.44
7.42
7.42
7.41
7.41
7.40
7.40
7.38
7.02
7.02
7.02
7.01
5.39
5.39
5.35
5.35
5.26
5.26
5.23
5.23
3.94
3.94
3.93
3.93
3.93
3.92
HN
Ph
O
Ph
N
N
3bi
1H NMR (CDCl3)
2.00
0.88
2.14
3.15
2.07
1.99
1.04
0.87
0.97
0.98
0.92
2.20
ppm

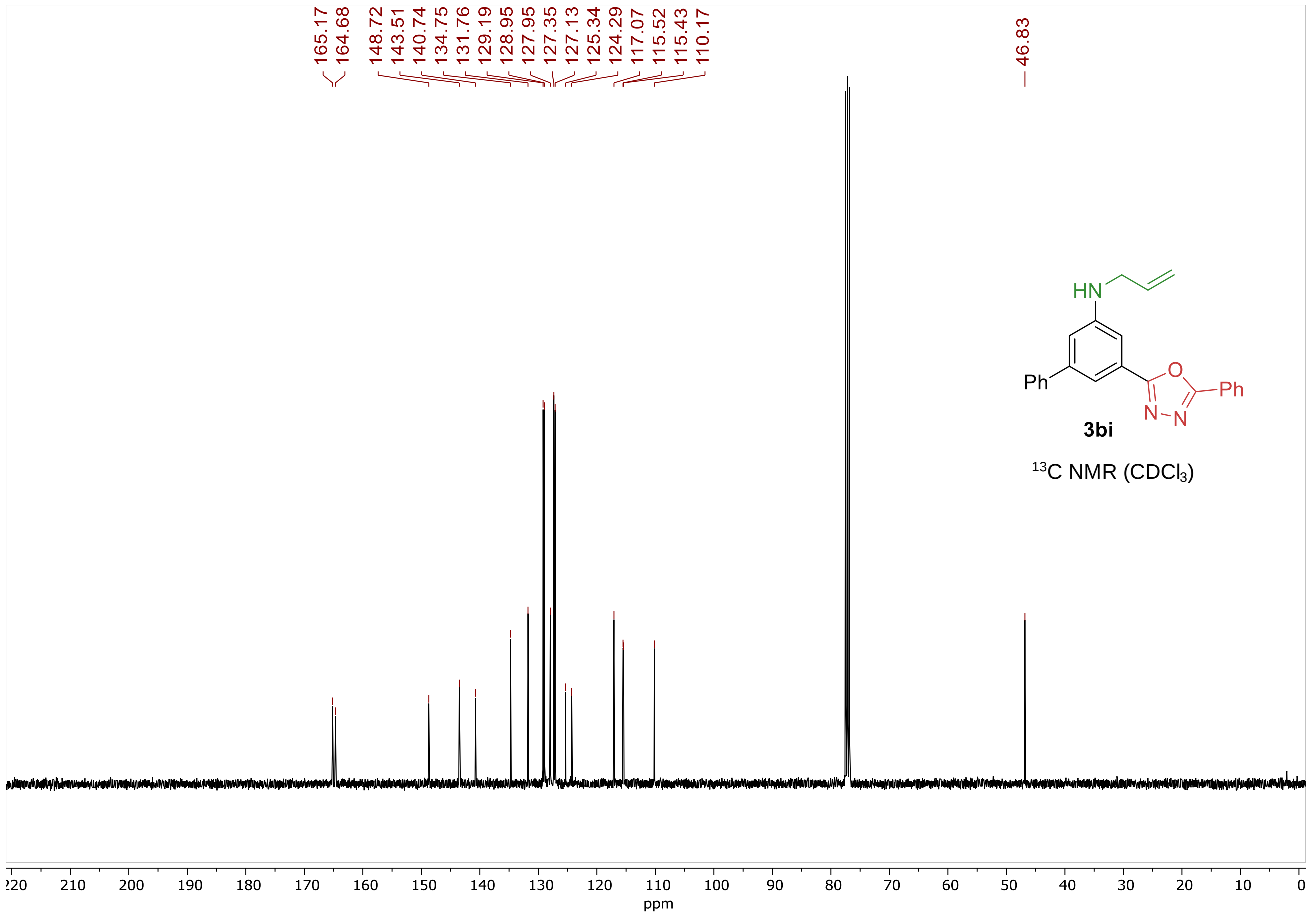
165.17
164.68
148.72
143.51
140.74
134.75
131.76
129.19
128.95
127.95
127.35
127.13
125.34
124.29
117.07
115.52
115.43
110.17
46.83
HN
Ph
O
Ph
N
N
3bi
13C NMR (CDCl3)
ppm

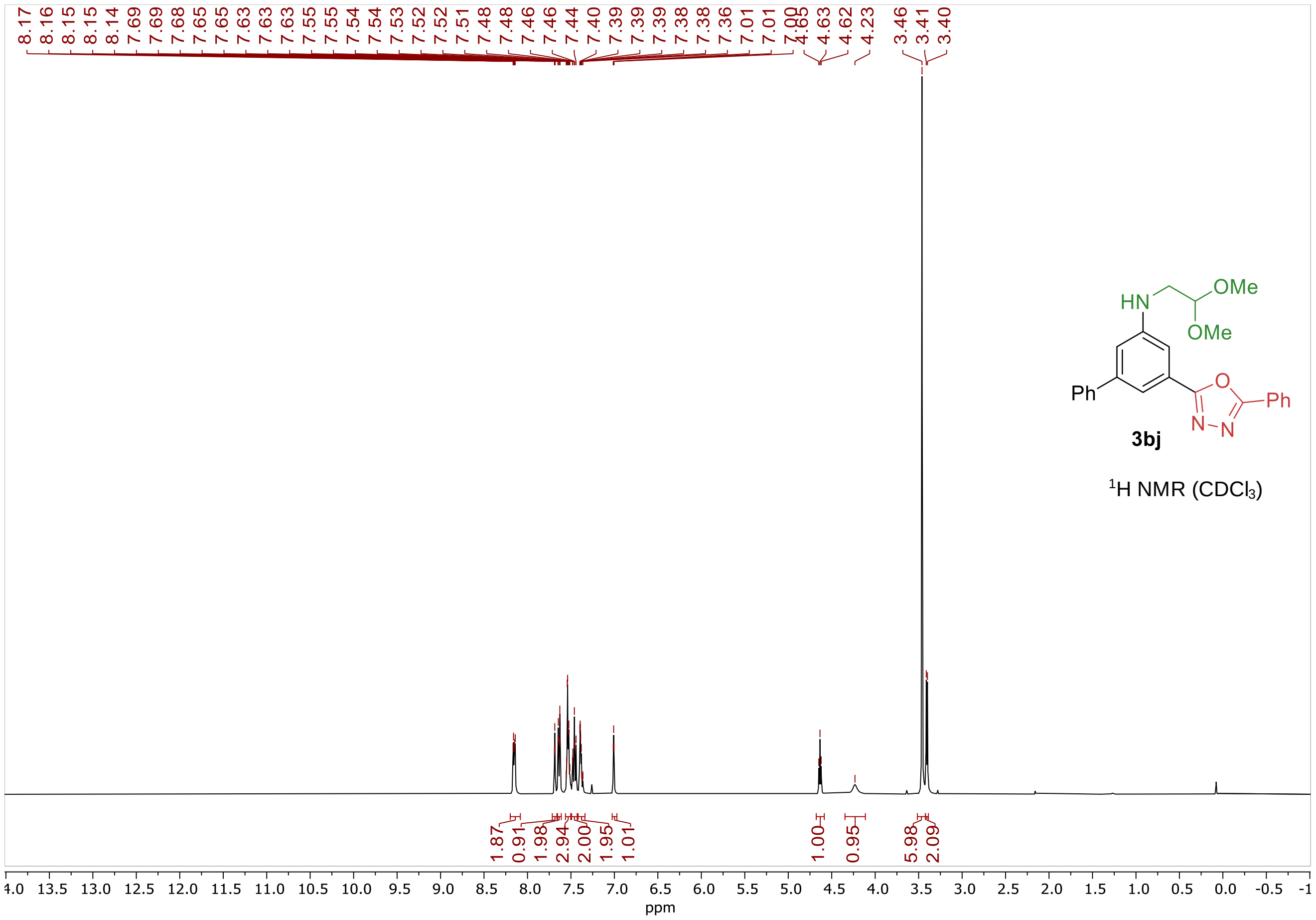
8.17 8.16 8.15 8.15 8.14 7.69 7.69 7.68 7.65 7.65 7.63 7.63 7.63 7.55 7.55 7.54 7.54 7.53 7.52 7.52 7.51 7.48 7.48 7.46 7.46 7.44 7.40 7.39 7.39 7.39 7.38 7.38 7.36 7.01 7.01 7.00 4.65 4.63 4.62 4.23 3.46 3.41 3.40
1.87 0.91 1.98 2.94 2.00 1.95 1.01 1.00 0.95 5.98 2.09
ppm
HN
OMe
OMe
Ph
O
Ph
N
N
3bj
^{1}H NMR ($CDCl_3$)

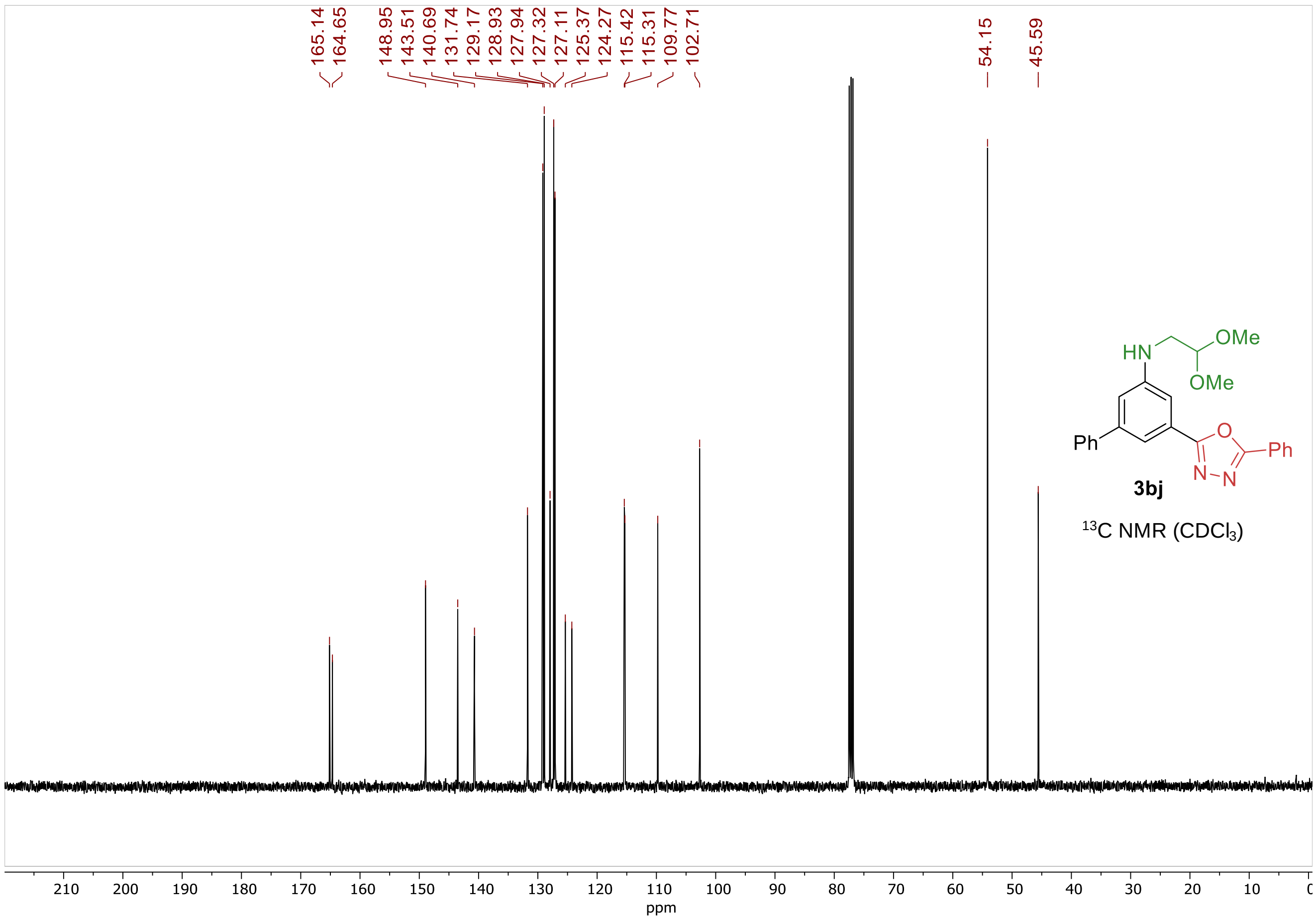
165.14 164.65 148.95 143.51 140.69 131.74 129.17 128.93 127.94 127.32 127.11 125.37 124.27 115.42 115.31 109.77 102.71 54.15 45.59
ppm
HN
OMe
OMe
Ph
O
Ph
N
N
3bj
^{13}C NMR ($CDCl_3$)

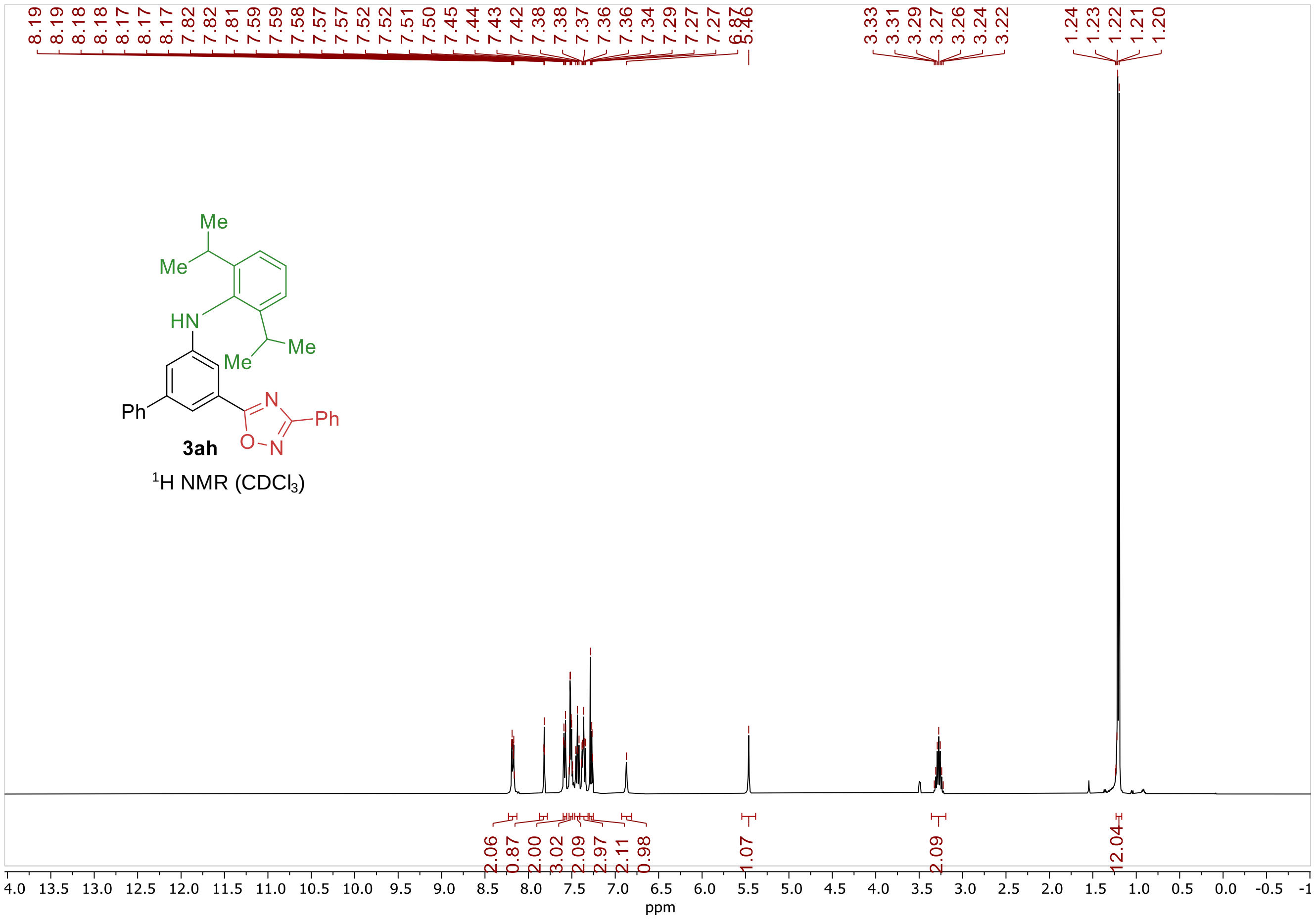
3ah
1H NMR (CDCl3)
ppm

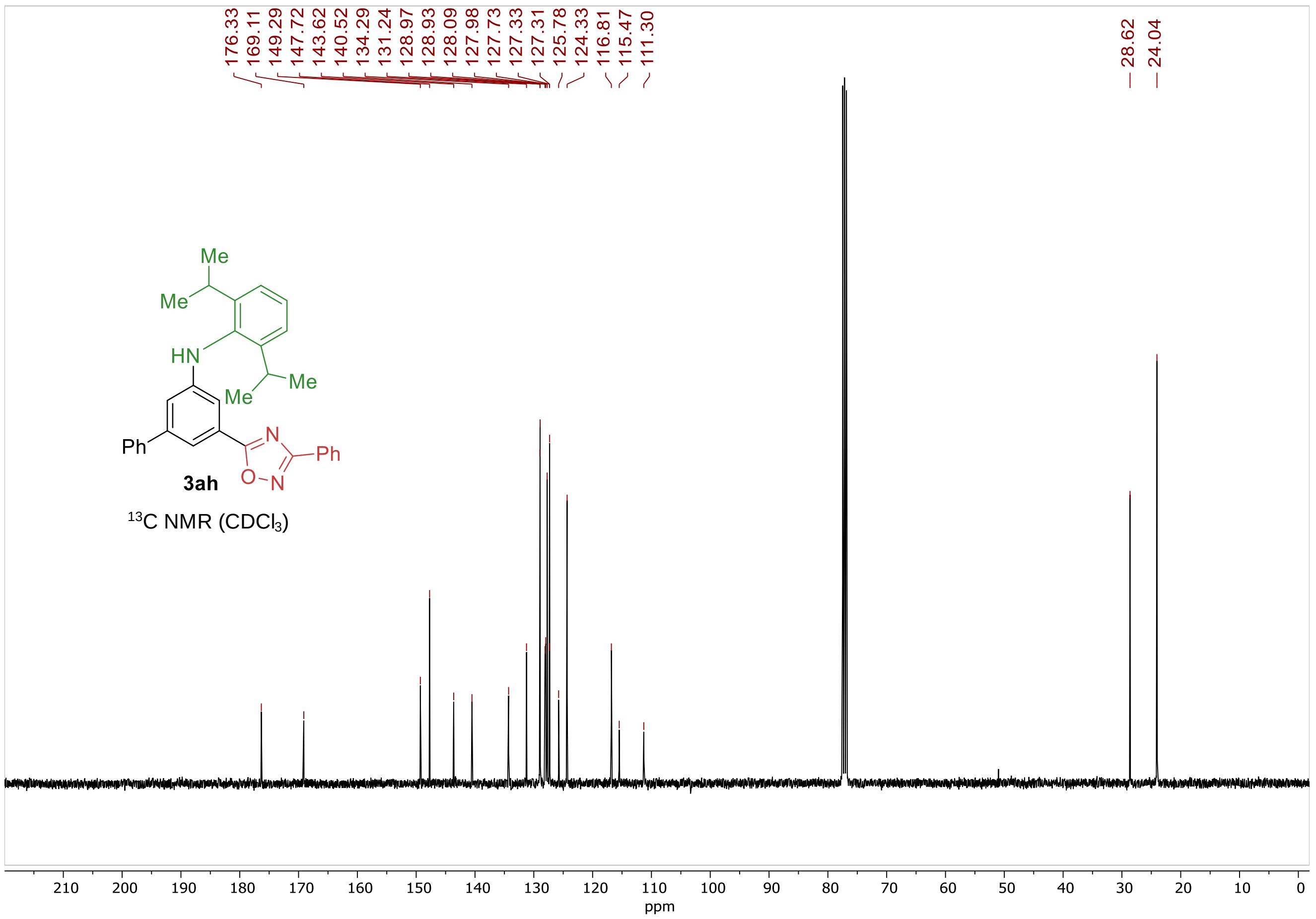
3ah
13C NMR (CDCl3)
ppm

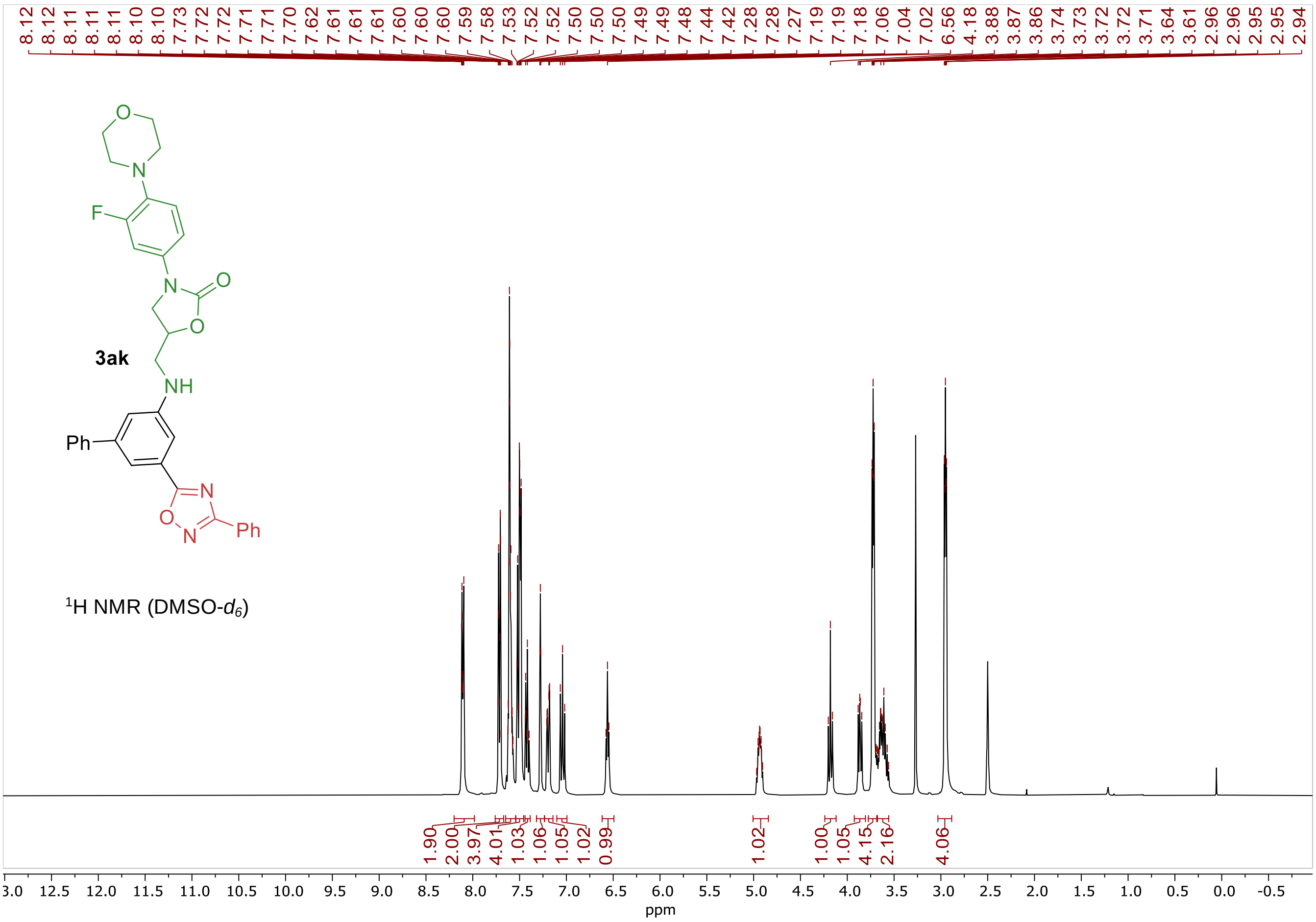
8.12
8.12
8.11
8.11
8.11
8.10
7.73
7.72
7.72
7.71
7.71
7.70
7.62
7.61
7.61
7.61
7.60
7.60
7.60
7.59
7.58
7.53
7.52
7.52
7.50
7.50
7.49
7.49
7.48
7.44
7.42
7.28
7.28
7.27
7.19
7.19
7.18
7.06
7.04
7.02
6.56
4.18
3.88
3.87
3.86
3.74
3.73
3.72
3.72
3.71
3.64
3.61
2.96
2.96
2.95
2.95
2.94
3ak
NH
Ph
F
O
N
Ph
1H NMR (DMSO-d6)
1.90
2.00
3.97
4.01
1.03
1.06
1.05
1.02
0.99
1.02
1.00
1.05
4.15
2.16
4.06
ppm

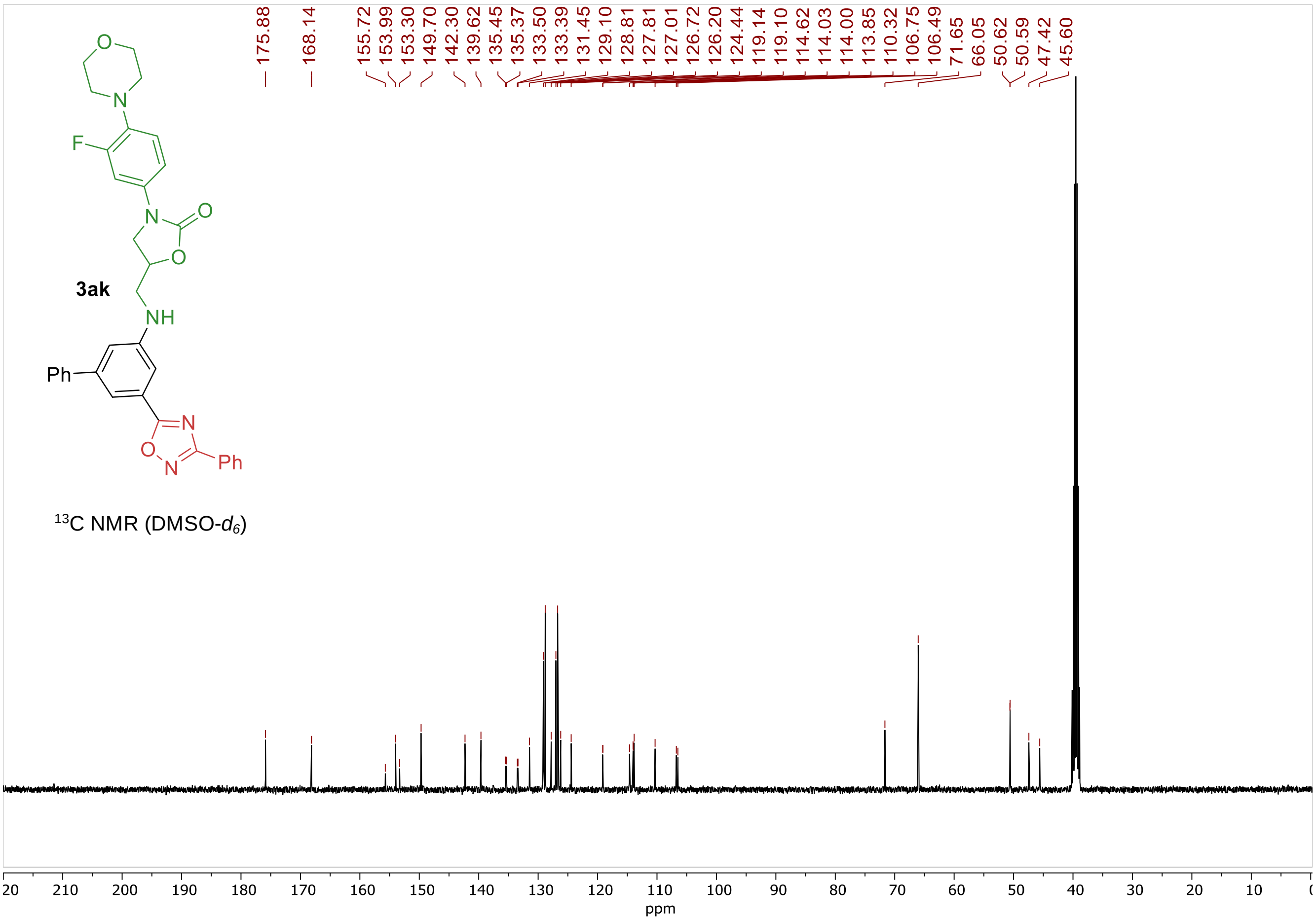
175.88
168.14
155.72
153.99
153.30
149.70
142.30
139.62
135.45
135.37
133.50
133.39
131.45
129.10
128.81
127.81
127.01
126.72
126.20
124.44
119.14
119.10
114.62
114.03
114.00
113.85
110.32
106.75
106.49
71.65
66.05
50.62
50.59
47.42
45.60
3ak
NH
Ph
F
13C NMR (DMSO-d6)
ppm
